# VIRMA-mediated m^6^A modification regulates forebrain formation through modulating ribosome biogenesis

**DOI:** 10.1126/sciadv.adq9643

**Published:** 2025-06-27

**Authors:** Min Wu, Xiaoli Wu, Haifeng Sun, Wen Wang, Leyi Zhang, Xia Liu, Yifan Zhang, Xinning Zhang, Jun Liu, Bin Shen, Tao Zhou

**Affiliations:** ^1^ Shenzhen Neher Neural Plasticity Laboratory, Shenzhen Key Laboratory of Drug Addiction, the Brain Cognition and Brain Disease Institute, Shenzhen Institutes of Advanced Technology, Chinese Academy of Sciences, Shenzhen 518055, China.; ^2^ Shenzhen-Hong Kong Institute of Brain Science-Shenzhen Fundamental Research Institutions, Shenzhen 518055, China.; ^3^ CAS Key Laboratory of Brain Connectome and Manipulation, Chinese Academy of Sciences, Shenzhen 518055, China.; ^4^ Faculty of Life and Health Sciences, Shenzhen University of Advanced Technology, Shenzhen 518106, China.; ^5^ University of Chinese Academy of Sciences, Beijing 100049, China.; ^6^ State Key Laboratory of Reproductive Medicine and Offspring Health, Women’s Hospital of Nanjing Medical University, Nanjing Maternity and Child Health Care Hospital, Gusu School, Nanjing Medical University, Nanjing 211166, China.; ^7^ State Key Laboratory of Protein and Plant Gene Research, School of Life Sciences, Peking-Tsinghua Center for Life Sciences, Peking University, Beijing 100871, China.

## Abstract

*N*^6^-Methyladenosine (m^6^A) modification plays crucial roles in tissue development and homeostasis. However, the mechanisms underlying cellular adaptation of m^6^A modification and their impact on protein synthesis machinery remain unclear. VIRMA, the largest and evolutionarily conserved core of the m^6^A methyltransferase complex, is highly expressed in the embryonic brain and various cancers. Here, we demonstrate that VIRMA-mediated m^6^A modification is essential for active ribosome biogenesis. VIRMA depletion destabilizes the entire writer complex and reduces m^6^A levels, leading to decreased proliferation and increased apoptosis of neural progenitor/stem cells, ultimately causing severe forebrain developmental defects. Mechanistically, VIRMA depletion impairs ribosome biogenesis by inhibiting mRNA decay, triggering a p53-dependent stress response and compromising global protein synthesis. These findings extend to some cancer cells, suggesting a potential conservation of this mechanism. Overall, our study reveals the critical role of m^6^A in adapting protein synthesis machinery during brain development.

## Teaser

VIRMA-mediated m^6^A modification orchestrates ribosome biogenesis, shaping brain development through protein synthesis control.

## INTRODUCTION

*N*^6^-Methyladenosine (m^6^A) is the most prevalent modification in eukaryotic mRNAs, being regulated by methyltransferases and demethyltransferases. Comprehensive studies have extensively documented the various roles of m^6^A in mRNA metabolism, ranging from nuclear processing to cytoplasmic translation and decay, through interactions with various m^6^A binding proteins (*1*–*3*). One of the most well-characterized functions of m^6^A is its involvement in facilitating mRNA degradation. It is well established that YTH *N*^6^-methyladenosine RNA binding protein 2 (YTHDF2), a selective m^6^A reader, promotes the degradation of m^6^A-modified RNA through multiple mechanisms: by directing the bound mRNA to cellular RNA decay sites, such as processing bodies, and by facilitating endoribonucleolytic cleavage through its association with ribonuclease (RNase) P/MRP, mediated by the adaptor protein HRSP12 (*4*–*6*). Thus, m^6^A has emerged as a crucial regulator of gene expression programs and plays critical roles in diverse biological processes, including tissue development and homeostasis (*1*–*3*). Several studies have identified the essential role of m^6^A in brain development by targeting the methyltransferase components methyltransferase like 3 (METTL3) and methyltransferase like 14 (METTL14), as well as the m^6^A binding protein YTHDF2 (*7*–*11*). These studies highlight the involvement of m^6^A in the degradation of crucial transcripts linked to cell state transition or histone modification, thereby influencing the self-renewal and differentiation of neural progenitor/stem cells (NPCs). In the context of tissue development, cells adjust their protein synthesis machinery to accommodate active cell growth and proliferation, aligning with metabolic demands (*12*). Despite these insights, the specific impact of m^6^A on the modulation of protein synthesis machinery during tissue development remains largely unknown.

The deposition of m^6^A on mRNA is mediated by the multicomponent methyltransferase complex (writer), which contains the catalytic METTL3/METTL14 subunits and the auxiliary subunits [including Vir like m6A methyltransferase associated (VIRMA) (*13*), Wilms tumor 1-associated protein (WTAP) (*14*), Casitas B-lineage lymphoma-like 1 (CBLL1) (*15*), Zinc finger CCCH-type containing 13 (ZC3H13) (*16*), and others] (*17*). Among these subunits, VIRMA serves as the largest and core scaffold component, having three of the four RNA binding sites within the writer complex (*18*). Notably, VIRMA, in collaboration with WTAP, counteracts the double-stranded DNA binding of the METTL3/METTL14 subunits, thereby maintaining their m^6^A methylation activity (*19*). This highlights the indispensable role of VIRMA in the activity of the writer complex. In addition, VIRMA is a notable component of the highly conserved VIRMA-WTAP-CBLL1-ZC3H13 complex found in metazoans (*20*). Its presence within this complex suggests its engagement in multicellular processes, such as cell proliferation, differentiation, and organization. According to mouse Encyclopedia of DNA Elements (ENCODE) transcriptome data (*21*), VIRMA exhibits higher expression in the embryonic brain compared to the adult brain and most other tissues, suggesting its potential importance in brain development. Moreover, numerous recent studies have reported up-regulation of VIRMA in various human cancer types (*22*, *23*). These findings imply that VIRMA might serve a common functional role in maintaining active cell growth and proliferation, which are key features of both brain development and cancer. On the basis of these observations, we decided to investigate the biological function of VIRMA.

Using an *Emx1*–*Cre*-mediated conditional knockout (cKO) mouse model with specific *Virma* gene deletion in the forebrain, we investigated the role of VIRMA in brain development. Our findings revealed that VIRMA depletion had profound consequences, leading to developmental defects, reduced proliferation, and enhanced apoptosis of NPCs in the VIRMA cKO mice. Furthermore, we observed that VIRMA deletion destabilized the entire writer complex. To gain deeper insights, we conducted RNA and m^6^A sequencing, along with proteomics analysis, which revealed that VIRMA depletion resulted in a down-regulation of m^6^A levels in mRNAs, directly affecting the expression of genes involved in ribosome biogenesis. Specifically, VIRMA depletion resulted in prolonged half-lives of mRNAs associated with ribosome biogenesis, impairing crucial steps in this process. Consequently, it triggered a p53-dependent stress response, compromising global protein translation and leading to disrupted cell growth and proliferation. Ultimately, these disruptions led to severe developmental defects. To assess the relevance of these findings in a broader context, we conducted preliminary analyses by including human breast cancer cells (MCF7) and cervical cancer cells (HeLa). We observed similar ribosome biogenesis defects in VIRMA-depleted cancer cells. In summary, our study provides insights into the coordination between mRNA m^6^A modification and protein synthesis machinery. We demonstrate that VIRMA-mediated m^6^A plays a crucial role in regulating the turnover of transcripts involved in ribosome biogenesis, thereby maintaining active ribosome biogenesis during normal brain development and potentially in some cancers as well.

## RESULTS

### VIRMA deficiency impairs forebrain development, reduces proliferation, and enhances apoptosis of NPCs

**Fig. 1.**
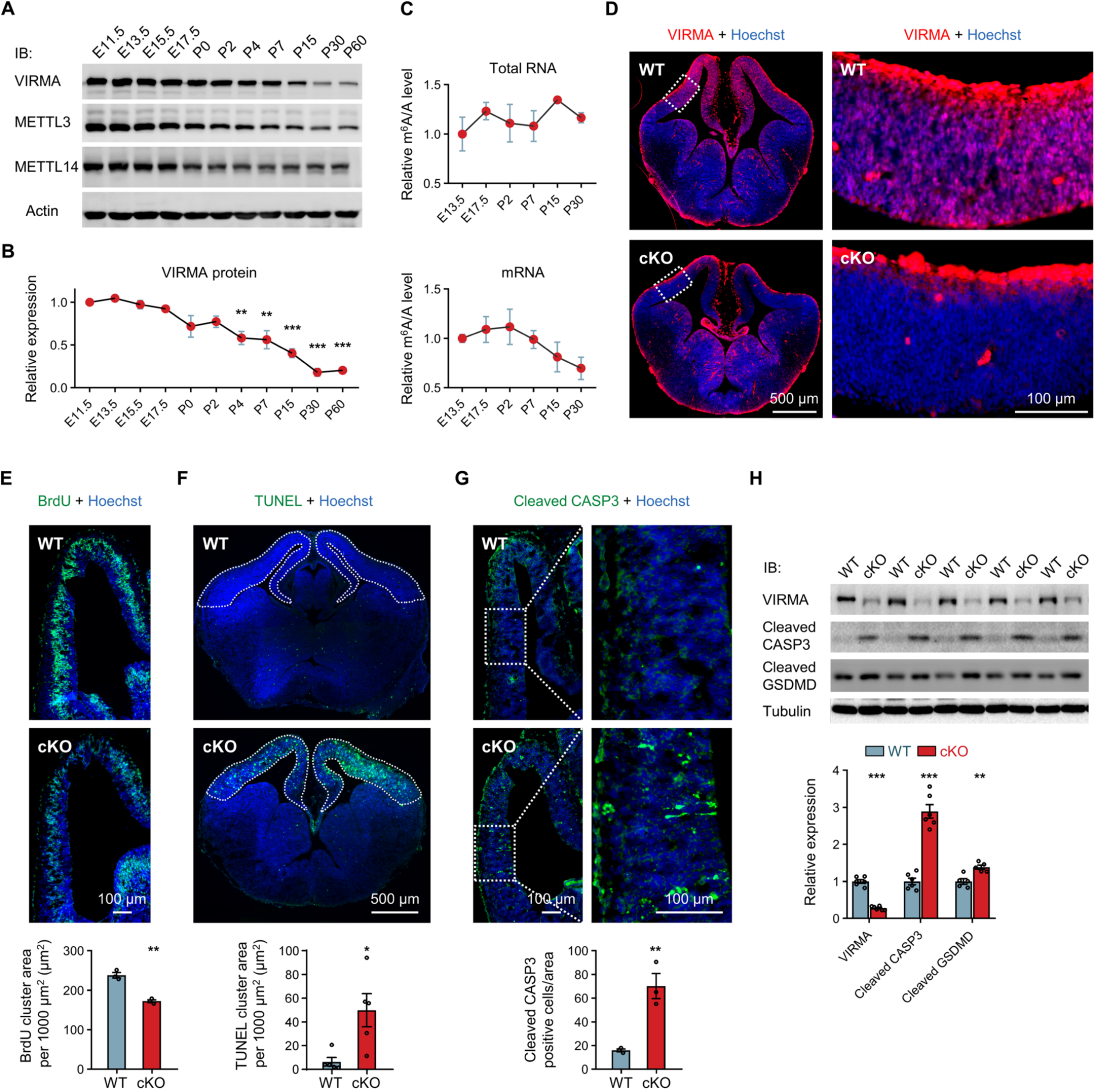
VIRMA depletion reduces forebrain NPCs proliferation and enhances apoptosis during the early embryonic stage. (**A**) Temporal expression profile of VIRMA, METTL3, and METTL14 protein in the mouse forebrain from E11.5 to P60. IB, immunoblotting. (**B**) Quantification of VIRMA protein abundance in (A). *N* = 2 samples for each stage. (**C**) LC-MS/MS analysis showing the temporal profile of m^6^A/A level in total RNA and mRNA isolated from mouse forebrain at different developmental stage. *N* = 3 samples for each stage. (**D**) Immunostaining for VIRMA in the brains of VIRMA WT and cKO mice at E13.5. Regions enclosed by white dashed boxes are shown at a higher magnification. (**E**) Assessment of cell proliferation through BrdU immunostaining on coronal brain sections from VIRMA WT and cKO mice at E13.5. The area (μm^2^ per 1000 μm^2^) with BrdU signal was reduced in the forebrain of VIRMA cKO mice. *N* = 3 mice for each genotype. (**F**) Evaluation of cell apoptosis through TUNEL assay. White dashed lines indicate the forebrain area used for quantification. The area with TUNEL signal was markedly increased in VIRMA cKO mice. *N* = 5 mice for each genotype. (**G**) Immunostaining of coronal brain sections with an anti-cleaved CASP3 antibody. The number of cleaved CASP3-positive cells in each forebrain section was manually counted. *N* = 3 mice for each genotype. (**H**) WB analysis showing the expression levels of apoptotic markers in the forebrain of VIRMA WT and cKO mice at E13.5. *N* = 5 to 6 mice for each genotype. Nuclei were counterstained with Hoechst in (D) to (G). One-way analysis of variance (ANOVA) followed by Dunnett’s multiple comparisons test in (B) and (C), *P < 0.05, **P < 0.01, and ***P < 0.001, compared to the first time point. Unpaired Student’s *t* test in (E) to (H), compared with WT mice. Data are means ± SEM.

We first investigated the temporal expression profile of VIRMA protein across different stages of mouse brain development. Western blot (WB) analysis revealed high expression levels of VIRMA during embryonic stages, which notably diminished in the adult brain (Fig. 1, A and B). We also observed high expression levels of METTL3 and METTL14 during embryonic brain development (Fig. 1A). In addition, a publicly accessible single-cell RNA-sequencing (RNA-seq) dataset of NPCs and their progeny (*24*) demonstrated a notable expression of VIRMA in NPCs (fig. S1A). These findings prompted us to explore whether the m^6^A levels undergo dynamic changes during brain development. We used liquid chromatography–tandem mass spectrometry (LC-MS/MS) to measure m^6^A levels. Our results indicated that, similar to VIRMA, the m^6^A levels in mRNA appeared higher during the embryonic stages and then gradually decreased postnatally (Fig. 1C). In contrast, total RNA m^6^A levels remained relatively stable throughout development. These findings suggest that VIRMA and m^6^A play essential and dynamic roles in the intricate process of brain development.

To further investigate the functional role of VIRMA in early brain development, we then conditionally deleted *Virma* using the *Emx1*–*Cre;Virma^flox/flox^* model (VIRMA cKO) (fig. S1, B and C). The *Emx1*–*Cre* transgene expressed Cre recombinase in the progenitor cells of dorsal telencephalon starting at embryonic day 10.5 (E10.5) to specifically delete VIRMA alleles in the forebrain (including cortex and hippocampus) of VIRMA cKO mice (*25*). *Virma^flox/flox^* or *Virma^flox/+^* littermates were used as wild-type controls (VIRMA WT). Immunostaining and WB analysis of E13.5 brains confirmed VIRMA deletion in the forebrain of VIRMA cKO mice (Fig. 1D and fig. S1D). VIRMA cKO mice exhibited a significantly reduced rate of body weight growth, with an approximate half size compared to WT littermates by postnatal day 20 (P20) (fig. S1, E and F). In addition, most cKO mice died prematurely with a median survival time of 25 days (fig. S1G). These findings highlight the essential role of VIRMA in the mammalian brain for survival.

We then examined the structure of forebrain. Starting from birth, the brains of VIRMA cKO mice were smaller, and histological analysis of brain sections with Hoechst staining unveiled the complete absence of the hippocampus and thinning of the cortex in VIRMA cKO mice (fig. S2, A and B). We also stained for dentate gyrus granule cell marker prospero homeobox 1 (PROX1) and astrocytic marker glial fibrillary acidic protein (GFAP), observing a significant decrease in their signals in the forebrain of VIRMA cKO mice, along with a disruption of the hippocampal structure (fig. S2, C and D). Further examination of E15.5 VIRMA cKO mice brains showed a moderate reduction in cortical length and width (fig. S3A). Hematoxylin and eosin (H&E) staining of E15.5 brain sections indicated ventricle enlargement and a substantial decrease in cortical thickness in VIRMA cKO brains compared to WT controls (fig. S3B). To further characterize the impact of VIRMA deletion on cortical development, we examined neuronal subtypes by immunostaining for cortical layer markers COUP-TF-interacting protein 2 (CTIP2) and special AT-rich sequence binding protein 2 (SATB2), as well as neural stem cells labeled by paired box 6 (PAX6). The number of CTIP2-positive, SATB2-positive, and PAX6-positive cells all significantly decreased, further highlighting the developmental damage in the forebrain of VIRMA cKO mice (fig. S3, C and D). Together, these results demonstrate severe impairment of forebrain development in VIRMA cKO mice.

We aimed to determine whether the developmental abnormalities observed in VIRMA cKO mice could be attributed to compromised cell proliferation, activation of cell death pathways, or a combination of both. We first examined the proliferation capacity of cells at E13.5, a developmental stage dominated by NPCs within the forebrain. Immunostaining revealed a marked reduction in bromodeoxyuridine (BrdU)–positive signals in the forebrain of VIRMA cKO mice (Fig. 1E). This result indicates that VIRMA deficiency hinders the proliferative capacity of NPCs. Subsequently, we examined cell death at E13.5. We detected robust terminal deoxynucleotidyl transferase–mediated deoxyuridine triphosphate nick end labeling (TUNEL)–positive signals in the forebrain of VIRMA cKO mice (Fig. 1F). This observation indicates that numerous VIRMA-depleted cells undergo DNA fragmentation, indicative of apoptosis. We also stained for cleaved caspase 3 (CASP3), a major execution protein in apoptotic pathways, and noted a significant increase in cleaved CASP3 signals within cKO mice (Fig. 1G). WB analysis corroborated these findings, revealing significantly elevated levels of cleaved CASP3, as well as cleaved gasdermin D (GSDMD) at residue D88, which has been shown to occur in apoptotic cells (*26*, *27*) (Fig. 1H). These results collectively suggest that VIRMA deficiency leads to abnormal activation of apoptosis in mice forebrain. In summary, our results reveal that VIRMA depletion primarily induces defects in forebrain development by affecting both NPC proliferation and apoptosis during the embryonic stage. This profile is distinct from that observed in METTL3 or METTL14 cKO mouse models (*8*–*11*), where either reduced proliferation or enhanced apoptosis of cells was noted, implying the involvement of different mechanisms.

### VIRMA deficiency reduces m^6^A levels and enhances the stability of m^6^A-modified mRNA

**Fig. 2.**
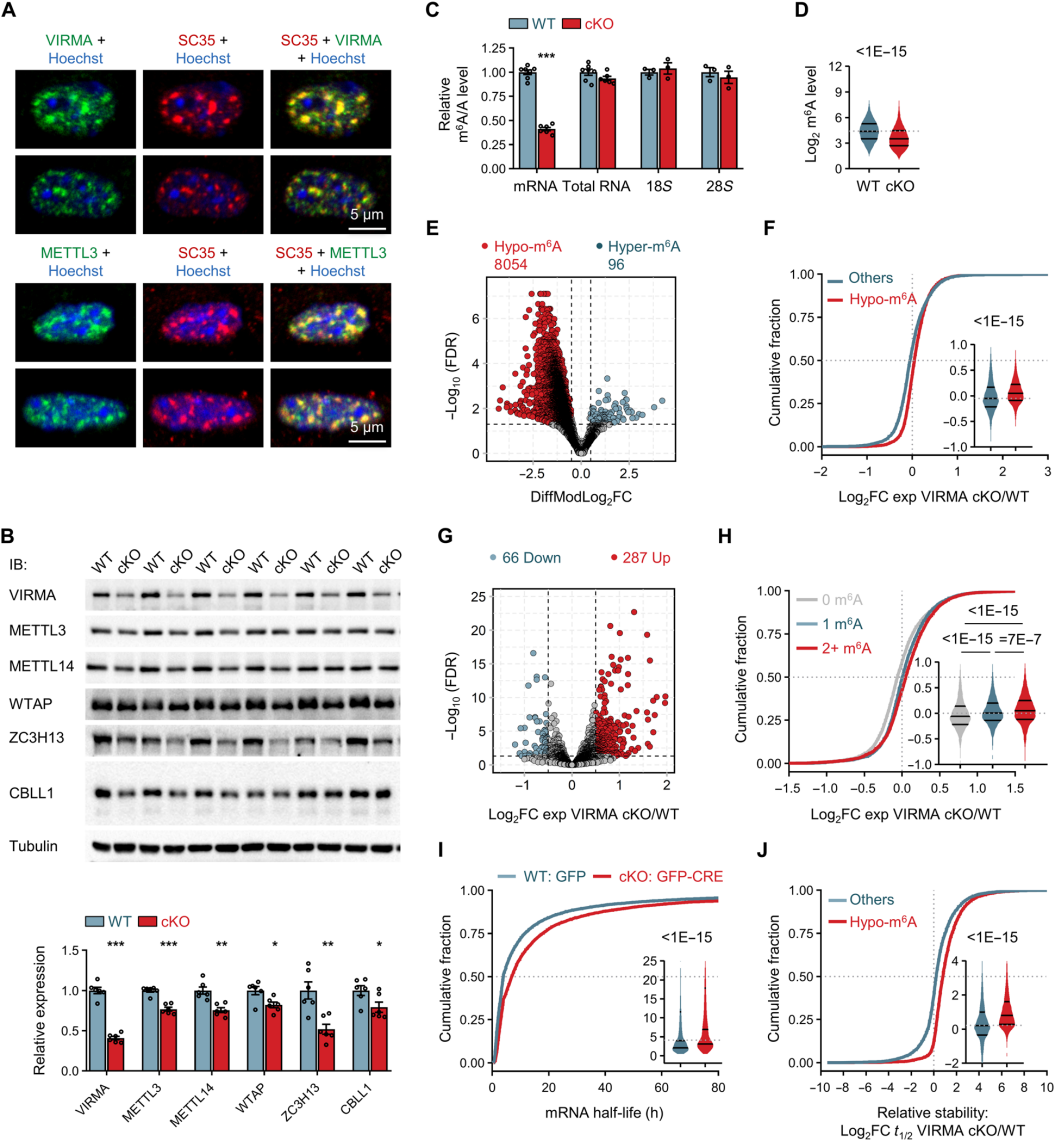
VIRMA deficiency reduces m^6^A levels and enhances the stability of m^6^A-modified mRNA in NPCs. (**A**) Immunostaining showing VIRMA localization to SC35-positive nuclear speckles in cultured NPCs. (**B**) Protein levels of methyltransferase complex components in E13.5 forebrain. *N* = 6 samples per genotype. (**C**) LC-MS/MS analysis of m^6^A/A levels in different RNAs from E13.5 forebrain. *N* = 3 to 7 samples per genotype. (**D**) Violin plots showing mRNA m^6^A levels from MeRIP-m^6^A-seq. *P* value by the Wilcoxon signed-rank test. (**E**) Volcano plot showing differentially methylated m^6^A peaks upon VIRMA depletion. Hypo-m^6^A, hypomethylated; Hyper-m^6^A, hypermethylated. (**F**) Cumulative distributions and violin plots (inset) showing the difference in expression level FC [Log_2_FC (cKO/WT)] between mRNAs with Hypo-m^6^A peaks and “Others” mRNAs. (**G**) Volcano plot showing the expression level FC [Log_2_FC (cKO/WT)] of mRNAs with Hypo-m^6^A peaks. Significantly up-regulated or down-regulated mRNAs are highlighted. (**H**) Cumulative distributions and violin plots (inset) showing the expression change [Log_2_FC (cKO/WT)] of mRNAs with 0, 1, or 2+ m^6^A sites. *P* values by the Kruskal-Wallis test followed by Dunn’s multiple comparisons test. (**I**) Cumulative distributions and violin plots (inset) showing mRNA half-lives in cultured NPCs. *P* value by the Wilcoxon signed-rank test. h, hours. (**J**) Cumulative distributions and violin plots (inset) showing the half-lives change [Log_2_FC (cKO/WT)] for mRNAs with Hypo-m^6^A peaks and “Others” mRNAs. “Others” in (F) and (J) refers to mRNAs with unchanged or increased m^6^A levels after VIRMA depletion. Four E13.5 forebrain samples per genotype were used for MeRIP-m^6^A-seq & RNA-seq (D to H), and four cultured NPCs samples per genotype at each time point were used in (I) and (J). Unpaired Student’s *t* test in (B) and (C), *P < 0.05, **P < 0.01, and ***P < 0.001, compared with WT samples. *P* value was determined using Wilcoxon rank sum tests in (F) and (J). Data are means ± SEM.

To unravel the mechanism underlying forebrain developmental defects in VIRMA cKO mice, we first characterized the subcellular localization of VIRMA. We performed immunostaining of NPCs cultured from *Virma^flox/flox^* mouse forebrain at E13.5, a stage enriched with NPCs (fig. S4A). The cultured NPCs were infected with lentivirus expressing GFP-CRE to achieve VIRMA knockout, whereas green fluorescent protein (GFP) was used as a control (fig. S4B). Consistent with previous reports (*28*, *29*), endogenous VIRMA, akin to METTL3, predominantly colocalized with the pre-mRNA splicing factor SC35 in nuclear speckles (Fig. 2A). Nuclear speckles are known sites for methyltransferase complex activity. Intriguingly, VIRMA depletion in NPCs led to diminished accumulation of METTL3/METTL14 and other m^6^A writer components, like WTAP, ZC3H13, and CBLL1, within nucleus (fig. S5, A to G). Notably, the SC35 signal within nuclear speckles remained unaffected (fig. S5F), suggesting that VIRMA depletion may not alter nuclear speckles. We also examined the impact of VIRMA on protein levels of m^6^A writer components in the mouse forebrain at E13.5. Our results revealed a noteworthy reduction in protein levels of m^6^A writer components in VIRMA cKO mice compared to WT mice (Fig. 2B). The mRNA levels of m^6^A writer components remained unchanged or even exhibited up-regulation (fig. S5H). Collectively, these observations emphasize the essential role of VIRMA in maintaining the protein stability of the entire writer complex, in line with previous structural findings suggesting VIRMA as the core structure of the complex (*18*).

We next investigated whether VIRMA deletion might influence m^6^A methyltransferase activity in vivo. We quantified m^6^A levels in forebrain RNA from VIRMA WT and cKO mice at E13.5 using LC-MS/MS assay. VIRMA deletion led to a marked reduction of ~60% in m^6^A levels within mRNA, whereas m^6^A levels within total RNA, 18*S* ribosomal RNA (rRNA), and 28*S* rRNA remained unchanged (Fig. 2C). To further explore the transcriptome-wide implications of VIRMA on mRNA m^6^A, we conducted methylated m^6^A RNA immunoprecipitation sequencing (MeRIP-m^6^A-seq) and RNA sequencing (RNA-seq) (fig. S6A and tables S1 to S3). We identified 18,631 and 17,658 m^6^A peaks in the forebrain mRNAs of VIRMA WT and cKO mice, respectively (table S1). These peaks contained the canonical motif RRACH (R = A or G; H = A, C or U) and were mainly distributed in regions of 3′ untranslated region (3′UTR) and near stop codons (fig. S6, B and C). VIRMA depletion resulted in a decrease in total m^6^A levels and 8150 differential m^6^A peaks, with nearly 99% of these peaks significantly down-regulated (Hypo-m^6^A: 8054) and over half of the Hypo-m^6^A peaks located in the 3′UTR regions (Fig. 2, D and E; fig. S6, D and E; and table S2). In summary, our findings indicate that VIRMA is essential for preserving global m^6^A levels on mRNAs during brain development.

Subsequently, we examined whether VIRMA-mediated m^6^A modification could drive mRNA change by analyzing the transcriptome based on mRNA m^6^A modification. Our results showed that mRNAs with Hypo-m^6^A peaks (4923 genes; table S2) tended to be up-regulated in VIRMA cKO forebrain (Fig. 2F). Among the 4923 genes with Hypo-m^6^A peaks, there were significantly more up-regulated (287) than down-regulated (66) genes, indicating a clear predominance of up-regulated genes (Fig. 2G). In addition, mRNAs containing two or more m^6^A sites exhibited a much higher increase in expression upon VIRMA depletion (Fig. 2H). Reciprocally, we observed an enrichment of m^6^A occupancy in significantly up-regulated genes [false discovery rate (FDR) < 0.05] in VIRMA cKO forebrain compared to unchanged or down-regulated genes (fig. S6F). The relationship between VIRMA-mediated m^6^A modification and increased mRNA levels in VIRMA-depleted forebrain suggests that VIRMA deficiency leads to the up-regulation of target transcripts. This up-regulation of mRNAs may result from extended half-lives, given that one of the most well-characterized functions of m^6^A is its role in facilitating mRNA degradation. To measure the half-lives, we conducted RNA-seq on cultured NPCs after treatment with actinomycin D (ActD) to inhibit transcription. We observed a significant extension in mRNA half-life in VIRMA cKO NPCs (Fig. 2I). mRNAs containing two or more m^6^A sites exhibited much shorter half-lives compared to those without or with just one m^6^A site (fig. S6G). VIRMA depletion extended the half-lives of mRNAs with two or more m^6^A sites to a much higher degree (fig. S6H). In addition, mRNAs with Hypo-m^6^A peaks displayed longer half-lives than other mRNAs in VIRMA cKO NPCs (Fig. 2J). Notably, the major alternative splicing (AS) event in the forebrain remained unaffected by VIRMA depletion (fig. S6I), with only 461 different AS events identified between VIRMA WT and cKO forebrain (fig. S6J). This change in AS event is not as dominant as previous observations in VIRMA-depleted oocytes, where genes had a significantly lower exon inclusion level, and 4214 different AS events were identified (*29*). In addition, alternative polyadenylation (APA) analysis using the DaPars algorithm (*30*) revealed no changes in APA events in the VIRMA cKO forebrain (fig. S6K), in contrast to prior findings in VIRMA-depleted HeLa cells (*13*). Overall, our results highlight that VIRMA deficiency primarily enhances the stability of m^6^A-modified mRNA, with VIRMA-mediated m^6^A modification in the forebrain predominantly regulating the transcriptome via the mRNA decay pathway rather than splicing or APA.

### VIRMA deficiency alters expression of genes involved in ribosome biogenesis

**Fig. 3.**
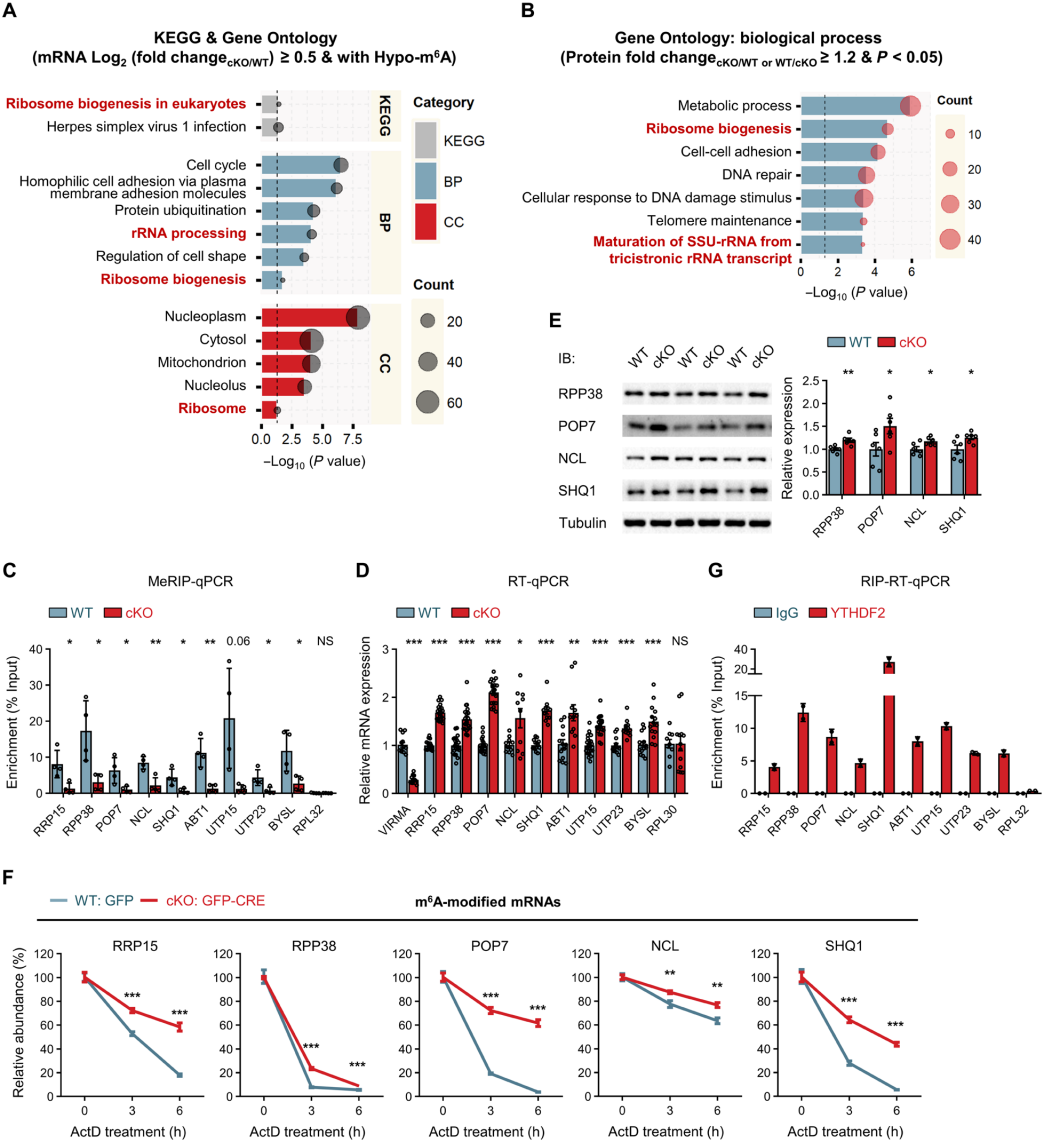
VIRMA deficiency induces profound expression changes of genes involved in ribosome biogenesis in the forebrain. (**A**) KEGG pathway and GO analyses of genes with up-regulated mRNA levels and Hypo-m^6^A peaks upon VIRMA depletion. Significantly enriched terms (*P* < 0.05, Fisher’s exact test in DAVID) are listed and ranked by *P* value. Enriched terms related to ribosome biogenesis are highlighted in red. BP, biological processes; CC, cellular components. (**B**) Significantly enriched GO terms in the biological process category for differentially expressed proteins (796 proteins) identified through proteomic analysis of E13.5 VIRMA WT and cKO forebrain, based on *P* value from Fisher’s exact test in DAVID. (**C**) MeRIP-qPCR analysis of m^6^A modifications on ribosome biogenesis mRNAs in E13.5 VIRMA WT and cKO forebrain. RPL32 was used as a negative control. *N* = 4 biologically independent experiments. (**D**) RT-qPCR confirmed the abnormal up-regulation of ribosome biogenesis genes in E13.5 cKO forebrain. The Ct values were normalized to GAPDH. *N* = 9 to 21 samples per genotype. (**E**) WB validation of the abnormal up-regulation of ribosome biogenesis genes in E13.5 cKO forebrain. *N* = 6 samples per genotype. (**F**) RT-qPCR validation of the enhanced stability of m^6^A-modified mRNAs upon VIRMA depletion in cultured NPCs. mRNA levels at 0, 3, and 6 hours after ActD treatment were normalized to RPL30 and presented as a percentage of mRNA amount at 0 hours. *N* = 6 samples per condition. (**G**) RIP combined with RT-qPCR analysis detected YTHDF2 binding to mRNAs involved in ribosome biogenesis in cultured NPCs. RPL32 was used as a negative control. Rabbit IgG was used as a control for IP. *N* = 2 biologically independent experiments. Unpaired Student’s *t* test in (C) to (F), *P < 0.05, **P < 0.01, and ***P < 0.001, compared with WT samples. Data are means ± SEM. NS, not significant.

We performed Kyoto Encyclopedia of Genes and Genomes (KEGG) pathway and Gene Ontology (GO) analyses on the up-regulated genes with Hypo-m^6^A peaks in VIRMA cKO forebrain (287 genes, as shown in table S3). The top-enriched KEGG pathway we identified is closely associated with ribosome biogenesis (Fig. 3A), a fundamental intranuclear process essential for building the protein synthesis machinery. Moreover, GO analysis revealed significant enrichment of ribosome in the cellular component (CC) category and significant enrichment in the biological process (BP) category for rRNA processing, which is a critical step in ribosome biogenesis (Fig. 3A). Similarly, GO analysis of the differentially expressed proteins resulting from the proteomic analysis of VIRMA WT and cKO forebrain also pointed to ribosome biogenesis pathway (Fig. 3B and table S4). These findings strongly suggest that VIRMA deficiency and consequent m^6^A depletion could alter the expression of genes involved in ribosome biogenesis.

We selected nine representative genes implicated in ribosome biogenesis based on the above KEGG pathway and GO analyses (Fig. 3, A and B, and tables S3 and S4) to examine their m^6^A methylation patterns (fig. S7A). These genes included ribosome RNA processing protein RRP15 (*31*–*33*), RNase MRP complex components RPP38 and POP7 (*34*–*36*), one of the most abundant nucleolar protein nucleolin (NCL) (*37*–*39*), the pre-rRNA processing factor SHQ1 (*40*, *41*), small-subunit (SSU) processome components ABT1, UTP15, UTP23, and BYSL (*42*, *43*). RNase MRP is essential for precursor rRNA processing at a defined site within the internal transcribed spacer region 1 (ITS1) in eukaryotic cells, disruption of which leads to pre-rRNA accumulation (*34*, *36*, *44*). NCL plays a role in several stages of ribosome biogenesis, including ribosomal DNA synthesis and ribosomal assembly, and is crucial for the initial step of rRNA processing by interacting with a highly conserved RNA sequence within the precursor rRNA 5′ external transcribed spacer (5′-ETS) (*37*–*39*). SHQ1 serves as an essential assembly factor for eukaryotic box H/ACA small nucleolar ribonucleoproteins (snoRNPs), playing a crucial role in the extensive pseudouridylation of rRNA and contributing to rRNA processing (*40*, *41*). The SSU processome is a large ribonucleoprotein complex essential for the biogenesis of the 18*S* rRNA (*45*). MeRIP combined with reverse transcription quantitative polymerase chain reaction (RT-qPCR) analysis (MeRIP-qPCR) confirmed the enrichment of m^6^A in mRNAs involved in ribosome biogenesis, revealing a significant down-regulation of m^6^A upon VIRMA depletion (Fig. 3C). In contrast, ribosomal protein L32 (RPL32) mRNA, which was an unmethylated control (fig. S7A), exhibited only an extremely low level of m^6^A enrichment (~0.1%) (Fig. 3C). This low level remained consistent in the forebrain of VIRMA cKO mice and is likely due to background noise from the immunoprecipitation (IP) process (Fig. 3C). Our threshold settings during the m^6^A peak calling process effectively filtered out this background binding noise. These results validate our MeRIP-m^6^A-seq dataset and highlight a significant reduction in m^6^A modification of these genes following VIRMA depletion (Fig. 3C and fig. S7A). We further confirmed the abnormal up-regulation of these ribosome biogenesis-related genes using RT-qPCR (Fig. 3D and fig. S7B). For the genes with available antibodies, we also performed WB and immunostaining to validate their abnormal up-regulation (Fig. 3E and fig. S7, C to F). In addition, we observed extended mRNA half-lives of these genes in VIRMA cKO NPCs using ActD-mediated transcription inhibition assay combined with RT-qPCR (Fig. 3F and fig. S8A), further supporting their dysregulation upon VIRMA depletion. In addition to these nine genes, RNA and m^6^A sequencing also identified other genes implicated in ribosome biogenesis, including but not limited to UTP14A, FBLL1, and DIEXF, which we did not comprehensively analyze.

YTHDF2 is a well-characterized m^6^A binding protein responsible for target mRNA degradation (*4*–*6*). In light of this, we hypothesized that YTHDF2 may bind to these m^6^A-modified genes involved in ribosome biogenesis. On the basis of a published YTHDF2-RIP-seq dataset (*46*), we found that among the genes with Hypo-m^6^A peaks upon VIRMA depletion (4923 genes; table S2), 43.22% of them (2128 genes) were bound by YTHDF2. YTHDF2-bound targets with VIRMA-mediated m^6^A modification (YTHDF2-RIP & Hypo-m^6^A) exhibited a tendency toward up-regulation and extended half-lives upon VIRMA depletion (fig. S8, B and C). Moreover, among the 287 up-regulated genes with Hypo-m^6^A peaks, 55.7% of them (160 genes) were YTHDF2 targets (fig. S8D). We validated these findings through YTHDF2-RIP-RT-qPCR experiments (Fig. 3G and fig. S8E), confirming that these ribosome biogenesis-related molecules exhibiting mRNA decay abnormalities upon VIRMA depletion are high-confidence YTHDF2 targets. Furthermore, using short hairpin RNA (shRNA)–mediated knockdown followed by RT-qPCR analysis, we demonstrated that YTHDF2 deficiency in NPCs resulted in the up-regulation of most ribosome biogenesis genes (fig. S8F), further supporting our conclusions. Notably, most of the VIRMA protein did not localize to the primary site of ribosome biogenesis, namely, the nucleoli marked by fibrillarin (FBL) or nucleophosmin (NPM1) (fig. S8G). This observation suggests that the effects of VIRMA depletion in NPCs are primarily mediated through m^6^A modification. In conclusion, these findings demonstrate that VIRMA deficiency and consequent m^6^A depletion lead to abnormal up-regulation of genes associated with ribosome biogenesis during brain development.

### VIRMA deficiency impairs ribosome biogenesis, triggers p53-dependent stress response, and compromises global protein translation

**Fig. 4.**
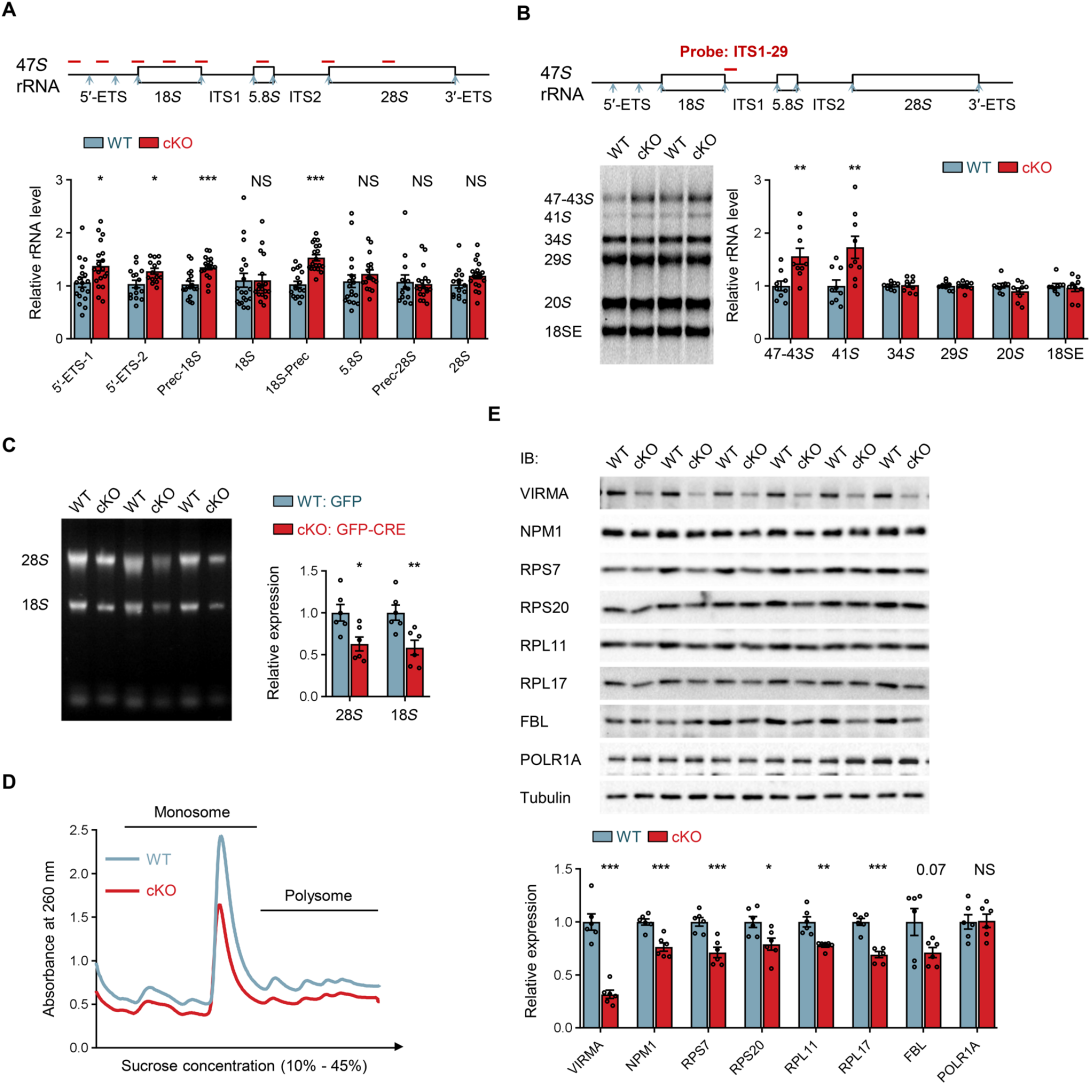
VIRMA deficiency leads to accumulation of rRNA precursors and impairs ribosome biogenesis. (**A**) RT-qPCR analysis to assess the expression levels of different rRNA species in the E13.5 forebrain of VIRMA WT and cKO mice. Equal amounts of total RNA (1 μg) were reverse transcribed into cDNA in each sample, which were subsequently used for RT-qPCR. The Ct values were normalized to GAPDH control, which were similar in both WT and cKO samples. In the 47*S* rRNA diagram above the graph, red short lines indicate the relative positions of qPCR products, and blue arrows denote cleavage sites in rRNA processing. *N* = 13 to 19 samples per genotype. (**B**) Northern blot analysis was performed using equal amounts of total RNA isolated from VIRMA WT and cKO forebrain. The 47*S* rRNA diagram is similar to that in (A), with the hybridization probe indicated as a red short line. 47-43*S* represents closely migrating 47*S*, 45*S*, and 43*S* pre-rRNAs. *N* = 9 samples per genotype. (**C**) Agarose gel electrophoresis of total RNA to analyze the expression levels of 28*S* and 18*S* rRNA in cultured NPCs. Total RNAs were extracted from an equal number of cultured WT and VIRMA cKO NPCs and then loaded onto an agarose gel. *N* = 6 samples per condition. (**D**) Polysome profiling from an equal number of cultured WT and VIRMA cKO NPCs demonstrating decreased monosome and polysome levels upon VIRMA depletion. (**E**) WB analysis of ribosome-associated proteins in lysates from the E13.5 forebrain of WT and VIRMA cKO mice. *N* = 6 samples per genotype. Unpaired Student’s *t* test in (A), (B), (C), and (E), *P < 0.05, **P < 0.01, and ***P < 0.001, compared with WT samples. Data are means ± SEM.

Given the role of VIRMA-mediated m^6^A modification in modulating gene expression associated with rRNA processing and ribosome biogenesis, we investigated how the up-regulation of these genes following VIRMA depletion affects rRNA and ribosome levels. Using RT-qPCR analysis targeting precursor and mature rRNA regions with equal amounts of total RNA, we detected elevated levels of rRNA precursors in the E13.5 forebrain of VIRMA cKO mice (Fig. 4A). This observation was corroborated by Northern blot assay (Fig. 4B). Moreover, we noted reduced 28*S* and 18*S* rRNA levels in an equal number of NPCs upon VIRMA depletion (Fig. 4C). Notably, we used a 5-ethynyl uridine (EU)–based labeling assay to exclude augmented RNA polymerase I transcription as the cause of elevated rRNA precursor levels (fig. S9). These results collectively suggest that VIRMA depletion predominantly affects posttranscriptional rRNA processing, rather than polymerase I transcription. Given the central role of rRNA in ribosome biogenesis, impaired rRNA processing could hinder mature ribosome production, potentially leading to reduced cytoplasmic ribosomes available for translation. This notion was supported by our observation of reduced content of monosomes and polysomes upon VIRMA depletion through quantitative polysome profiling in comparable numbers of NPCs (Fig. 4D). Furthermore, using WB and immunostaining analysis, we observed a reduction in the protein levels of NPM1 and essential ribosome component proteins, including RPL11, RPS7, RPS20, and RPL17, in both the forebrain of VIRMA cKO mice and VIRMA-depleted NPCs (Fig. 4E and fig. S10, A to D). However, RT-qPCR analysis did not reveal any changes in mRNA levels of NPM1 and these essential ribosome components following VIRMA depletion (fig. S10, E and F). Collectively, these results point toward an outcome of reduced ribosome content upon VIRMA depletion, underscoring the necessity of VIRMA for proper rRNA processing and ribosome biogenesis.

**Fig. 5.**
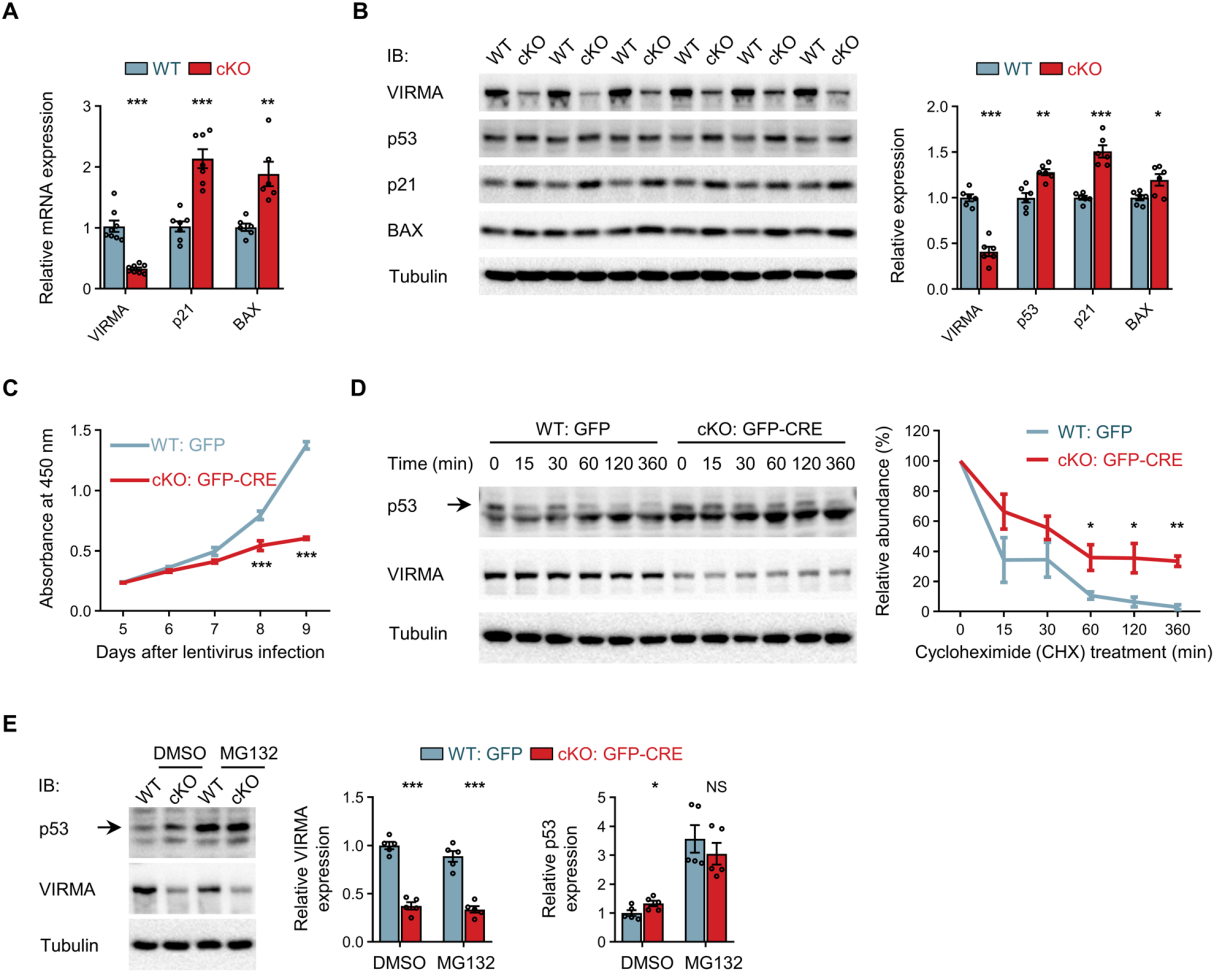
VIRMA deficiency triggers a p53-dependent stress response. (**A**) RT-qPCR analysis showing the enhanced transcription levels of p53 target genes, including p21 and BAX, in the E13.5 cKO forebrain. The Ct values were first normalized to the GAPDH controls, which were similar in both WT and cKO samples. *N* = 6 to 8 samples per genotype. (**B**) WB analysis showing the expression levels of tumor suppressor protein p53, p21, and BAX in the forebrain of E13.5 WT and VIRMA cKO mice. *N* = 6 samples per genotype. (**C**) Growth curves of cultured WT and VIRMA cKO NPCs. Cell numbers were determined by CCK-8 assay. The assays were repeated three times, and one representative result is shown. (**D**) Protein turnover analysis for p53 indicated enhanced stability of p53 upon VIRMA depletion. In cultured NPCs with or without VIRMA depletion, p53 protein levels at different time points (0, 15, 30, 60, 120, and 360 min) after CHX (50 μg/ml) treatment were normalized to tubulin abundance and presented as a percentage of p53 amount at 0 min. *N* = 3 biologically independent experiments. (**E**) Cultured NPCs with or without VIRMA depletion were treated with 20 μM MG132 or dimethyl sulfoxide (DMSO) solvent control for 6 hours and lysed for WB analysis. p53 protein levels were normalized to tubulin abundance. *N* = 5 samples per condition. Unpaired Student’s *t* test in (A) to (E), *P < 0.05, **P < 0.01, and ***P < 0.001, compared with WT samples. Data are means ± SEM.

The tumor suppressor p53, a pivotal transcription factor, coordinates signals from diverse stressors, such as ribosomal dysfunction, DNA damage, and oncogenic cues, to orchestrate essential antiproliferative or proapoptotic pathways vital for tissue homeostasis and cancer suppression (*47*–*50*). Under normal conditions, p53 is a highly unstable protein that remains at very low levels due to rapid ubiquitination by the E3 ubiquitin ligase MDM2, followed by degradation through the proteasome (*47*, *48*, *50*). However, in response to stress signals, p53 accumulates rapidly and becomes active as a transcription factor (*47*, *48*, *50*). Expanding on this, we proceeded to examine whether VIRMA depletion-induced ribosome biogenesis impairment initiates a p53-dependent stress response. Depletion of VIRMA in the forebrain increased the transcription levels of p53 target genes, including the cell cycle inhibitor p21 and the proapoptotic gene BAX (Fig. 5A), indicating activation of p53 as a transcription factor. The protein levels of p53, along with its downstream targets, p21 and BAX, were elevated following VIRMA depletion (Fig. 5B), suggesting that VIRMA depletion leads to the accumulation of p53 protein and the induction of antiproliferative or proapoptotic effects. This aligns with earlier findings of impaired NPCs proliferation and abnormal apoptosis in VIRMA cKO forebrain (Fig. 1, E to H). Moreover, we observed reduced proliferation of cultured VIRMA cKO NPCs, validated through the Cell Counting Kit-8 (CCK-8) assay, BrdU assay, and immunostaining of proliferating cell nuclear antigen (PCNA) (Fig. 5C, and fig. S11). The absence of m^6^A modification on p53 mRNA, as revealed by MeRIP-m^6^A-seq and qPCR analyses (fig. S12, A and B), suggests that its expression is not directly influenced by m^6^A-mediated modulation. Instead, we confirmed that the increased p53 protein levels in VIRMA-deficient NPCs result from protein stabilization rather than increased synthesis (Fig. 5, D and E). We conducted a chase experiment on cultured NPCs by treating them with the protein synthesis inhibitor, cycloheximide (CHX). Quantification of p53 protein levels revealed that VIRMA depletion slowed down the degradation of p53 (Fig. 5D). In addition, treatment with the proteasomal inhibitor MG132 confirmed that the delayed degradation of p53 was due to the inhibition of proteasome-mediated degradation (Fig. 5E). Although studies in cancer have reported that reduced MDM2 m^6^A levels due to METTL3 or METTL14 knockdown lead to decreased mRNA stability and p53 pathway activation (*51*, *52*), this does not appear to be the case in NPCs. Although VIRMA depletion in NPCs significantly decreased the m^6^A level on MDM2 mRNA (fig. S12, A and B), MDM2 mRNA stability did not decrease, and its mRNA level remained unchanged compared to control cells (fig. S12, C and D). Notably, previous studies have shown that, in the context of ribosomal dysfunction, several ribosomal proteins, particularly RPL5 and RPL11, bind to MDM2, inhibiting MDM2-mediated degradation of p53 and leading to p53-dependent antiproliferative or proapoptotic effects (*47*, *48*, *50*). Consistent with these findings, our IP and WB analyses showed that VIRMA depletion enhanced the interaction between MDM2 and ribosomal proteins RPL5 and RPL11 (fig. S12E). This finding supports the notion that the delayed degradation of p53 upon VIRMA depletion is linked to impaired ribosome biogenesis, which can activate p53 by inhibiting MDM2 through ribosomal proteins. Together, these findings demonstrate that VIRMA deficiency impairs ribosome biogenesis and triggers a p53-dependent stress response, leading to cell cycle arrest and apoptosis.

**Fig. 6.**
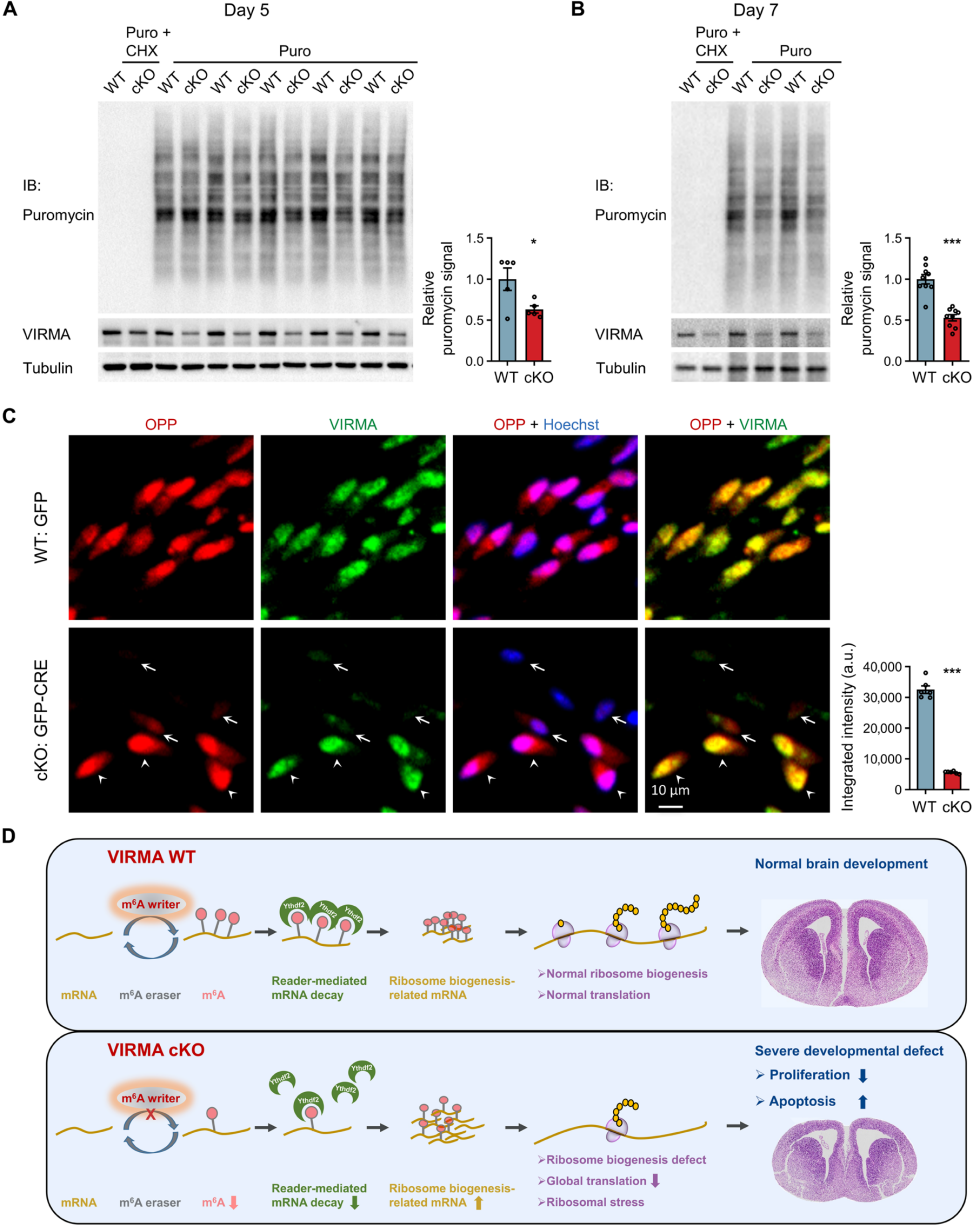
VIRMA deficiency impairs global protein translation. (**A** and **B**) SUnSET analysis of protein translation in cultured VIRMA WT and cKO NPCs treated with puromycin (Puro) or puromycin plus CHX (Puro + CHX) for 30 min. CHX was used for translation inhibition control. NPCs at 5 days (A) or 7 days (B) after lentivirus infection were used. *N* = 5 to 9 samples per condition. (**C**) Representative images of translation rate measured by OPP click chemistry in cultured WT and VIRMA cKO NPCs. The GFP fluorescence signal was lost due to the click reaction. Accordingly, VIRMA knockout efficiency was visualized by immunostaining with a VIRMA antibody. Noninfected WT NPCs and infected VIRMA cKO NPCs are denoted by arrowheads and arrows, respectively. The faint VIRMA signal in the VIRMA cKO (arrow) NPCs comes from nonspecific background staining. Nuclei were counterstained with Hoechst. Integrated intensity of the OPP signal in each cell was quantified based on *n* = 6 images. a.u., arbitrary units. (**D**) Schematic diagram illustrating the mechanisms underlying the regulatory functions of VIRMA-mediated m^6^A modification in ribosome biogenesis during embryonic forebrain formation. Unpaired Student’s *t* test in (A) to (C), *P < 0.05 and ***P < 0.001, compared with WT samples. Data are means ± SEM.

To investigate the impact of VIRMA depletion on translation subsequent to impaired ribosome biogenesis, we performed a surface sensing of translation (SUnSET) assay using puromycin pulse chasing to measure protein synthesis rate in cultured NPCs. Our results revealed substantial reduction in global translation due to VIRMA deficiency (Fig. 6, A and B). Additional quantification using *O*-propargyl-puromycin (OPP) click chemistry further confirmed compromised global protein translation in VIRMA-depleted NPCs (Fig. 6C and fig. S12F). Consistent with our previous observations that YTHDF2 deficiency up-regulated most ribosome biogenesis genes similarly to VIRMA depletion (fig. S8F), we demonstrated that YTHDF2 knockdown in NPCs also impaired ribosome biogenesis and reduced global protein translation (fig. S13A). In addition, YTHDF2 deficiency significantly impaired cell proliferation and enhanced apoptosis, as evidenced by decreased levels of the proliferation marker PCNA, increased levels of the apoptosis marker cleaved CASP3, and results from CCK-8 assays (fig. S13). These findings strongly corroborate our discovery that m^6^A modification controls the stability of transcripts involved in ribosome biogenesis and downstream protein synthesis during brain development.

Although the above observations primarily focused on NPCs, it is reasonable to extrapolate these findings to other rapidly proliferating cells, particularly cancer cells. This is due to the pivotal role of ribosome biogenesis in the growth and proliferation of various cell types, including cancer cells. Multiple studies have showcased elevated VIRMA levels in various cancers, including breast, colorectal, and non–small cell lung carcinoma (*22*, *23*). Enrichment analyses of the top 100 codependent genes identified via CRISPR (DepMap 22Q2 Public+Score, Chronos) revealed a noteworthy correlation between VIRMA and genes involved in ribosome biogenesis in cancer cells (fig. S14A). Therefore, it is worth exploring whether up-regulated VIRMA in cancer cells orchestrates ribosome biogenesis. To address this, we examined two cancer cell lines, MCF7 and HeLa, using shRNA knockdown to investigate the impact of VIRMA suppression on ribosome biogenesis. We found that endogenous VIRMA colocalized with SC35 in nuclear speckles of MCF7 and HeLa cells (fig. S14, B and C), and VIRMA knockdown impaired ribosome biogenesis and reduced global protein translation (fig. S14, D and E). Consistently, down-regulation of VIRMA in MCF7 and HeLa cells impaired cell proliferation and enhanced apoptosis. This was evidenced by a significant decrease in the levels of PCNA, an increase in the levels of cleaved CASP3, and results from the CCK-8 assay (fig. S14, D to G). In summary, our findings reveal the essential role of VIRMA-mediated m^6^A modification in adapting the protein synthesis machinery by controlling ribosome biogenesis in actively proliferating cells, in both brain development and cancer.

## DISCUSSION

Ribosome biogenesis is a complex and energy-intensive process orchestrated by hundreds of factors, including rRNA processing factors and ribosome assembly factors (*53*, *54*). Compromised ribosome biogenesis can lead to neurodevelopmental disorders, such as microcephaly (*55*, *56*), whereas enhanced ribosome biogenesis has been linked to uncontrolled cell growth and proliferation, as observed in cancer development (*54*, *57*, *58*). However, the molecular mechanisms governing ribosome biogenesis during development are not fully investigated. In this study, we elucidate an important epitranscriptomic mechanism underlying ribosome biogenesis in NPCs during embryonic forebrain development (Fig. 6D). Our study reveals that VIRMA-dependent m^6^A modification regulates ribosome biogenesis by modulating the mRNA decay of genes involved in this process (Fig. 6D). Specifically, VIRMA deficiency leads to an extended half-life of mRNA and aberrant up-regulation of key ribosome biogenesis factors (Fig. 3 and figs. S7 and S8), including NCL, a highly abundant nucleolar protein known to play pivotal roles in multiple steps of ribosome biogenesis (*37*–*39*). Previous studies have predominantly focused on investigating the functional roles of individual ribosome biogenesis factors using gene knockout or knockdown strategies. These studies have consistently demonstrated that the down-regulation of these factors results in a decrease in ribosome expression and overall protein synthesis. Conversely, up-regulation of ribosome biogenesis factors is often observed in actively proliferating cancer cells, where enhanced ribosome production supports rapid cell growth and division. Consequently, it has generally been assumed that the down-regulation of these factors leads to decreased ribosome expression, whereas their up-regulation promotes ribosome biogenesis. However, it is well established that maintaining balanced expression of certain genes is crucial for proper cellular function. Numerous studies have demonstrated that both overexpression and underexpression of specific genes can result in defects in associated biological processes. For example, both the loss of function and the increased dosage of SHANK3 or MECP2 can cause neurological disorders (*59*–*61*). These results indicate that excessive mRNA levels can also lead to abnormal cellular function, highlighting the importance of maintaining a balanced expression of these genes. In a similar vein, several studies have reported that high expression levels of certain ribosome biogenesis factors, such as NCL, RPP38, and SHQ1, can be detrimental to cell growth and survival (*62*–*64*). Notably, overexpression of SHQ1 has been shown to inhibit rRNA processing (*62*). These findings support our observation that the stabilization and overexpression of ribosome biogenesis factors due to VIRMA depletion-mediated m^6^A down-regulation may lead to defects in ribosome biogenesis. Our study offers a fresh perspective by identifying potential defects in ribosome biogenesis that arise from abnormal up-regulation of these genes due to m^6^A down-regulation associated with VIRMA depletion. This highlights the critical need for precisely regulated mechanism within cells to maintain balanced expression of genes essential for proper ribosome biogenesis during development. Given the huge energy demand required for ribosome biogenesis, we speculate that this finely tuned mechanism is designed to prevent excessive energy consumption and overproduction of ribosomes, thereby ensuring proper organismal homeostasis. As a reversible and rapid RNA-level epigenetic modification, m^6^A emerges as an effective regulatory mechanism for fine-tuning gene expression. This discovery adds an additional layer of complexity to the regulatory mechanisms governing ribosome biogenesis.

In our study, we reveal the crucial role of VIRMA in preserving the protein stability of the entire writer complex and demonstrate the impact of m^6^A down-regulation, resulting from VIRMA depletion, on active ribosome biogenesis. It is noteworthy that m^6^A regulation of ribosome biogenesis in brain development has not been previously reported in mice lacking METTL3 or METTL14, despite a substantial reduction in m^6^A levels and the presence of similar brain developmental defects in those mice (*8*–*11*). Distinct molecular mechanisms underlying m^6^A regulation in embryonic brain development are highlighted in different studies, even when using the same mouse model. For example, using the *Nestin*–*Cre*-mediated METTL14 cKO mouse model, one study reported that m^6^A depletion resulting from METTL14 knockout prolonged the cell cycle of NPCs and extended cortical neurogenesis into postnatal stages (*11*). On the other hand, another study using the same model demonstrated that m^6^A regulates NPCs self-renewal through its influence on histone modifications by destabilizing transcripts encoding H3K27 acetyltransferases (*10*). Using an *Emx1*–*Cre*-mediated VIRMA cKO mouse model, we found that the down-regulation of m^6^A due to VIRMA depletion resulted in impaired ribosome biogenesis. This impairment subsequently led to decreased proliferation and increased apoptosis of NPCs. Another study used a similar *Emx1*–*Cre*-mediated METTL3 cKO mouse model and performed some of the same analyses at the same developmental stage (E13.5) (*9*). The use of identical knockout strategy allows us to directly compare the effects of deleting VIRMA and METTL3. In the E13.5 METTL3 cKO forebrain, no significant changes in the number of BrdU-positive proliferative cells were observed, indicating that METTL3 deficiency does not affect NPC proliferation (*9*). The median survival time for METTL3 cKO mice was 29 days, which is longer than that of VIRMA cKO mice, and notably, 30% of the METTL3 cKO mice survived for at least 80 days (*9*). These results indicate that deletion of the structural component VIRMA has a more pronounced impact on brain development than deletion of the catalytic component METTL3, which was unexpected. The difference in mechanisms observed when knocking out VIRMA versus catalytic components METTL3 or METTL14 may be attributed to several factors. One possibility is that VIRMA contains three of the four RNA binding sites within the complex (*18*), enabling effective binding of the VIRMA-containing writer complex to specific RNA substrates, such as transcripts related to ribosome biogenesis. Furthermore, VIRMA may cooperate with other METTL3/METTL14-like proteins to facilitate m^6^A deposition on ribosome biogenesis-related transcripts, maintaining functionality even in the absence of the catalytic components METTL3 or METTL14. Future experiments aimed at identifying additional partners of VIRMA within the writer complex are needed and will enhance our understanding of m^6^A regulation during brain development.

Recent studies have sporadically reported the involvement of m^6^A modification in ribosome biogenesis, with a predominant focus on m^6^A modification of rRNA (*65*–*70*). Notably, two highly conserved m^6^A sites have been identified on eukaryotic 18*S* and 28*S* rRNA, catalyzed by METTL5 and ZCCHC4, respectively (*66*, *67*). This is in contrast to m^6^A modifications on mRNA, which are primarily deposited by the methyltransferase complex consisting of METTL3/METTL14 and VIRMA (*71*). Consistently, our results demonstrate that VIRMA deficiency does not affect the m^6^A levels observed in 18*S* and 28*S* rRNA (Fig. 2C). However, VIRMA deficiency leads to a pronounced decrease in m^6^A levels specifically on mRNA (Fig. 2C). These findings strongly support the notion that m^6^A events on mRNA and rRNA are governed by distinct enzyme systems. The mechanisms through which these enzyme systems achieve such strong substrate specificities are intriguing questions warranting further investigation in future studies. In addition, a recent study has indentified METTL3 as a key regulator of ribosome levels and translation through distinct mechanisms in chronic myeloid leukemia (*65*). It has been revealed that METTL3 indirectly modulates the levels of myelocytomatosis oncogene (MYC), a transcriptional activator of genes involved in ribosome biogenesis (*65*). Moreover, cytoplasmic METTL3 has been found to directly regulate the translation of Pescadillo (PES1), a crucial regulator of ribosome biogenesis and cell proliferation, through a catalytic activity–independent mechanism (*65*). These observations contrast with the VIRMA-mediated pathway, as supported by our omics data, which showed minimal changes in MYC and PES1 expression. Together, these findings emphasize the multifaceted nature of ribosome biogenesis regulation and underscore the diverse functions of METTL3.

The VIRMA-WTAP-CBLL1-ZC3H13 complex exhibits remarkable conservation across metazoans (*20*), indicating its potential involvement in coordinating diverse growth processes within multicellular organisms. During the development and maturation of multicellular organisms, the transition from a single cell to multicellular structure often relies on various types of stem and progenitor cells with robust proliferation and differentiation capacities (*72*). Ribosome biogenesis actively participates in the self-renewal and differentiation of stem and progenitor cells. Our findings are consistent with the potential role of VIRMA in multicellularity, and we hypothesize that VIRMA-mediated m^6^A modification has evolved to fine-tune ribosome function and introduce translational plasticity in multicellular organisms in response to metabolic requirements. This insight may broaden our understanding of ribosome assembly and regulation beyond that observed in bacteria and yeast. We observed a discrepancy between mRNA levels and protein expression of ZC3H13 and CBLL1 upon VIRMA depletion. Our MeRIP-m^6^A-seq data indicate that m^6^A is enriched in ZC3H13 and CBLL1 mRNAs (table S2), and these transcripts are also identified as YTHDF2-bound targets based on a published YTHDF2-RIP-seq dataset (*46*). This suggests that VIRMA-dependent m^6^A modification may regulate the decay of ZC3H13 and CBLL1 mRNAs through YTHDF2, potentially leading to increased mRNA levels upon VIRMA depletion. The observed increase in mRNA levels coupled with reduced protein expression for ZC3H13 and CBLL1 suggests two possible scenarios: First, defects in ribosome biogenesis resulting from VIRMA depletion may lead to a decrease in functional ribosomes and overall protein synthesis; second, even if the mRNAs are translated, the proteins might be rapidly degraded, leading to lower steady-state protein levels. VIRMA depletion could destabilize the entire writer complex, possibly affecting the stability of multiple protein components within it. Further investigations will help to clarify these mechanisms.

Recent studies have uncovered complex interactions between m^6^A modification and p53 signaling in specific cancers (*51*). For instance, YTHDF2 promotes the degradation of m^6^A-modified p53 mRNA, destabilizing p53 in ocular melanoma (*73*). In addition, METTL3 has been identified as a previously unidentified p53-interacting protein that stabilizes p53 by competing with MDM2 (*49*), whereas the methyltransferase complex also influences p53 signaling by modulating the mRNA stability of p53 ubiquitin ligases MDM2 and FBXO43 (*52*, *74*), which are crucial for p53 degradation. However, our data suggest that these m^6^A-p53 pathway interactions may not be conserved in NPCs. First, MeRIP-m^6^A-seq and qPCR analyses (fig. S12, A and B) indicate that p53 mRNA lacks m^6^A modification in NPCs, suggesting that p53 expression is not directly influenced by m^6^A modulation. Second, despite reduced METTL3 levels in VIRMA cKO mice (Fig. 2B), this cannot explain the elevated p53 levels as lower METTL3 should decrease p53 stabilization by reducing competition with MDM2. Third, although VIRMA depletion decreases the m^6^A level on MDM2 mRNA (fig. S12, A and B), MDM2 mRNA stability did not decrease, and its mRNA levels remained unchanged (fig. S12, C and D). In addition, RNA-seq data show extremely low FBXO43 expression in NPCs (table S3), suggesting that FBXO43 is unlikely to regulate p53. Instead, our findings indicate that ribosome biogenesis disruptions upon VIRMA depletion activate the impaired ribosome biogenesis checkpoint, leading to p53 activation by inhibiting MDM2 through ribosomal proteins. This signaling pathway involving ribosomal proteins, MDM2, and p53 acts as a molecular switch, potentially serving as a surveillance system to monitor ribosome biogenesis integrity.

VIRMA has been primarily investigated in the context of cancer and has been reported as an oncogenic factor in various cancer types, including breast cancer (*22*, *23*). However, the specific molecular mechanisms underlying its oncogenic role vary notably, involving both m^6^A-dependent and m^6^A-independent pathways. In addition to its role in neural stem cells, robust ribosome biogenesis is also crucial for the active proliferation of cancer cells. Although our initial conclusion regarding the involvement of VIRMA-mediated m^6^A modification in regulating ribosome biogenesis was drawn from NPCs, we were able to replicate similar ribosome biogenesis defects in some VIRMA-depleted cancer cells, including MCF7 and HeLa cells (fig. S14). These results demonstrate that the observed effects may be common in constantly dividing cells. Moreover, the essential role of VIRMA in cell survival has been demonstrated in several cell lines, including HeLa cells (*13*, *75*, *76*), potentially through its regulation of ribosome biogenesis, which is vital for cellular functionality. However, the complex mechanisms and heterogeneity observed in different cancers, including the m^6^A-p53 pathways mentioned above, limit the generalizability of our findings across all cancer types.

In summary, our findings establish the first link between mRNA m^6^A modification and active ribosome biogenesis, shedding light on a previously unexplored regulatory mechanism. The VIRMA-mediated m^6^A pathway in ribosome biogenesis reveals an additional layer of control exerted by mRNA m^6^A on translation. This discovery broadens our understanding of the intricate regulatory networks governing protein synthesis and highlights the importance of mRNA modification in fine-tuning cellular processes.

## MATERIALS AND METHODS

### VIRMA cKO mice

The *Virma^flox/flox^* mice with a C57BL/6J genomic background, previously described (*29*), were generated in B.S.’s Laboratory. Emx1-Cre mice (the Jackson Laboratory, stock 005628, generously provided by L. Cheng at Southern University of Science and Technology, Shenzhen, China) were bred and maintained on a C57BL/6J genomic background. *Virma^flox/flox^* mice were first crossed with Emx1-Cre mice to generate *Emx1*–*Cre;Virma^flox/+^* mice. *Emx1*–*Cre;Virma^flox/+^* and *Virma^flox/flox^* breeding pairs produced offspring with *Emx1*–*Cre;Virma^flox/flox^*, *Emx1*–*Cre;Virma^flox/+^*, *Virma^flox/flox^*, and *Virma^flox/+^* genotypes in a Mendelian ratio of 1:1:1:1, indicating that embryonic ablation of VIRMA is not lethal. Mice carrying the *Emx1*–*Cre;Virma^flox/flox^* genotype were referred to as VIRMA cKO mice, which exhibited specific deletion of the conditional VIRMA alleles in the developing forebrain, including both the cortical and hippocampal regions (*25*). Littermates carrying the *Virma^flox/flox^* or *Virma^flox/+^* genotype were used as WT controls (VIRMA WT) for comparison.

The genotyping of the floxed *Virma* gene was performed using PCR with the following primer pair: forward primer (F1) 5′-TG-CCACATAGATGCGATGCC-3′ and reverse primer (R1) 5′-CA-AGACCCTGGGTTGAACCC-3′. For *Emx1*–*Cre*, the forward primer used was 5′-GTCCAATTTACTGACCGTACACC-3′, and the reverse primer was 5′-GTTATTCGGATCATCAGCTACACC-3′. For the mutated allele (*Virma* cKO) after Cre/lox-mediated genetic recombination, the forward primer (F2) used was 5′-TTGCCTAC-TTCATTTCTATTCAT-3′, and the reverse primer (R2) was 5′-AGC-TCAGACCATAACTTCGT-3′. The specific PCR products for the *Virma* WT and floxed alleles were 254 and 438 base pairs (bp), respectively. The specific PCR product for the *Emx1*–*Cre* allele was 706 bp, and that for the mutated allele (*Virma* cKO) was 628 bp.

The exact gestational age of pregnant female mice was determined by examining the presence of the vaginal plug resulting from mating. Animals of both sexes were included in the study, with the identification of the sex of mouse embryos posing challenges. All mice were housed in a specific pathogen–free animal facility and maintained under a 12-hour/12-hour light/dark cycle with free access to food and water. All experimental procedures involving animals were conducted in compliance with the protocols (SIAT-IACUC-20200226-NS-NENJSYS-WM-A1017-01 and SIAT-IACUC-210531-NS-ZT-A1930) approved by the Animal Research Committee at the Shenzhen Institute of Advanced Technology, Chinese Academy of Sciences (SIAT).

### Primary culture of NPCs

Primary NPCs were prepared from *Virma^flox/flox^* mouse embryos at E13.5, a developmental stage known for its abundance of neural stem cells. The forebrain was dissected and subjected to in vitro NPC culture using the NeuroCult Proliferation Kit (STEMCELL Technologies, 05702) according to the manufacturer’s instructions. To promote NPC expansion, the culture medium was supplemented with human recombinant epidermal growth factor (20 ng/ml; STEMCELL Technologies, 78006.1). The NPCs were seeded as a monolayer onto dishes or coverslips, which had been precoated sequentially with poly-l-ornithine (Sigma-Aldrich, P4638) and Laminin (Sigma-Aldrich, L2020). The cells were maintained at 37°C with 5% CO_2_ in a humidified incubator. When the NPCs reached 80% confluence, they were passaged using ACCUTASE cell detachment solution (STEMCELL Technologies, 07920). The subculture procedure was performed through multiple passages, not exceeding 13 passages. After 24 hours of passage, the NPCs were infected with lentivirus carrying either GFP-CRE or GFP control. The medium was replaced with fresh medium 24 hours after infection. Experimental analyses were conducted 5 to 7 days after infection, with two to three rounds of passages in between. To confirm the NPCs’ identity, immunocytochemistry (ICC) was performed using an antibody against the neural stem cell marker SOX2 (Millipore, AB5603, 1000 for ICC). The results demonstrated that over 95% of the cultured cells used for experiment were positive for SOX2, validating their NPC identity.

### Culture of cell lines

The human breast cancer cell line MCF7 and cervical cancer cell line HeLa (gifts from H. Wang, SIAT, originally from the American Type Culture Collection) were cultured in Dulbecco’s modified Eagle’s medium (Gibco, C11995500BT) supplemented with 10% fetal bovine serum (Lonsera, S711-001S) and 1x Pen/Strep (Gibco, 15140-122). The cells were maintained in a humidified atmosphere with 5% CO_2_ at 37°C. For transfection, the AAV-shRNA plasmid was introduced into human MCF7 and HeLa cells using Lipo6000 (Beyotime, C0526) when the cell confluence reached 70 to 80%. The medium was completely changed 4 to 6 hours after transfection, and the cells were cultured for an additional 48 to 96 hours before harvesting for experiments. Both cell lines were confirmed to be negative for mycoplasma contamination before use.

### Antibodies

The following antibodies were used for WB, ICC, and immunohistochemistry (IHC) with the indicated dilutions: mouse monoclonal antibody to α-tubulin (Proteintech, 66031-1-Ig, 1:20,000 for WB), glyceraldehyde-3-phosphate dehydrogenase (GAPDH) (Thermo Fisher Scientific, AM4300, 1:60,000 for WB), β-actin (Sigma-Aldrich, A5441, 1:60,000 for WB), FBL (Abcam, ab4566, 1:6000 for WB, 1:200 for ICC), NPM1 (Sigma-Aldrich, B0556, 1:100,000 for WB, 1:20,000 for ICC), PCNA (Abcam, ab29, 1:4000 for WB, 1:100 for ICC), RPS7 (Santa Cruz, sc-377317, 1:8000 for WB), RPL17 (PTMab, PTM-5621, 1:2000 for WB), POLR1A/RPA194 (Santa Cruz, sc-48385, 1:500 for WB), puromycin (Millipore, MABE343, 1:2000 for WB), SC35 (Abcam, ab11826, 1:400 for ICC), PAX6 (BD Biosciences, 561462, 1:750 for IHC), GFAP (Sigma-Aldrich, G3893, 1:600 for IHC), MDM2 (Santa Cruz, sc-377317, 1:1000 for WB), and TUJ1 (Abcam, ab78078, 1000 for ICC); rabbit monoclonal antibody to VIRMA Ab#1 (Cell Signaling Technology, 88358S, 1:1000 for WB, 1:100 for ICC and IHC), METTL3 (Abcam, ab195352, 1:2000 for WB, 1:400 for ICC), cleaved GSDMD (Abcam, ab215203, 1:1000 for WB), RPS20 (PTMab, PTM-5834, 1:2000 for WB), and SATB2 (Abcam, ab92446, 1:750 for IHC); rabbit polyclonal antibody to β-tubulin (Proteintech, 10094-1-AP, 1:3000 for WB), YTHDF2 (Proteintech, 24744-1-AP, 1:2000 for WB), VIRMA Ab#2 (Proteintech, 25712-1-AP, RRID:AB_2880204, 1:2000 for WB), METTL14 (ABclonal, A8530, 1:2000 for WB), METTL14 (Sigma-Aldrich, HPA038002, 1:400 for ICC), WTAP (Proteintech, 10200-1-AP, 1:2000 for WB, 1:800 for ICC), ZC3H13 (Bethyl, A300-748A, 1:4000 for WB, 1:200 for ICC), CBLL1 (Bethyl, A302-969A, 1:4000 for WB, 1:400 for ICC), NCL (Abcam, ab70493, 1:4000 for WB, 1:7500 for IHC), RPL11 (Proteintech, 16277-1-AP, 1:3000 for WB, 1:500 for IHC), RPL5 (Proteintech, 29092-1-AP, 1:3000 for WB), PROX1 (Proteintech, 11067-2-AP, 1:200 for IHC), RPP38 (Sigma-Aldrich, HPA050398, 1:1000 for WB), POP7 (Proteintech, 14964-1-AP, 1:500 for WB, 1:50 for IHC), SHQ1 (Proteintech, 27020-1-AP, 1:2000 for WB, 1:100 for IHC), p53 (Proteintech, 10442-1-AP, 1:2000 for WB), p21 (Proteintech, 28248-1-AP, 1:1000 for WB), BAX (Proteintech, 50599-2-Ig, 1:2000 for WB), cleaved CASP3 (Cell Signaling Technology, 9661, 1:1000 for WB, 1:400 for IHC), and SOX2 (Millipore, AB5603, 1000 for ICC); and rat monoclonal antibody to CTIP2 (Abcam, ab18465, 1:750 for IHC) and BrdU (Abcam, ab6326, RRID:AB_305426, 1:1000 for ICC and IHC).

### Plasmid constructs and lentivirus

DNA sequences of shVIRMA (5′-GAGGATGATCGACGAACAG-TA-3′) for silencing human VIRMA expression and shCON control (5′- GCAAGCTGACCCTGAAGTTCAT-3′) were subcloned into the AAV-shRNA vector using the BbsI site (New England Biolabs, R3539L). The AAV-shRNA vector (AAV: ITR-CMV-Cerulean-SV40-shRNA-U6-ITR), as described in a previous study (*77*), was generously provided by J. Xia from Hong Kong University of Science and Technology (Guangzhou). The shCON used here was shGFP as in Addgene plasmid no. 30323. All constructs were confirmed by Beijing Genomics Institute (BGI) sequencing. Lentiviruses expressing shVIRMA, shCON, or shYTHDF2 (5′-GGACGTTCCCAATA-GCCAACT-3′) were prepared by Taitool Biotech (Shanghai) using the pLentai-hU6-shRNA-esEF1A-MataGFP-WPRE-pA vector. Lentiviruses expressing GFP-CRE (L7025) or GFP control (L7014) were ordered from Taitool Biotech (Shanghai).

### Western blot

Tissues or cultured cells were lysed in radioimmunoprecipitation assay (RIPA) buffer [50 mM tris-HCl (pH 7.4), 1% Triton X-100, 0.5% sodium deoxycholate, 0.1% SDS, 2 mM EDTA, and 150 mM NaCl] supplemented with a protease inhibitor cocktail (MCE, HY-K0010). The lysates were incubated at 4°C for 2 to 3 hours and then cleared by centrifugation at 18,000*g* at 4°C for 20 min. The protein concentration was determined using the Pierce BCA Protein Assay Kit (Thermo Fisher Scientific, 23227). Subsequently, samples were boiled at 90° to 100°C for 5 min with 3X SDS loading buffer and stored at −80°C until further use. The proteins in each sample were resolved on SDS–polyacrylamide gel electrophoresis gels (10 to 12.5%) and then transferred onto the Immobilon-NC Transfer Membrane (Merck, HATF00010). After transfer, the membrane was blocked in 0.5% nonfat milk solution for 60 min at room temperature (RT) and then incubated with diluted primary antibody solution at 4°C overnight, followed by incubation with diluted horseradish peroxidase–conjugated secondary antibody solution (Cell Signaling Technology, 7076s and 7074s, 1:2000 for WB) for 2 hours at RT. After washing, proteins were visualized using an enhanced chemiluminescence (ECL) substrate kit (Epizyme, SQ101L/SQ202L/SQ201L) and the ChemiDoc Touch Imaging System (Bio-Rad). The protein levels were determined using the gray values of protein bands, as measured using ImageJ software. Tubulin, actin, or GAPDH on the same membrane was used as the internal loading control. Uncut full WBs with appropriate ladders can be found in fig. S15. All antibodies used for WB analysis were tested on WB strips to ensure their specificity and reliability before use (fig. S16).

### Immunostaining

For in vivo experiments, mice at E13.5/E15.5/E17.5/P0/P5 were anesthetized on ice or with diethyl ether and decapitated. The mouse brains were rapidly removed and fixed in 4% paraformaldehyde (PFA) at 4°C for 24 hours. Mice at P10/P20 were anesthetized with sodium pentobarbital (50 mg/kg of body weight, intraperitoneal injection) and perfused with phosphate-buffered saline (PBS) followed by 4% PFA. The mouse brains were then dissected and postfixed in 4% PFA overnight at 4°C. Subsequently, the brains were dehydrated using 10, 20, and 30% sucrose solutions, and 10- or 35-μm-thick cryosections were cut using the Leica CM1950 Cryostat and stored at −80°C for IHC. For in vitro experiments, cultured cells seeded on coverslips were fixed with 4% PFA for 20 min. Both before and after fixation, the cells were washed with PBS. Subsequently, ICC was performed.

For both IHC and ICC, brain slices or cultured cells were subjected to permeabilization and blocking for 1.5 hours in PBST (PBS with 0.3% Triton X-100) containing 5% goat serum (Beyotime, C0265) at RT. Subsequently, the samples were incubated with primary antibodies at 4°C overnight, followed by detection with fluorescent secondary antibodies and counterstaining with Hoechst for 2 hours at RT. Fluorescent images were captured using the Olympus IX73 inverted microscope, Olympus VS120 Virtual Slide microscope, or Zeiss LSM 900/980 confocal microscope. Regions or cells were randomly selected for imaging. Comparable images were captured with identical settings and analyzed using ImageJ software.

### BrdU labeling

The proliferation capacity of NPCs was assessed using BrdU, an analog of thymidine that labels proliferating cells by incorporating into synthesizing DNA during the S phase of the cell cycle. For in vivo embryonic labeling, pregnant mice at gestation day 13.5 were intraperitoneally injected with BrdU (Sigma-Aldrich, B5002) at a dose of 100 mg/kg of body weight. After 15 min, the brains of the embryos were immediately collected, followed by fixation, dehydration, and sectioning for subsequent IHC. For in vitro labeling, NPCs seeded on coverslips at 7 days after lentivirus infection were incubated with 10 μM BrdU in complete culture medium for 60 min. Subsequently, the cells were washed twice with PBS and fixed with 4% PFA for 15 min for subsequent ICC.

Antigen retrieval was performed by immersing the sections in citrate buffer (Sangon Biotech, E673000) at 90° to 100°C for 15 min, followed by incubation at RT for 60 min. Subsequently, the sections were washed twice with PBS and treated with 2 M HCl for 30 min, followed by another three washes with PBS. The samples were then subjected to the standard immunostaining procedure, including blocking and antibody incubation. An anti-BrdU antibody (Abcam, ab6326, 1:1000 for ICC and IHC) was used to detect the incorporated BrdU. The GFP fluorescence signal was lost due to antigen retrieval.

### TUNEL assay

The TUNEL assay was performed using the DeadEnd Fluorometric TUNEL System (Promega, G3250) according to the manufacturer’s instructions. Brain cryosections were postfixed with 4% PFA for 20 min and then washed with PBS for 5 min. Next, the sections were permeabilized with PBST for 20 min and washed with PBS for 5 min. Subsequently, the samples were equilibrated with equilibration buffer for 10 min and then labeled with the Recombinant Terminal deoxynucleotidyl transferase (rTdT) reaction mixture for 2 hours at RT. The reaction was terminated by immersing the slides in 2X SSC for 15 min, and the samples were washed three times with PBS. The sections were counterstained with Hoechst, and fluorescence was detected using the Olympus VS120 Virtual Slide microscope. The images were analyzed using ImageJ software.

### EU labeling

To detect newly synthesized RNA, predominantly rRNA, we performed EU labeling using the Click-iT RNA Alexa Fluor 594 Imaging Kit (Invitrogen, C10330) following the manufacturer’s instructions. NPCs seeded on coverslips at 7 days after lentivirus infection were treated with 1 mM EU in the culture medium. After incubating for 60 min, cells were rinsed twice with PBS and fixed with 3.7% formaldehyde for 15 min at RT. Following a single wash with PBS, the cells were permeabilized with PBS containing 0.5% Triton X-100 for 15 min. Subsequently, the cells were incubated with the Click-iT reaction cocktail for 30 min at RT in the dark. After incubation, the samples were washed with Click-iT reaction rinse buffer and then with PBS. Next, a standard immunostaining procedure, including blocking and antibody incubation, was performed to label VIRMA and counterstain with Hoechst. The GFP fluorescence signal was lost due to the click reaction. To ensure labeling specificity, we included a negative control group in which ActD (1 μg/ml; Sigma-Aldrich, SBR00013), an RNA transcription inhibitor, was added 6 hours before EU treatment. The negative control group exhibited only a background signal. Fluorescent images were captured using the Zeiss LSM 900 confocal microscope, and the integrated intensity of the EU signal in each cell was quantified using Zen software (V.2.3; Carl Zeiss Inc.) based on six to seven images (with >150 cells). Consistent results were obtained from two independent experiments.

### H&E staining

E15.5 mouse brains were fixed in 4% PFA at 4°C and dehydrated through a series of graded alcohols. The brains were then embedded in paraffin wax, and 5-μm-thick paraffin sections were prepared. The sections were deparaffinized by immersion in xylene and rehydrated with graded alcohols. Subsequently, the tissues were sequentially stained with H&E solution. After staining, the tissues were dehydrated again with graded alcohols. Last, the tissues were immersed in xylene twice and mounted with neutral gum. Bright-field images were captured using an Olympus VS120 Virtual Slide microscope.

### Protein synthesis assay

To evaluate the global protein synthesis rate in cultured NPCs, we used two puromycin pulse chasing methods: the SUnSET assay and the OPP click chemistry followed by imaging. Puromycin, an aminoacyl-tRNA analog, incorporates into newly synthesized peptides, allowing us to monitor protein synthesis.

For the SUnSET assay, NPCs at 5 to 7 days after lentivirus infection were exposed to 1 μM puromycin (MCE, HY-B1743A) in culture medium for 30 min. After treatment, proteins were extracted from the cells using RIPA buffer, and the quantity of puromycin-labeled peptides was evaluated by WB using an anti-puromycin antibody (Millipore, MABE343, 1:2000 for WB).

For the OPP click chemistry followed by imaging, NPCs seeded on coverslips at 7 days after lentivirus infection were incubated with 25 μM OPP (Click Chemistry Tools, 1407-5) in culture medium for 60 min. Subsequently, the cells were washed twice with PBS before being fixed with 4% PFA for 15 min. After fixation, the cells were permeabilized with PBST for 15 min, followed by two additional washes with PBS. Next, the cells were incubated with a freshly prepared azide-alkyne cycloaddition reaction mix [308 μl of PBST, 7 μl of 100 mM CuSO_4_, 1 μl of biotin azide (Click Chemistry Tools, 1265-5), and 35 μl of ascorbic acid (20 mg/ml; Sigma-Aldrich, A92902)] at RT for 30 min in the dark. The cells were then washed twice with PBST and blocked with 5% goat serum for 1.5 hours before being incubated with an anti-VIRMA antibody (Cell Signaling Technology, 88358S, 1:100 for ICC) at 4°C overnight. Following this, the cells were washed three times with PBST and incubated in a solution containing Cy3-streptavidin (Jackson ImmunoResearch Laboratories, 016-160-084), Alexa Fluor 647–conjugated secondary antibody, and Hoechst for 2 hours at RT. The cells were washed three times with PBST and mounted before imaging using an Olympus IX73 inverted microscope. The images were analyzed using ImageJ software. A specific threshold of signal intensity was applied to the images, and the number of cells with an OPP signal above the threshold was manually counted. This count was then divided by the count of Hoechst-stained cells to obtain the ratio (OPP/Hoechst count). The GFP fluorescence signal was lost due to the click reaction.

To ensure assay specificity, we included a negative control group in which CHX (50 μg/ml; MCE, HY-12320), a protein synthesis inhibitor, was added 15 min before puromycin or OPP treatment. The negative control group exhibited only a background signal, confirming the specificity of the assay.

### Polysome profiling

At 7 days after lentivirus infection, cultured NPCs were treated with CHX (100 μg/ml) for 15 min at 37°C and then detached using ACCUTASE Cell detachment solution containing CHX (100 μg/ml). The cells were washed three times with ice-cold PBS containing CHX (100 μg/ml) and counted using a LUNA-II Automated Cell Counter. After counting, an equal number of cells (26 million) were pelleted and snap-frozen in liquid nitrogen before being sent to GuangZhou QingZe BioTech Co. Ltd. for further processing. The cell lysates were prepared using the polysome profiling cell lysis buffer and incubated on ice for 30 min. Subsequently, the lysates were centrifuged at 4°C at 13,000*g* for 10 min, and the supernatants were collected for further analysis. The supernatants were separated on 10 to 45% sucrose gradients by centrifuging at 36,000 rpm for 3 hours in a Beckman Optima XE-100 ultracentrifuge using an SW41 rotor. Following centrifugation, the gradients were fractionated using a Biocomp Gradient Station fractionator, and fractions were quantified by measuring absorbance at 260 nm. The distribution peaks of subunits, monosomes, and polysomes were determined based on the curve, and their relative abundances were compared to assess the protein translation status.

### Reverse transcription quantitative polymerase chain reaction

Total RNA was extracted from tissues or cultured NPCs using TRIzol reagent (Invitrogen, 15596026) following the manufacturer’s instructions. Subsequently, 1 μg of total RNA was reverse transcribed into cDNA using the SuperScript III First-Strand Synthesis System (Invitrogen, 18080051) according to the manufacturer’s protocols. The resulting cDNA was subjected to quantitative PCR using the AceQ Universal SYBR Green qPCR Master Mix (Vazyme, Q511-02) in the QuantStudio 3 Real-Time PCR System (Applied Biosystems). Each assay was performed in technical duplicate. The Ct values were first normalized to either GAPDH or RPL30 controls, which were similar in both WT and cKO samples. The relative expression level of the target mRNA or rRNA was calculated using the 2^−ΔΔCt^ method, enabling a comparison of the expression levels between different samples. The primers used for RT-qPCR are listed in table S5.

### RNA stability assay: Half-life RNA-seq analysis

At 7 days after lentivirus infection, cultured NPCs were treated with ActD (5 μg/ml; Sigma-Aldrich, SBR00013) to halt new RNA synthesis. At 0, 3, and 6 hours posttreatment, cells were washed once with PBS and collected with TRIzol reagent for total RNA extraction. The concentration and purity of total RNA were assessed through agarose gel electrophoresis and the Agilent 2100 Bioanalyzer system to ensure high-quality RNA. Subsequently, the RNA samples were sent to BGI for RNA-seq on the DNBSEQ platform, following their established protocols for library preparation available on their website (https://yuque.com/yangyulan-ayaeq/oupzan/gu8ls7). Detailed sequencing data analysis methods can also be found on the BGI website (https://yuque.com/yangyulan-ayaeq/oupzan). In brief, the sequencing data were filtered using SOAPnuke (v1.5.6), and subsequent analysis and data mining were performed using the Dr. Tom multi-omics data mining system (https://biosys.bgi.com). Bowtie2 (v2.3.4.3) was used to align the clean reads to the gene set, and the expression levels of genes were quantified in TPM (transcripts per million) using RSEM (v1.3.1). The RNA half-life was determined using the equation described previously (*78*). Four replicates were conducted for each genotype at each time point. In addition, RT-qPCR was performed to independently assess the RNA half-life of specific mRNA, serving as a validation step for the RNA-seq analysis.

### Northern blot

Northern blot was performed using the NorthernMax Kit (Invitrogen, AM1940) and the Chemiluminescent Nucleic Acid Detection Module (Thermo Fisher Scientific, 89880), following the manufacturer’s instructions. We optimized specific experimental parameters as described below. A total of 0.5 or 1 μg of total RNA was loaded into each well of the agarose gel and subsequently transferred to a Plus Positively Charged Nylon Membrane (Invitrogen, AM10100) using the downward transfer apparatus for 2.5 hours. The membrane was then baked at 80°C in a conventional oven for 30 min to cross-link the RNA. For hybridization to distinguish different rRNA precursors, single-stranded DNA probes labeled with biotin at both ends were synthesized by Sangon Biotech (Shanghai). The DNA probe sequence (ITS1-29: 5′-ACGCCGCCGCTCCTCCACAGTCTCCCGTT-3′) was designed based on a reference (*79*). The RNA bands were visualized using the Omni-ECL Femto Light Chemiluminescence Kit (Epizyme, SQ201L) and the ChemiDoc Touch Imaging System (Bio-Rad). The levels of different rRNA precursors were determined by measuring the gray values of the RNA bands using ImageJ software.

### CCK-8 assay

To generate cell growth curves, NPCs at 4 days after lentivirus infection were plated into 96-well plates at a density of 7000 cells per well, and MCF7 and HeLa cells at 1 day after transfection or lentivirus infection were plated into 96-well plates at a density of 1250 cells per well. Subsequently, cell numbers were assessed daily for five consecutive days using a CCK-8 (DOJINDO, CK04) according to the manufacturer’s instructions.

### LC-MS/MS quantification of m^6^A

The quantification of m^6^A was performed using LC-MS/MS following established protocols (*78*). Briefly, 100 ng of either poly(A)+ mRNA, total RNA, or rRNA was subjected to nuclease P1 digestion (1 U) in 17 μl of buffer containing 10 mM NH_4_Ac at 42°C for 2 hours. Subsequently, the reaction mixture was further incubated at 37°C for an additional 2 hours upon the addition of CutSmart Buffer (2 μl) and alkaline phosphatase (0.5 U). Next, 5 μl of the resulting solution was injected into the LC-MS/MS system. The nucleosides were separated by reversed-phase ultraperformance LC on a C18 column with online mass spectrometry detection using the Agilent 6410 QQQ triple-quadrupole LC mass spectrometer in positive electrospray ionization mode. The nucleosides were quantified by comparison with a standard curve generated from pure nucleoside standards run alongside the samples in the same batch. The nucleoside to base ion mass transitions of 282 to 150 (m^6^A) and 268 to 136 (A) were used for quantification. The ratio of m^6^A to A was calculated based on the calibrated concentrations. For the enrichment of poly(A) + mRNA from total RNA, the Dynabeads mRNA DIRECT Purification Kit (Invitrogen, 61012) was used, following the manufacturer’s instructions. 18*S* rRNA and 28*S* rRNA in total RNA were separated by agarose gel electrophoresis and recovered from the gel using the Zymoclean Gel RNA Recovery Kit (Zymo Research, R1011).

### MeRIP-m^6^A-seq and RNA-seq

Total RNAs were extracted from the forebrain of E13.5 mice using TRIzol reagent. To ensure accurate quantification, 1 μl of spike-in control RNA was added to 5 μg of total RNA, and the RNAs were treated with RNase-free DNase I for 20 min at 37°C to remove DNA contamination. Next, the RNAs were fragmented by adding the RNA fragmentation buffer and incubating for 5 min at 70°C. The fragmented RNAs were then recovered using the RNA Clean & Concentrator-5 kit (Zymo Research, R1014) and subjected to IP with an anti-m^6^A antibody (Millipore, ABE572) and protein-A&G magnetic beads in IP buffer [10 mM tris-HCl (pH 7.4), 150 mM NaCl, and 0.1% IGEPAL CA-630, supplemented with RNase inhibitor] for 2 hours at 4°C. One-tenth of the fragmented RNAs were saved as “input.” After the IP, the beads were washed in IP buffer, and the RNAs were eluted using m^6^A monophosphate solution. The eluted RNAs, along with the input RNAs, were recovered using the RNA Clean & Concentrator-5 kit and used for library preparation with the SMARTer Stranded Total RNA-Seq Kit v2–Pico Input Mammalian (Takara Bio, 634413). This library preparation kit includes an rRNA depletion method. The resulting libraries were subjected to PE150 (paired-end 150 bp) sequencing at Nanjing Jiangbei New Area Biopharmaceutical Public Service Platform Co. Ltd. Four biological replicates were performed for each genotype.

### MeRIP-m^6^A-seq and RNA-seq data analyses

Reads were quality controlled using FastQC (v0.11.5) and TrimGalore (v0.6.0) with default parameters. Removal of rRNA reads was achieved using Bowtie2 (v2.3.4.1) with the “--un-conc-gz” parameter. Filtered reads were aligned using HISAT2 (v2.2.1) with the “--rna-strandness RF” parameter, resulting in 33,399,139 to 63,403,966 reads aligned to the GRCm38 genome (table S1). For RNA-seq data analysis, gene expression levels were quantified using featureCounts (v1.6.0). Differential expression analysis was performed using R package DESeq2 (v1.38.3). Genes with baseMean > 20, |Log_2_FC| ≥ 0.5, and FDR < 0.05 were considered as differentially expressed genes (DEGs). For AS analysis, including the exon inclusion level analysis, we used rMATS (v4.0.3beta). For APA analysis, we used the DaPars algorithm (*30*). APA events that met the criteria of FDR ≤ 0.05, absolute ΔPDUI ≥ 0.2, and at least twofold change of PUDIs were defined as significantly changed. For MeRIP-m^6^A-seq data analysis, m^6^A peaks were called using the R package exomePeak2 (v1.10.0). Peaks with |DiffModLog_2_FC| ≥ 0.5 and FDR < 0.05 were considered as differential m^6^A peaks between WT and cKO groups. All BAM files were converted to bigWigs for visualization in Integrative Genomics Viewer (IGV, v2.8.2) by using the bamCoverage command in deepTools (v3.5.1). The consensus m^6^A motifs were determined using the HOMER (v4.11) motif discovery tool. Metagene plot of m^6^A was performed using the R package Guitar (v2.14.0). For heatmap analysis and summary plot of the MeRIP-m^6^A-seq read density, the computeMatrix in deepTools suit (v3.5.1) (*80*) was taken for computing with “--regionBodyLength 6000, --beforeRegionStartLength 3000, --afterRegionStartLength 3000, --binSize 300, --skipZeros” parameters, followed by the visualization using plotHeatmap and plotProfile with default parameters.

### Quantitative proteomic analysis

To perform quantitative proteomic analysis, protein lysates were extracted from the forebrain of E13.5 mice using RIPA buffer. The subsequent analysis was conducted at Jingjie PTM BioLab (Hangzhou) Co. Ltd. The protein concentration was determined using the Pierce BCA Protein Assay Kit, and the samples underwent sequential trypsin digestion, LC-MS/MS, and data analysis to obtain comprehensive protein profiles. For trypsin digestion, equal amounts of proteins from each sample were used, and the volume was adjusted with lysis buffer to ensure consistency. Prechilled acetone was added in an equal volume to the sample, followed by vortex mixing. An additional fourfold volume of prechilled acetone was added, and the mixture was precipitated at −20°C for 2 hours. After centrifugation at 4500*g* for 5 min, the precipitate was washed twice with prechilled acetone. The resulting pellet was air-dried and then resuspended in 200 mM Triethylammonium bicarbonate (TEAB) buffer. The precipitate was sonicated for dispersion, and trypsin was added at a ratio of 1:50 (enzyme to protein, m/m) for overnight digestion. For reduction, 5 mM dithiothreitol (DTT) was added, and the mixture was incubated at 56°C for 30 min. Subsequently, 11 mM iodoacetamide was added, and the mixture was incubated at RT in the dark for 15 min for alkylation. The resulting peptides were then separated using ultrahigh-performance liquid chromatography (UHPLC) and ionized in the NSI ion source before undergoing MS/MS in the Orbitrap Exploris 480 mass spectrometer, which was coupled online to the UHPLC. The acquired MS/MS data were processed using the Proteome Discoverer (v2.4.1.15) search engine. Four biological replicates were performed for each genotype. Proteins with *P* value < 0.05 and fold change (FC) ≥ 1.2 or FC ≤ 1/1.2 were considered differentially expressed.

### MeRIP combined with RT-qPCR

MeRIP-qPCR was performed to quantify m^6^A modifications on target mRNAs. Total RNA was extracted from tissues using TRIzol reagent (Invitrogen, 15596026) following the manufacturer’s instructions. For each IP reaction, 33 μg of total RNAs was fragmented by adding RNA fragmentation buffer (Invitrogen, AM8740) and incubating for 3 min at 70°C. The fragmented RNAs were then recovered using ethanol precipitation-based method with three volumes of ethanol, one-tenth volume of 3 M sodium acetate, and 1 μl of glycogen (Thermo Fisher Scientific, R0551). Subsequently, the fragmented RNAs were subjected to IP using an anti-m^6^A antibody (Millipore, ABE572) or a control IgG antibody (Millipore, PP64) along with protein-A&G magnetic beads (Thermo Fisher Scientific, 88803) in IP buffer [50 mM tris-HCl (pH 7.4), 150 mM NaCl, and 0.5% IGEPAL CA-630, supplemented with RNase inhibitor] for 2 hours at 4°C. One-tenth of the fragmented RNAs were saved as “input.” Following IP, the beads were washed with IP buffer, and the bound RNAs were released by incubation in proteinase K reaction buffer [100 mM tris-HCl (pH 7.4), 50 mM NaCl, 1 mM EDTA, 0.2% SDS, and proteinase K (0.95 mg/ml)] for 30 min at 55°C. The released RNAs were then recovered using ethanol precipitation-based method. The RNAs obtained from the IP, as well as the input RNAs, were used for cDNA library construction and subsequent RT-qPCR analysis. To quantify the enrichment of immunoprecipitated RNA relative to the input (% Input), the Ct value and the sample volume were taken into account during calculations. Four biological replicates were performed for each group. No PCR amplification was detected in the negative IP samples using the control IgG antibody. The primers used for MeRIP-qPCR are listed in table S5.

### RNA immunoprecipitation with RT-qPCR

RIP experiment was conducted using the EZ-Magna RIP RNA-Binding Protein Immunoprecipitation Kit (Millipore, 17-701) following the manufacturer’s instructions. To optimize the cell lysis step, cultured NPCs (15 million cells in each sample) were lysed with 100 μl of complete RIP lysis buffer supplemented with 900 μl of RIP IP buffer, along with a protease inhibitor cocktail, for 60 min at 4°C. After clearing the cell lysates by centrifugation at 18,000*g* at 4°C for 20 min, we proceeded with IP using a rabbit anti-YTHDF2 antibody (Proteintech, 24744-1-AP) as well as a control rabbit IgG. One-tenth of the cleared cell lysates were saved as “input.” The eluted RNAs from the IP and the input RNA were used for cDNA library construction and subsequent RT-qPCR analysis. To quantify the enrichment of immunoprecipitated RNA relative to the input (% Input), the Ct value and the sample volume were taken into account during calculations. The results for each group were obtained from two biological replicates. The primers used for RT-qPCR are listed in table S5.

### In vivo IP

Forebrain tissues from more than 20 mice at E13.5 were combined and lysed in ice-cold lysis buffer [50 mM tris-HCl (pH 7.4), 1% IGEPAL CA-630, 5 mM MgCl_2_, and 150 mM KCl] supplemented with a protease inhibitor cocktail (MCE, HY-K0010) and phosphatase inhibitor cocktails (MCE, HY-K0021 and HY-K0022). The lysates were incubated at 4°C for 2 to 3 hours and then cleared by centrifugation at 18,000*g* at 4°C for 20 min. The protein concentration was determined using the Pierce BCA Protein Assay Kit (Thermo Fisher Scientific, 23227). Equal amounts of protein in equal volumes were subjected to IP using an anti-MDM2 antibody (Santa Cruz sc-377317) or a control IgG antibody along with protein-A&G magnetic beads (Thermo Fisher Scientific, 88803) in lysis buffer for 3 hours at 4°C. Bound proteins were washed twice with lysis buffer and then eluted with SDS loading buffer. The eluted proteins were used for WB analysis.

### Statistical analysis

To quantify protein expression, the chemiluminescence intensity of each protein was first normalized to the intensity of tubulin or actin on the same membrane. Subsequently, the normalized value of the WT group was set as the average value of 1 for comparison. GO term and KEGG pathway enrichment analyses of DEGs were performed using the bioinformatics tool DAVID (https://david.ncifcrf.gov/). To measure the area with a signal in brain slices [unit: cluster area (μm^2^) per 1000 μm^2^], the compared images were thresholded using a constant threshold value in ImageJ. This threshold value was calculated as the mean of the manually selected threshold values, which were visually confirmed to select appropriate signal clusters. For the ratio of cells with a signal in NPCs, the compared images were thresholded using a constant threshold of fluorescent signal intensity in ImageJ. The number of cells with a signal above the threshold was manually counted, and this count was then divided by the count of Hoechst-stained cells to obtain the ratio. In fig. S5, the intensity value in each pixel was obtained along an 18-μm line spanning a cell nucleus using Zen software (V.2.3; Carl Zeiss Inc.). These data were used to generate a fluorescence intensity profile. The mean fluorescence intensity per micrometer for each cell was calculated and used for statistical comparison between WT and cKO groups. Adobe Photoshop CS6 was used to adjust the brightness and contrast of the images and to select the regions of interest. GraphPad Prism 8.0.1 was used to perform statistical analysis and plot the data. The statistical analysis methods and the sample sizes are indicated in the figure legends. Results are expressed as the means ± SEM. Significant differences are denoted by asterisks (*P < 0.05, **P < 0.01, and ***P < 0.001). No statistical methods were used to predetermine sample sizes, but our sample sizes were similar to those reported in previous publications (*11*, *81*).
