# Supplementary material for: VIRMA-mediated m6A modification regulates forebrain formation through modulating ribosome biogenesis
Source: Sci Adv. 2025 Jun 27;11(26):eadq9643. doi: 10.1126/sciadv.adq9643 (PMC12204135; doi:10.1126/sciadv.adq9643)
Supplement: Supplementary file 1 — Figs. S1 to S16 Legends for tables S1 to S5 [file sciadv.adq9643_sm.pdf]

Science Advances
AAAS

Supplementary Materials for

VIRMA-mediated m^6A modification regulates forebrain formation through modulating ribosome biogenesis

Min Wu *et al.*

Corresponding author: Min Wu, min.wu@siat.ac.cn; Bin Shen, binshen@njmu.edu.cn;
Tao Zhou, zhoutao@siat.ac.cn

Sci. Adv. **11**, eadq9643 (2025)
DOI: 10.1126/sciadv.adq9643

The PDF file includes:

Figs. S1 to S16
Legends for tables S1 to S5

Other Supplementary Material for this manuscript includes the following:

Tables S1 to S5

Figure S1

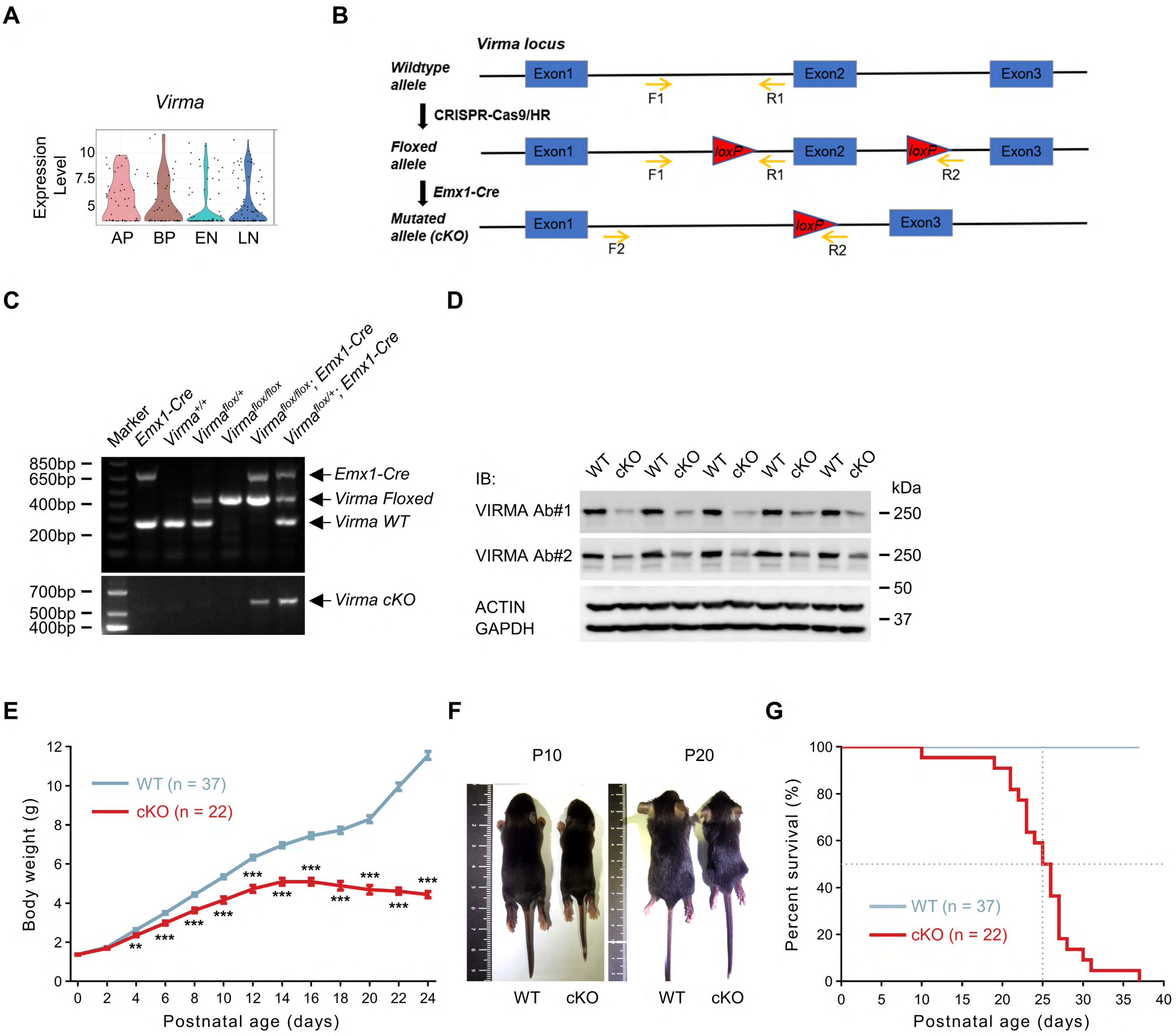

A
Virma
Expression Level
10
7.5
5
AP
BP
EN
LN
B
Virma locus
Wildtype allele
Exon1
Exon2
Exon3
F1
R1
CRISPR-Cas9/HR
Floxed allele
loxP
R2
Emx1-Cre
Mutated allele (cKO)
F2
C
Marker
Emx1-Cre
Virma+/+
Virmaflox/+
Virmaflox/flox
Virmaflox/flox; Emx1-Cre
Virmaflox/+; Emx1-Cre
850bp
650bp
400bp
200bp
700bp
500bp
400bp
Emx1-Cre
Virma Floxed
Virma WT
Virma cKO
D
IB:
WT
cKO
kDa
VIRMA Ab#1
VIRMA Ab#2
ACTIN
GAPDH
250
250
50
37
E
Body weight (g)
Postnatal age (days)
WT (n = 37)
cKO (n = 22)
F
P10
P20
WT
cKO
G
Percent survival (%)
Postnatal age (days)
WT (n = 37)
cKO (n = 22)

Fig. S1. Generation and characterization of *Emx1-Cre*-mediated *Virma* cKO mice.

(**A**) VIRMA expression in various cell types, including NPCs and their progeny, based on a publicly available single-cell RNA-seq dataset (*24*). VIRMA exhibited high expression in NPCs. Violin plots were generated on the website http://genebrowser.unige.ch/science2016/. AP: Apical progenitors; BP: daughter basal progenitors; EN: early neurons; LN: late neurons.
(**B**) Schematic illustration of the procedure for generating *Emx1-Cre*-mediated *Virma* cKO mice. Exon 2, flanked by two loxP sites (red), can be deleted upon Cre expression. F1 and R1, F2 and R2: primer pairs used for genotyping PCR; loxP: locus of X-over P1; floxed: an allele with two forward loxP sequences; HR: homologous recombination.
(**C**) Genotyping analysis of mice with different genotypes, including *Emx1-Cre, Virma*$^{+/+}$, *Virma*$^{flox/+}$, *Virma*$^{flox/flox}$, *Virma*$^{flox/flox}$*; Emx1-Cre,* and *Virma*$^{flox/+}$*; Emx1-Cre*. *Virma*$^{flox/+}$: *Virma* gene with one floxed allele and one wildtype (+) allele; *Virma*$^{flox/flox}$: *Virma* gene with two floxed alleles; *Emx1-Cre*: Cre recombinase expression driven by *Emx1* promoter.
(**D**) Western blot validation of *Virma* knockout using VIRMA antibody #1 (Ab#1) and VIRMA Ab#2, confirming depletion of VIRMA protein in forebrain homogenates from VIRMA cKO mice at E13.5. Faint bands remaining are attributed to contamination from normal surrounding tissue.
(**E**) Body weights of VIRMA WT (n = 37) and cKO (n = 22) mice during the first 24 days after birth. Starting from P4, the body weights of cKO mice were significantly less than those of WT mice. Unpaired Student's t-test, $^{**}p < 0.01$, $^{***}p < 0.001$, compared with WT mice. Data are mean ± SEM.
(**F**) Body size comparison of VIRMA WT and cKO mice at P10 and P20. VIRMA cKO mice were much smaller in size than their WT littermates.
(**G**) Kaplan-Meier survival curves of VIRMA WT (n = 37) and cKO (n = 22) mice showing the survival rates. The median survival time of VIRMA cKO mice was 25 days. Log-rank (Mantel-Cox) Test, $p < 0.0001$.

Figure S2

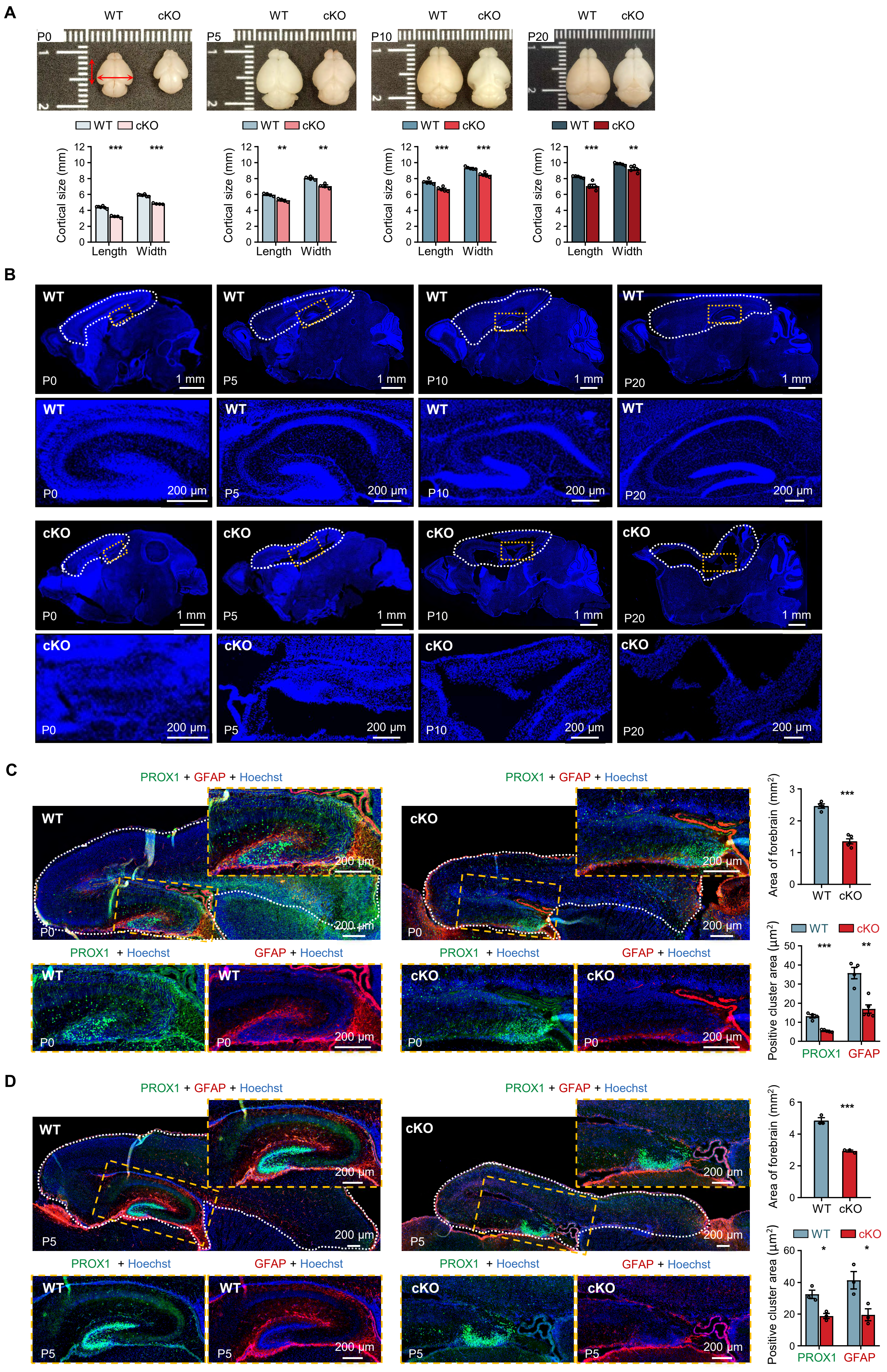

A
WT
cKO
P0
P5
P10
P20
WT cKO
Cortical size (mm)
Length
Width
***
**
B
WT
cKO
1 mm
200 μm
C
PROX1 + GFAP + Hoechst
PROX1 + Hoechst
GFAP + Hoechst
Area of forebrain (mm²)
Positive cluster area (μm²)
PROX1
GFAP
D
*

Fig. S2. Severe developmental defects in the forebrain of postnatal VIRMA cKO mice.

(**A**) Gross anatomy of brains from VIRMA WT and cKO mice at P0, P5, P10, and P20; quantitative analysis of cortical length and width (red arrows). N = 4-6 mice for each genotype.

(**B**) Representative images of Hoechst staining in sagittal brain sections from VIRMA WT and cKO mice at P0, P5, P10, and P20. The regions delineated by irregular dashed white lines indicate the forebrain area. Regions enclosed by dashed yellow boxes are shown at higher magnification.

(**C** to **D**) Immunofluorescence for the dentate gyrus granule cell marker PROX1 (green) and the astrocytic marker GFAP (red) in sagittal brain sections from VIRMA WT and cKO mice at P0 and P5. Nuclei were counterstained with Hoechst. The regions delineated by irregular dashed white lines indicate the forebrain area used for quantification. The area (μm^2) with positive signal was markedly decreased in VIRMA cKO mice compared to WT mice. N = 3-5 mice for each genotype. Regions enclosed by dashed yellow boxes are shown at higher magnification.

Unpaired Student's t-test in (A), (C) and (D), *p < 0.05, **p < 0.01, ***p < 0.001, compared with WT mice. Data are mean ± SEM.

Figure S3

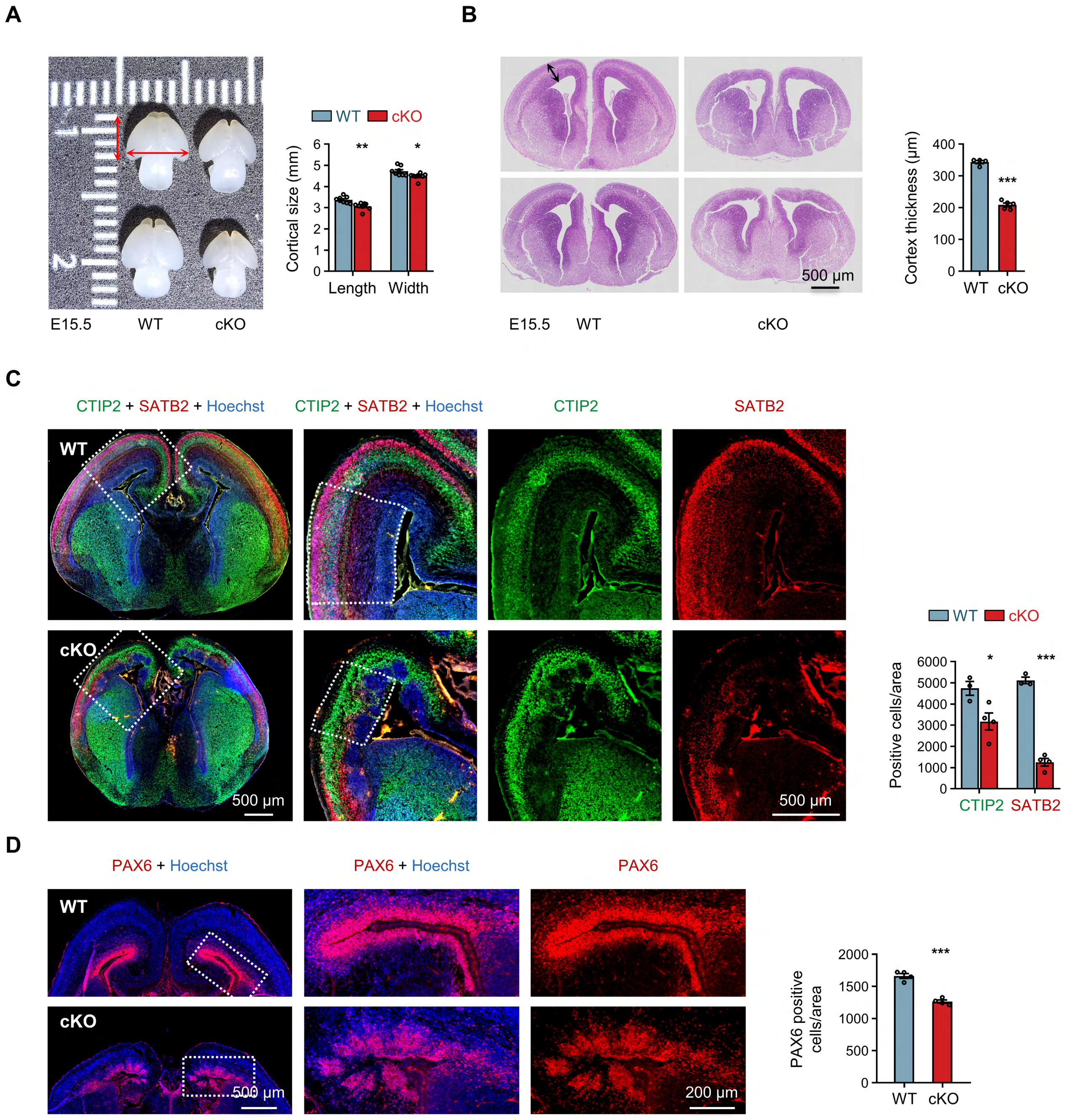
A
E15.5
WT
cKO
WT
cKO
Cortical size (mm)
**
*
Length
Width
B
500 μm
E15.5
WT
cKO
Cortex thickness (μm)
***
WT
cKO
C
CTIP2 + SATB2 + Hoechst
CTIP2 + SATB2 + Hoechst
CTIP2
SATB2
WT
cKO
500 μm
500 μm
WT
cKO
Positive cells/area
*
***
CTIP2
SATB2
D
PAX6 + Hoechst
PAX6 + Hoechst
PAX6
WT
cKO
500 μm
200 μm
PAX6 positive cells/area
***
WT
cKO

Fig. S3. Severe developmental defects in the forebrain of embryonic VIRMA cKO mice.

(**A**) Whole brain images of VIRMA WT and cKO mice at E15.5; quantitative analysis of cortical length and width (red arrows). N = 8 mice for each genotype.

(**B**) Hematoxylin and eosin (H&E) stained coronal brain sections from VIRMA WT and cKO mice at E15.5; quantitative analysis of cortical thickness (black arrows). N = 4-5 mice for each genotype.

(**C**) Immunofluorescence for the deep layer cortical neuron marker CTIP2 (green) and the upper layer cortical neuron marker SATB2 (red) in coronal brain sections from VIRMA WT and cKO mice at E17.5. Nuclei were counterstained with Hoechst. VIRMA cKO mice exhibited disorganized structure and reduced number of CTIP2- and SATB2-positive cells in the forebrain. The number of CTIP2-positive or SATB2-positive cells within the regions delineated by irregular dashed white lines was manually counted based on n = 3-4 images. Regions enclosed by dashed white boxes are shown at higher magnification.

(**D**) Immunostaining of coronal brain sections from VIRMA WT and cKO mice at E17.5 with an antibody against PAX6, a well-characterized neural stem cell marker. Nuclei were counterstained with Hoechst. The number of PAX6-positive cells within the regions delineated by dashed white boxes was manually counted. A significant decrease and disorganization of neural stem cells were observed in the forebrain of VIRMA cKO mice at E17.5. N = 4 mice for each genotype. Regions enclosed by dashed white boxes are shown at higher magnification.

Unpaired Student's t-test in (A to D), $*p < 0.05$, $**p < 0.01$, $***p < 0.001$, compared with WT mice. Data are mean ± SEM.

Figure S4

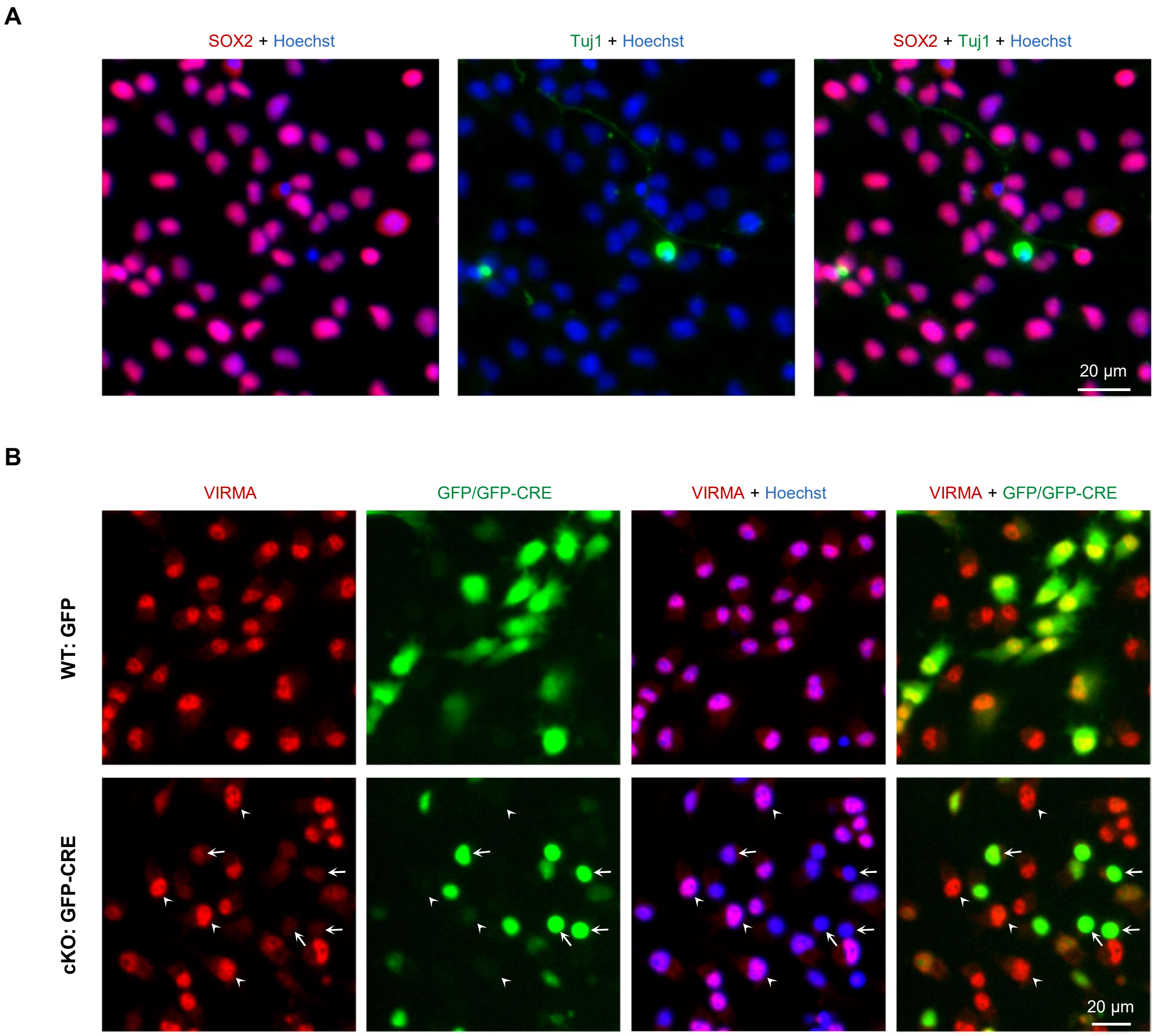
A
SOX2 + Hoechst
Tuj1 + Hoechst
SOX2 + Tuj1 + Hoechst
20 μm
B
VIRMA
GFP/GFP-CRE
VIRMA + Hoechst
VIRMA + GFP/GFP-CRE
WT: GFP
cKO: GFP-CRE
20 μm

Fig. S4. Characterization of primary cultured NPCs.

(**A**) Immunostaining revealed high expression level of the stem marker SOX2 and low expression level of the neuronal differentiation marker βIII tubulin (Tuj1) in cultured NPCs. Nuclei were counterstained with Hoechst.

(**B**) Immunostaining of cultured WT and VIRMA cKO NPCs infected with lentivirus expressing GFP or GFP-CRE, respectively, using a VIRMA antibody. Non-infected WT NPCs and infected VIRMA cKO NPCs expressing GFP-CRE are denoted by arrowheads and arrows, respectively. Nuclei were counterstained with Hoechst.

Figure S5

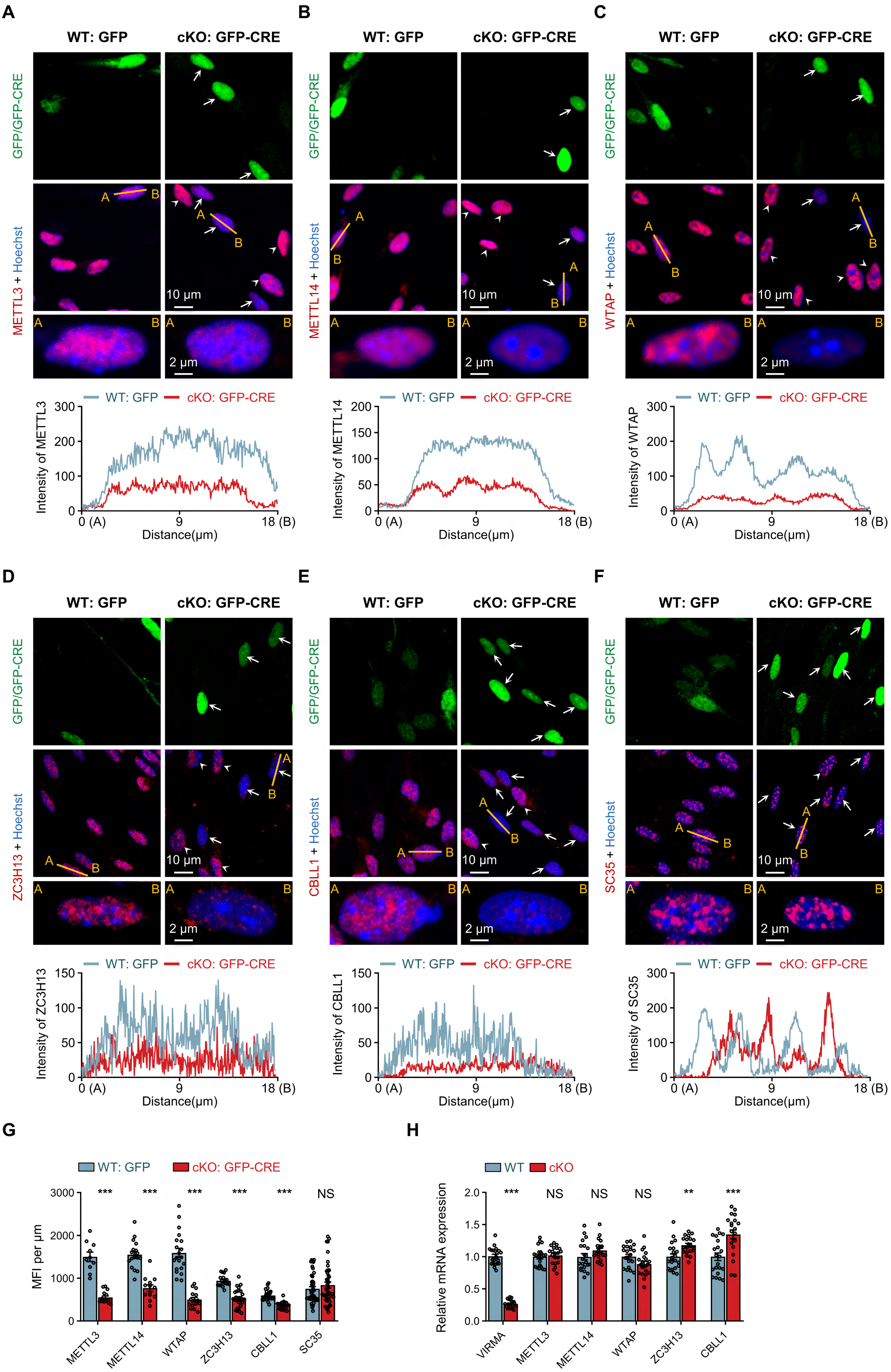

A
B
C
D
E
F
G
H
WT: GFP
cKO: GFP-CRE
GFP/GFP-CRE
METTL3 + Hoechst
METTL14 + Hoechst
WTAP + Hoechst
ZC3H13 + Hoechst
CBLL1 + Hoechst
SC35 + Hoechst
10 μm
2 μm
Intensity of METTL3
Intensity of METTL14
Intensity of WTAP
Intensity of ZC3H13
Intensity of CBLL1
Intensity of SC35
Distance(μm)
0 (A)
9
18 (B)
MFI per μm
Relative mRNA expression
WT
cKO
***
**
NS
METTL3
METTL14
WTAP
ZC3H13
CBLL1
SC35
VIRMA

Fig. S5. Nuclear localization of m^{6}A writer components in NPCs requires VIRMA.

(**A** to **F**) Immunostaining of cultured WT and VIRMA cKO NPCs infected with lentivirus expressing GFP or GFP-CRE, respectively, using indicated antibodies. Non-infected WT NPCs and infected VIRMA cKO NPCs expressing GFP-CRE are denoted by arrowheads and arrows, respectively, in the corresponding image. For each condition, one representative cell is shown at higher magnification, and a fluorescence intensity profile was plotted along an 18 µm line (A→B) spanning a cell nucleus to display the expression levels of the indicated protein. Nuclei were counterstained with Hoechst.

(**G**) Mean fluorescence intensity (MFI) per µm for different proteins localizing at nucleus speckles, as shown in (A to F), was calculated for each cell and used for statistical comparison. N = 10-46 cells for each condition.

(**H**) RT-qPCR analysis of mRNA levels of m^{6}A writer components in the forebrain of E13.5 WT and VIRMA cKO mice. The Ct values were first normalized to GAPDH control, which were similar in both WT and cKO samples. N = 20-21 samples for each genotype.

Unpaired Student's t-test in (G) and (H), $*p < 0.05$, $**p < 0.01$, $***p < 0.001$, compared with WT control. Data are mean ± SEM.

Figure S6

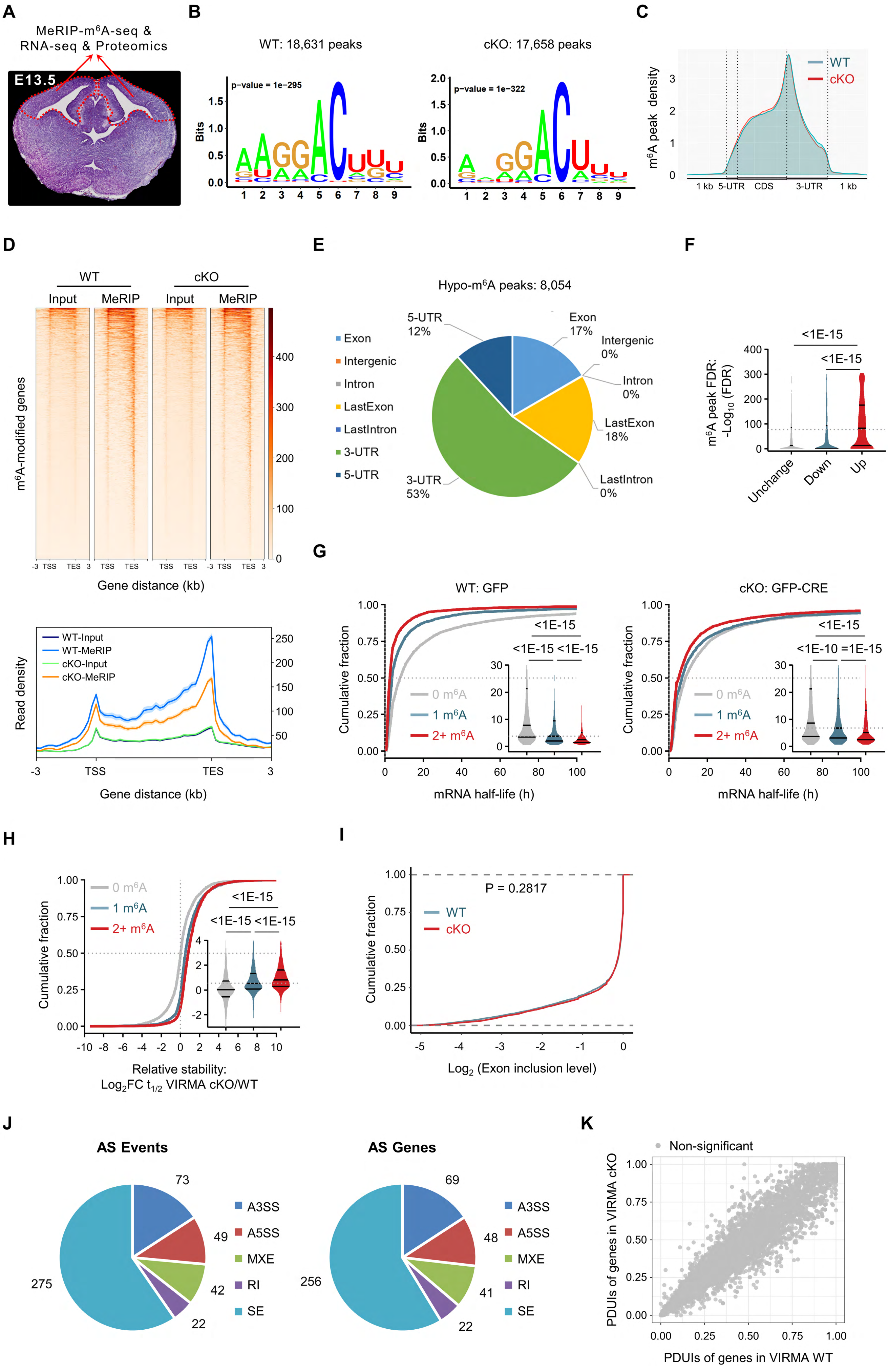

A
MeRIP-m6A-seq &
RNA-seq & Proteomics
E13.5
B
WT: 18,631 peaks
cKO: 17,658 peaks
p-value = 1e-295
p-value = 1e-322
Bits
C
m6A peak density
WT
cKO
1 kb 5-UTR CDS 3-UTR 1 kb
D
WT
cKO
Input
MeRIP
m6A-modified genes
Gene distance (kb)
WT-Input
WT-MeRIP
cKO-Input
cKO-MeRIP
Read density
E
Hypo-m6A peaks: 8,054
Exon
Intergenic
Intron
LastExon
LastIntron
3-UTR
5-UTR
5-UTR 12%
Exon 17%
Intergenic 0%
Intron 0%
LastExon 18%
LastIntron 0%
3-UTR 53%
F
m6A peak FDR: -Log10 (FDR)
<1E-15
Unchange
Down
Up
G
WT: GFP
cKO: GFP-CRE
Cumulative fraction
0 m6A
1 m6A
2+ m6A
<1E-10
=1E-15
mRNA half-life (h)
H
Relative stability: Log2FC t1/2 VIRMA cKO/WT
I
P = 0.2817
Log2 (Exon inclusion level)
J
AS Events
AS Genes
A3SS
A5SS
MXE
RI
SE
K
Non-significant
PDUIs of genes in VIRMA cKO
PDUIs of genes in VIRMA WT

Fig. S6. MeRIP-m^{6}A-seq and RNA-seq analyses of E13.5 forebrain from WT and VIRMA cKO mice.

(**A**) Diagram illustrating the forebrain region dissected from E13.5 mice for MeRIP-m^{6}A-seq, RNA-seq, and proteomics analyses.

(**B**) Enriched m^{6}A RRACH motifs in mRNAs from E13.5 forebrain of VIRMA WT and cKO mice, detected by the HOMER motif discovery tool using the MeRIP-m^{6}A-seq data.

(**C**) Metagene plot displaying the density distribution of m^{6}A peaks across 5-UTR, CDS, and 3-UTR regions of mRNAs, normalized for length. Transcript models were extended 1,000 nucleotides (1 kb) upstream and downstream of 5-UTR and 3-UTR, respectively.

(**D**) Heatmaps (upper panel) and summary plots (lower panel) showing MeRIP-m^{6}A-seq read density of m^{6}A-modified genes. Read counts were extracted from all samples (n=4) for each m^{6}A-modified gene, spanning from the Transcription Start Site (TSS) to the Transcription End Site (TES), and normalized for gene length. Additional regions extending 3,000 nucleotides (3 kb) upstream and downstream of the TSS and TES, respectively, were included. Genes were sorted based on their read density levels in the heatmap. The heatmaps and summary plots collectively reveal that VIRMA depletion resulted in a decrease in m^{6}A levels, particularly evident for peaks located in the 3-UTR regions.

(**E**) Pie chart showing the percentage of Hypo-m^{6}A peaks annotated to each transcript segment. More than half of the Hypo-m^{6}A peaks are distributed in the 3-UTR.

(**F**) False discovery rate (FDR) of m^{6}A peaks from MeRIP-m^{6}A-seq data in the WT forebrain for genes categorized by expression changes between VIRMA WT and cKO forebrain. Down and up represent genes significantly downregulated or upregulated in VIRMA cKO forebrain (FDR < 0.05), respectively. P-value was determined using Wilcoxon rank sum tests.

(**G**) Cumulative distributions and violin plots (inset) showing mRNA half-lives in cultured VIRMA WT (left) and cKO (right) NPCs for mRNA without m^{6}A site (0 m^{6}A), mRNA with one m^{6}A site (1 m^{6}A), and mRNA with two or more m^{6}A sites (2+ m^{6}A).

(**H**) Cumulative distributions and violin plots (inset) showing half-lives change (Log_2FC (cKO/WT)) upon VIRMA depletion in cultured NPCs for mRNA without m^{6}A site (0 m^{6}A), mRNA with one m^{6}A site (1 m^{6}A), and mRNA with two or more m^{6}A sites (2+ m^{6}A).

(**I**) Cumulative distributions showing the exon inclusion level in E13.5 forebrain of VIRMA WT and cKO mice. Differential AS events in RNA-seq data were analyzed using rMATS. Genes in VIRMA cKO forebrain exhibited similar exon inclusion levels compared to WT forebrain. P-value was determined using Wilcoxon signed-rank paired test. The dashed lines at 0 and 1 on the y-axis of the plot represent the boundaries of the cumulative probability range.

(**J**) Pie charts showing the percentage and number of five types of different AS events (left) or genes (right) between WT and VIRMA cKO forebrain identified by rMATs. VIRMA only regulates the AS of a small number of transcripts. SE, skipped exon; RI, retained intron; MXE, mutually exclusive exon; A5SS, alternative 5' splice site; A3SS, alternative 3' splice site.

(**K**) Scattering plots showing the PDUIs (3-UTR usage difference) in the E13.5 forebrain of VIRMA WT and cKO mice. The APA events in the RNA-seq data were analyzed using DaPars algorithm (*30*). No significant changes in APA events were observed between VIRMA WT and cKO forebrain.

P-values were determined using Kruskal-Wallis test followed by Dunn's multiple comparisons test (G and H).

Figure S7

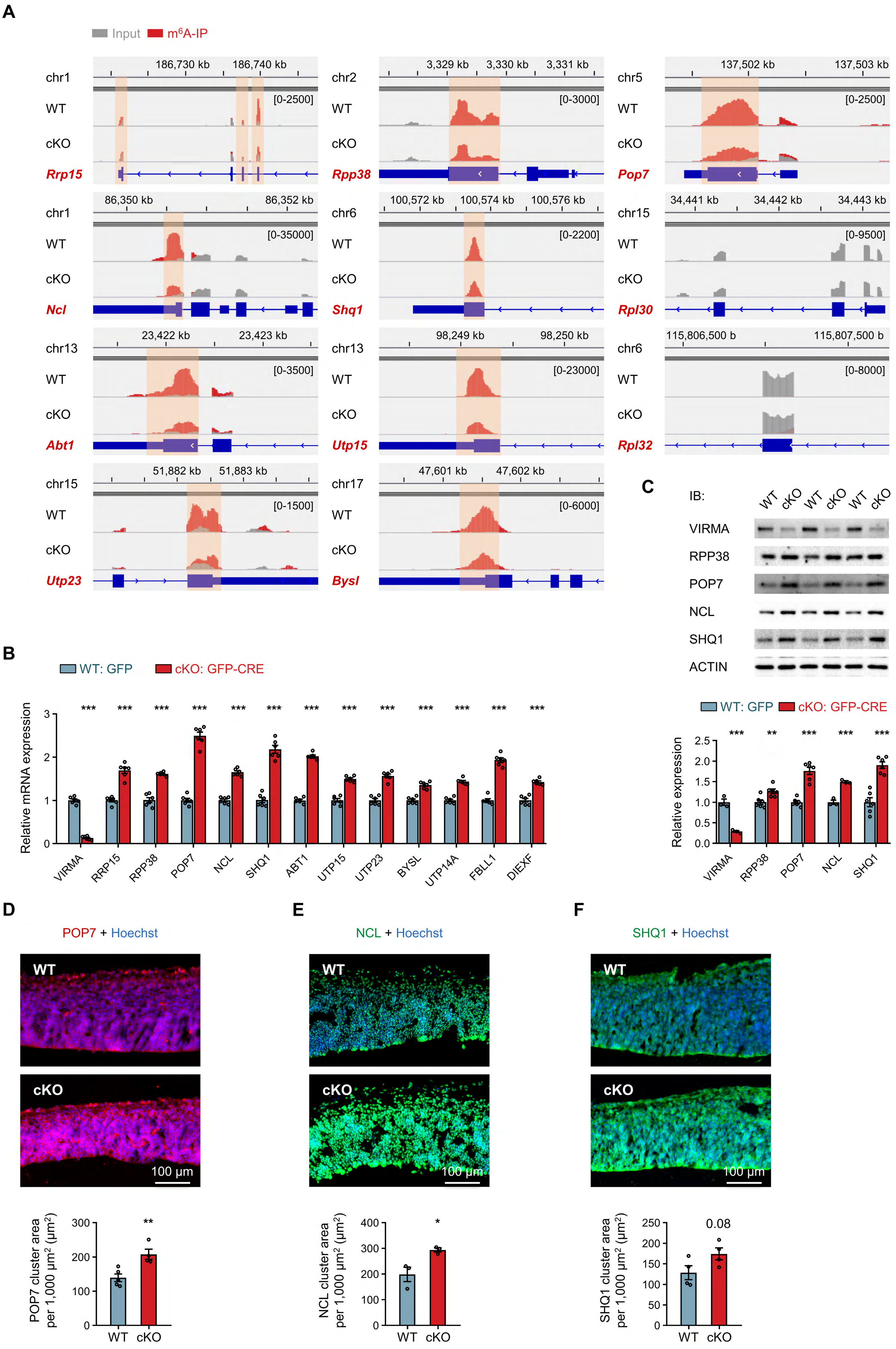
A
Input m6A-IP
Rrp15
Rpp38
Pop7
Ncl
Shq1
Rpl30
Abt1
Utp15
Rpl32
Utp23
Bysl
WT
cKO
B
WT: GFP
cKO: GFP-CRE
Relative mRNA expression
VIRMA
RRP15
RPP38
POP7
NCL
SHQ1
ABT1
UTP15
UTP23
BYSL
UTP14A
FBLL1
DIEXF
C
IB:
VIRMA
RPP38
POP7
NCL
SHQ1
ACTIN
Relative expression
D
POP7 + Hoechst
E
NCL + Hoechst
F
SHQ1 + Hoechst
100 μm
POP7 cluster area per 1,000 μm2 (μm2)
NCL cluster area per 1,000 μm2 (μm2)
SHQ1 cluster area per 1,000 μm2 (μm2)
0.08

Fig. S7. VIRMA deficiency induces upregulation of genes involved in ribosome biogenesis in NPCs.

(**A**) Integrative Genomics Viewer (IGV) screenshots presenting m^6A peaks in a representative MeRIP-m^6A-seq result. Nine genes implicated in ribosome biogenesis (*Rrp15, Rpp38, Pop7, Ncl*, *Shq1, Abt1, Utp15, Utp23* and *Bysl*) with m^6A modifications and two unmethylated genes (*Rpl30* and *Rpl32*) are shown. The red and grey peaks represent the read coverages of immunoprecipitated m^6A and input libraries, respectively. Gene structures are denoted by the blue bar (arrows indicate transcription direction). Orange regions highlight the m^6A peaks downregulated upon VIRMA depletion.

(**B**) RT-qPCR confirming the abnormal upregulation of genes involved in ribosome biogenesis in cultured VIRMA cKO NPCs. All selected genes carry m^6A modification. The Ct values were first normalized to GAPDH control, which were similar in both WT and cKO samples. N = 6 samples for each condition.

(**C**) Western blot validating the abnormal upregulation of genes involved in ribosome biogenesis in cultured VIRMA cKO NPCs. N = 3-6 samples for each genotype.

(**D** to **F**) Immunostaining of coronal brain sections using antibodies against POP7 (D), NCL (E) and SHQ1 (F). Nuclei were counterstained with Hoechst. The area (μm^2 per 1,000 μm^2) with positive signal was significantly increased in E13.5 forebrain of VIRMA cKO mice. N = 3-5 mice for each genotype.

Unpaired Student's t-test in (B to F), *$p < 0.05$, **$p < 0.01$, ***$p < 0.001$, compared with WT samples. Data are mean ± SEM.

Figure S8

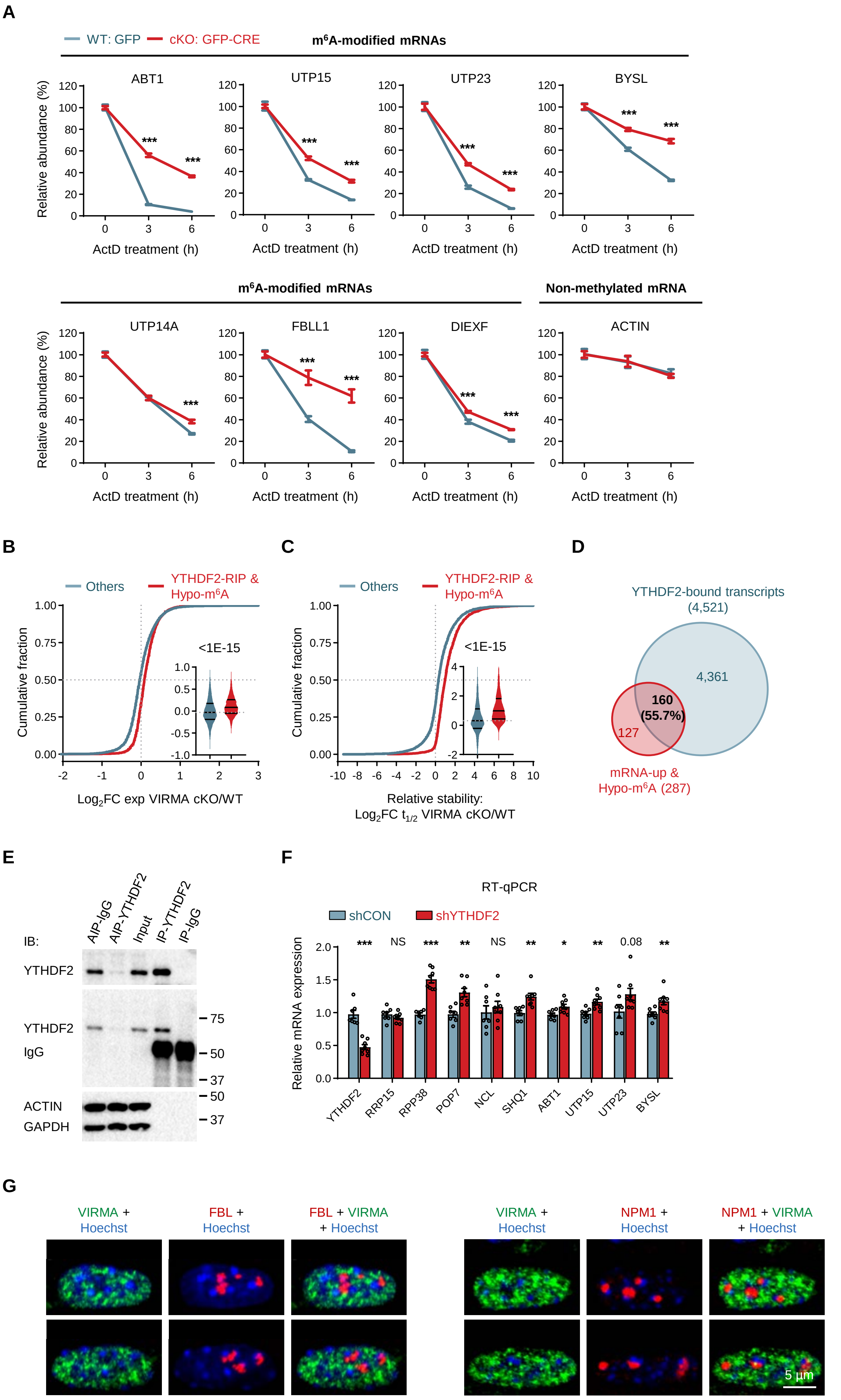

A
WT: GFP
cKO: GFP-CRE
m6A-modified mRNAs
Non-methylated mRNA
ABT1
UTP15
UTP23
BYSL
UTP14A
FBLL1
DIEXF
ACTIN
Relative abundance (%)
ActD treatment (h)
***
B
Others
YTHDF2-RIP & Hypo-m6A
Cumulative fraction
<1E-15
Log2FC exp VIRMA cKO/WT
C
Relative stability: Log2FC t1/2 VIRMA cKO/WT
D
YTHDF2-bound transcripts (4,521)
4,361
160 (55.7%)
127
mRNA-up & Hypo-m6A (287)
E
IB:
AIP-IgG
AIP-YTHDF2
Input
IP-YTHDF2
IP-IgG
YTHDF2
IgG
ACTIN
GAPDH
75
50
37
F
RT-qPCR
shCON
shYTHDF2
Relative mRNA expression
*** NS *** ** NS ** * ** 0.08 **
YTHDF2
RRP15
RPP38
POP7
NCL
SHQ1
ABT1
UTP15
UTP23
BYSL
G
VIRMA + Hoechst
FBL + Hoechst
FBL + VIRMA + Hoechst
VIRMA + Hoechst
NPM1 + Hoechst
NPM1 + VIRMA + Hoechst
5 µm

Fig. S8. VIRMA deficiency enhances the stability of m^6A-modified transcripts involved in ribosome biogenesis in NPCs.

(**A**) RT-qPCR confirming the enhanced stability of m^6A-modified mRNAs upon VIRMA depletion in cultured NPCs. mRNA levels at different time points (0 h, 3 h and 6 h after ActD treatment) were normalized to RPL30 abundance and presented as a percentage of mRNA amount at 0 h. ACTIN mRNAs lacking m^6A sites were used as controls. The stability of mRNAs involved in ribosome biogenesis significantly increased upon VIRMA depletion in NPCs. N =6 samples for each condition.

(**B**) Cumulative distributions and violin plots (inset) showing the difference in expression level fold change (Log_2FC (cKO/WT)) between YTHDF2-bound mRNAs with Hypo-m^6A peaks (YTHDF2-RIP & Hypo-m^6A) and "Others" mRNAs upon VIRMA depletion. "Others" refers to all remaining mRNAs except those that are YTHDF2-bound with Hypo-m^6A peaks. P-value was determined using Wilcoxon rank sum tests.

(**C**) Cumulative distributions and violin plots (inset) showing the half-lives change (Log_2FC (cKO/WT)) upon VIRMA depletion for YTHDF2-bound mRNAs with Hypo-m^6A peaks (YTHDF2-RIP & Hypo-m^6A) and "Others" mRNAs. P-value was determined using Wilcoxon rank sum tests.

(**D**) Venn diagram showing the overlap between YTHDF2-bound transcripts (blue circle, 4,521 genes) and upregulated mRNAs with Hypo-m^6A peaks in E13.5 VIRMA cKO forebrain (red circle, 287 genes). YTHDF2-bound transcripts from a published dataset were identified by YTHDF2-RIP in HeLa cells followed by sequencing (*46*). Numbers indicate the gene counts in each group. The overlapped percentage of "mRNA-up & Hypo-m^6A (287)" genes is indicated.

(**E**) Western blot showing the efficiency of YTHDF2 pull-down in the YTHDF2-RIP experiment using cultured NPCs. YTHDF2 protein abundance in Input, IP-YTHDF2, IP-IgG, AIP-YTHDF2, and AIP-IgG samples were examined. IgG served as a negative control. Input and AIP: homogenates before and after IP, respectively.

(**F**) RT-qPCR analysis assessing the mRNA levels of genes involved in ribosome biogenesis in cultured NPCs infected with lentivirus expressing either shCON or shYTHDF2. All selected genes carry m^6A modification. The Ct values were normalized to RPL30. N = 7-8 samples for each condition.

(**G**) Examination of VIRMA localization in cultured NPCs co-stained for nucleoli markers FBL or NPM1. The majority of the VIRMA protein did not co-localize with FBL or NPM1 in nucleoli.

Unpaired Student's t-test in (A and F), $^*p < 0.05$, $^{**}p < 0.01$, $^{***}p < 0.001$, compared with WT samples. Data are mean ± SEM.

Figure S9

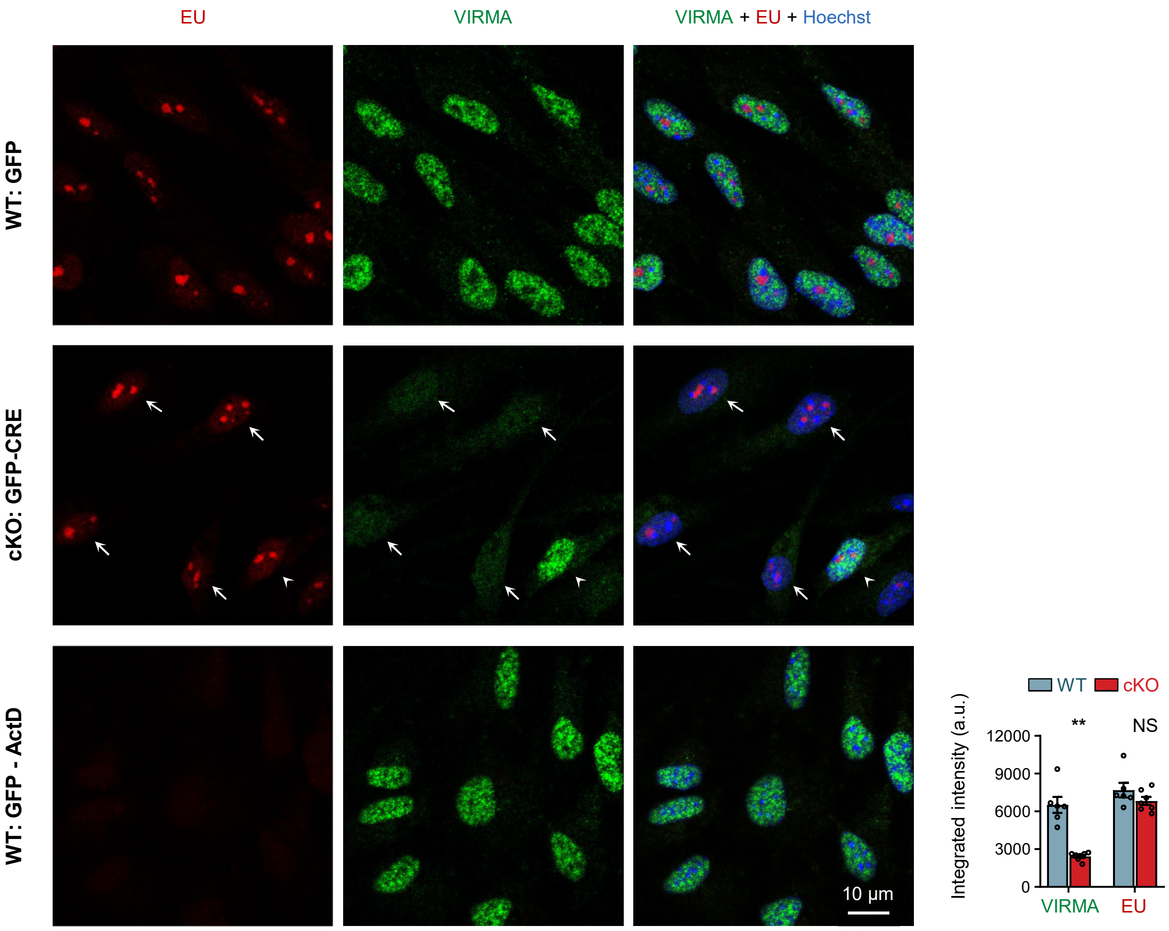

EU
VIRMA
VIRMA + EU + Hoechst
WT: GFP
cKO: GFP-CRE
WT: GFP - ActD
10 µm
WT
cKO
Integrated intensity (a.u.)
12000
9000
6000
3000
0
**
NS
VIRMA
EU

Fig. S9. VIRMA depletion has no effect on rRNA transcription.

Representative images showing rRNA transcription levels in cultured NPCs. rRNA transcription was evaluated through click reaction and imaging after a brief EU labeling pulse. The GFP fluorescence signal was lost due to the click reaction. Accordingly, VIRMA knockout efficiency was visualized by immunostaining with a VIRMA antibody. Non-infected WT NPCs and infected VIRMA cKO NPCs expressing GFP-CRE are denoted by arrowheads and arrows, respectively, in the corresponding image. The faint VIRMA signal in the VIRMA cKO (arrow) NPCs comes from nonspecific background staining. Nuclei were counterstained with Hoechst. Integrated intensity of EU signal in each cell was quantified based on $n = 6\text{-}7$ images.
Unpaired Student's t-test, $^{*}p < 0.05$, $^{**}p < 0.01$, $^{***}p < 0.001$, compared with WT samples. Data are mean ± SEM.

Figure S10

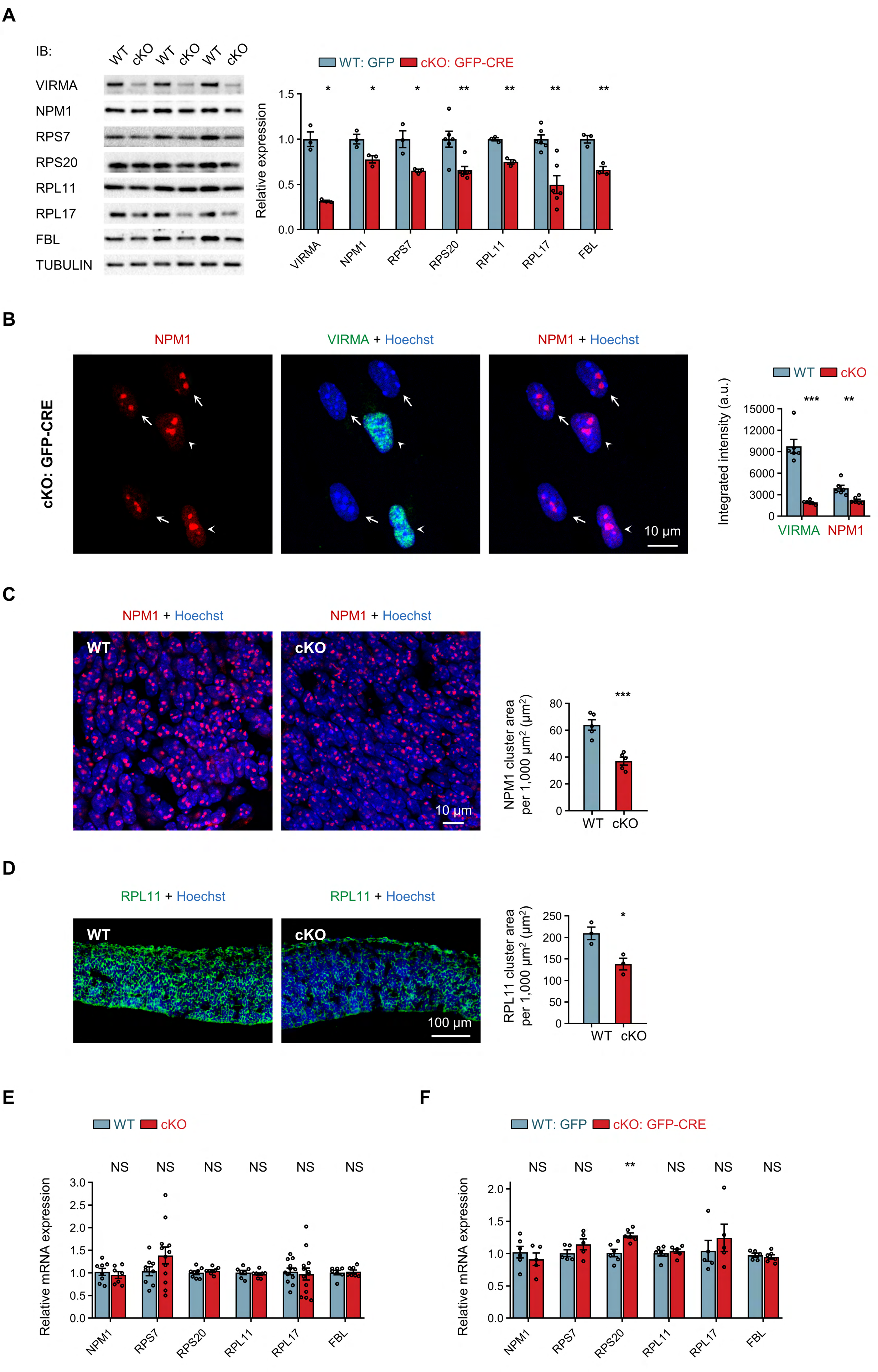

A
IB:
WT cKO WT cKO WT cKO
VIRMA
NPM1
RPS7
RPS20
RPL11
RPL17
FBL
TUBULIN
WT: GFP
cKO: GFP-CRE
Relative expression
* * * ** ** ** **
VIRMA NPM1 RPS7 RPS20 RPL11 RPL17 FBL
B
NPM1
VIRMA + Hoechst
NPM1 + Hoechst
cKO: GFP-CRE
10 μm
WT cKO
Integrated intensity (a.u.)
*** **
VIRMA NPM1
C
NPM1 + Hoechst
NPM1 + Hoechst
WT
cKO
10 μm
NPM1 cluster area per 1,000 μm² (μm²)
***
WT cKO
D
RPL11 + Hoechst
RPL11 + Hoechst
WT
cKO
100 μm
RPL11 cluster area per 1,000 μm² (μm²)
*
WT cKO
E
WT cKO
NS NS NS NS NS NS
Relative mRNA expression
NPM1 RPS7 RPS20 RPL11 RPL17 FBL
F
WT: GFP cKO: GFP-CRE
NS NS ** NS NS NS
Relative mRNA expression
NPM1 RPS7 RPS20 RPL11 RPL17 FBL

Fig. S10. VIRMA depletion leads to a reduction in protein levels of NPM1 and essential ribosome component proteins.

(**A**) Western blot analysis of ribosome-associated proteins in lysates from cultured NPCs with or without VIRMA depletion at 7 days post-lentivirus infection. N = 3-6 samples for each condition.
(**B**) Representative images showing NPM1 signals in cultured WT (arrowhead) and VIRMA cKO (arrow) NPCs. Integrated intensity of NPM1 signal in each cell was quantified based on n = 6 images. Nuclei were counterstained with Hoechst.
(**C**) Representative images showing NPM1 signals in WT and VIRMA cKO forebrain. Nuclei were counterstained with Hoechst. The area (μm^2 per 1,000 μm^2) with NPM1 signal significantly decreased in E13.5 forebrain of VIRMA cKO mice. N = 5 mice for each genotype.
(**D**) Immunostaining of coronal brain sections using an antibody against RPL11, a ribosome component protein. Nuclei were counterstained with Hoechst. The area (μm^2 per 1,000 μm^2) with RPL11 signal significantly decreased in E13.5 forebrain of VIRMA cKO mice. N = 3 mice for each genotype.
(**E**) RT-qPCR analysis of mRNA levels of NPM1, FBL and essential ribosome components, including RPS7, RPS20, RPL11, and RPL17, in the forebrain of E13.5 WT and VIRMA cKO mice. The Ct values were first normalized to GAPDH control, which were similar in both WT and cKO samples. N = 7-13 samples for each genotype.
(**F**) RT-qPCR analysis of mRNA levels of NPM1, FBL and essential ribosome components, including RPS7, RPS20, RPL11, and RPL17, in cultured NPCs. The Ct values were first normalized to GAPDH control, which were similar in both WT and cKO samples. N = 5-6 samples for each genotype.
Unpaired Student's t-test in (A to F), *$p < 0.05$, **$p < 0.01$, ***$p < 0.001$, compared with WT samples. Data are mean ± SEM.

Figure S11

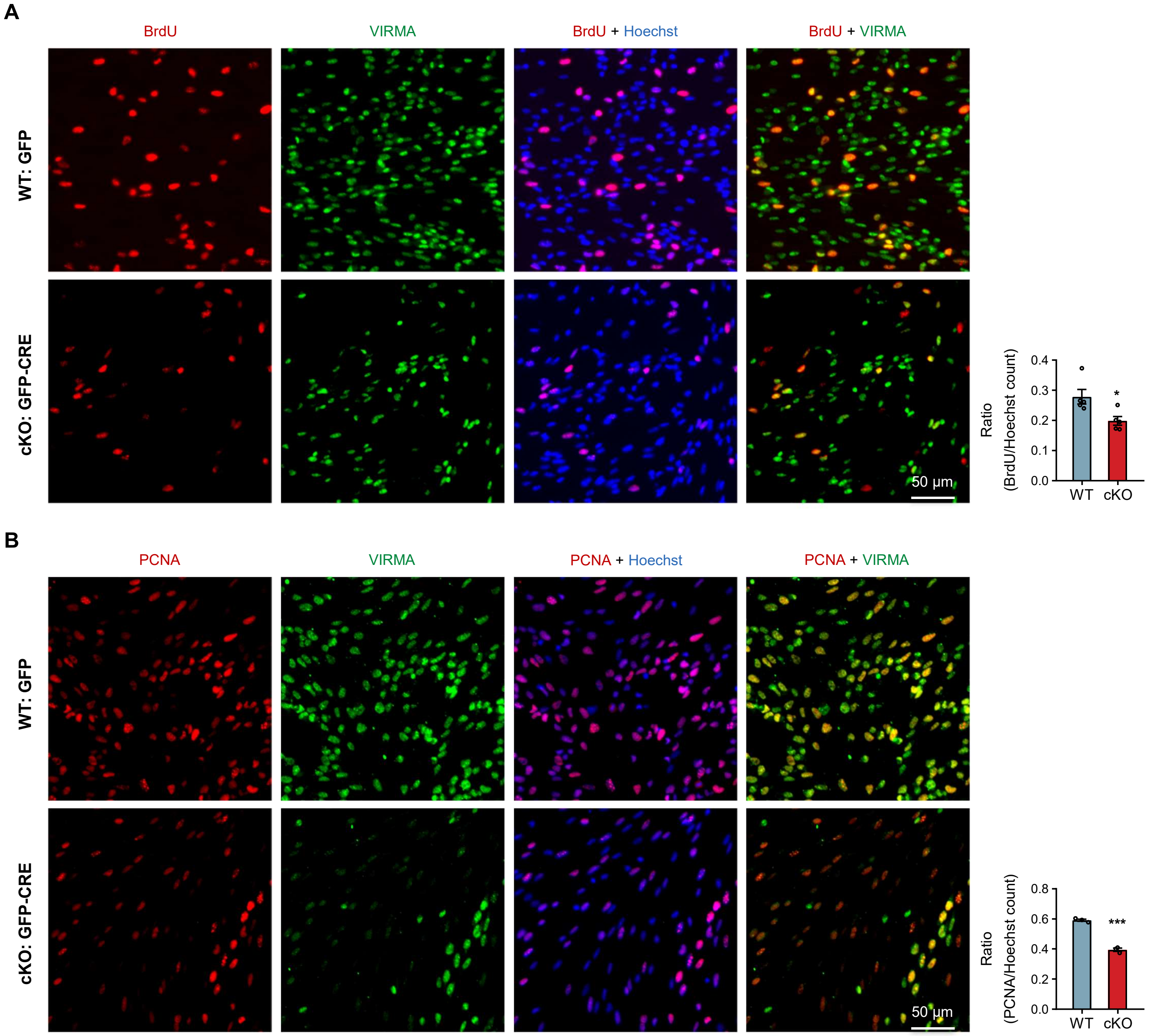
A
BrdU
VIRMA
BrdU + Hoechst
BrdU + VIRMA
WT: GFP
cKO: GFP-CRE
50 µm
Ratio (BrdU/Hoechst count)
0.4
0.3
0.2
0.1
0.0
WT
cKO
*
B
PCNA
VIRMA
PCNA + Hoechst
PCNA + VIRMA
WT: GFP
cKO: GFP-CRE
50 µm
Ratio (PCNA/Hoechst count)
0.8
0.6
0.4
0.2
0.0
WT
cKO
***

Fig. S11. Depletion of VIRMA reduces NPC proliferation.

(**A**) Evaluation of cell proliferation capability through BrdU immunostaining in cultured WT and VIRMA cKO NPCs. On Day 7 after virus infection, NPCs received a 60-minute BrdU pulse before PFA fixation. The GFP fluorescence signal was lost due to antigen retrieval. Accordingly, VIRMA knockout efficiency was visualized by immunostaining with a VIRMA antibody. Nuclei were counterstained with Hoechst. The ratio of BrdU-positive cells (divided by the Hoechst count) with BrdU signal above a certain threshold decreased in VIRMA cKO NPCs compared to WT cells. N = 5 samples for each condition.
(**B**) Immunostaining of cultured WT and VIRMA cKO NPCs using an antibody against PCNA, a marker of cell proliferation. VIRMA knockout efficiency was visualized by immunostaining with a VIRMA antibody. Nuclei were counterstained with Hoechst. The ratio of PCNA-positive cells (divided by the Hoechst count) with PCNA signal above a certain threshold decreased in VIRMA cKO NPCs compared to WT cells. N = 3 samples for each condition.
Unpaired Student's t-test, $*p < 0.05$, $**p < 0.01$, $***p < 0.001$, compared with WT cells. Data are mean ± SEM.

Figure S12

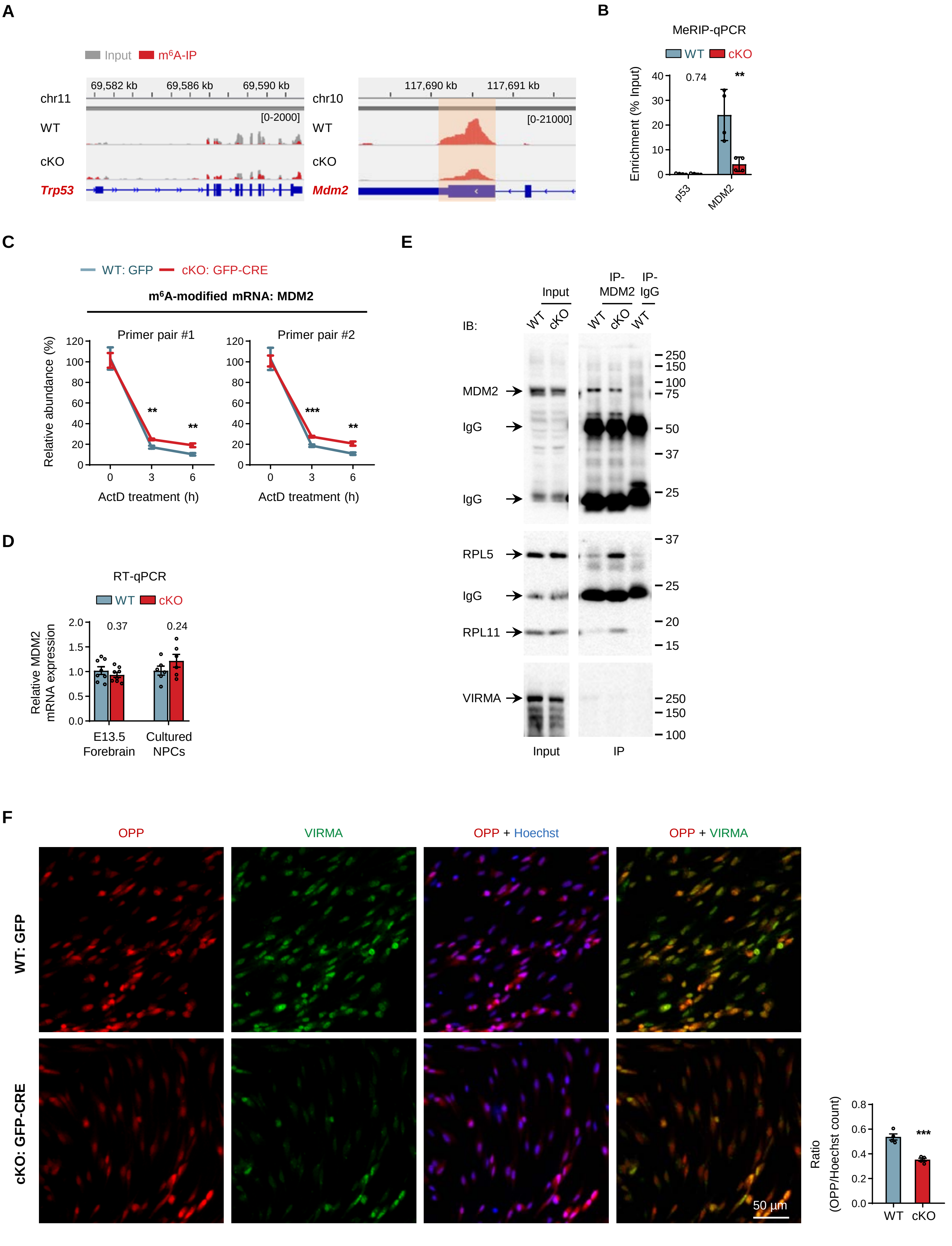
A
Input
m6A-IP
69,582 kb
69,586 kb
69,590 kb
chr11
WT
cKO
[0-2000]
Trp53
117,690 kb
117,691 kb
chr10
[0-21000]
Mdm2
B
MeRIP-qPCR
WT
cKO
Enrichment (% Input)
0.74
**
p53
MDM2
C
WT: GFP
cKO: GFP-CRE
m6A-modified mRNA: MDM2
Primer pair #1
Primer pair #2
Relative abundance (%)
ActD treatment (h)
**
***
D
RT-qPCR
Relative MDM2 mRNA expression
0.37
0.24
E13.5 Forebrain
Cultured NPCs
E
IB:
Input
IP-MDM2
IP-IgG
WT
cKO
MDM2
IgG
RPL5
RPL11
VIRMA
250
150
100
75
50
37
25
20
15
IP
F
OPP
VIRMA
OPP + Hoechst
OPP + VIRMA
WT: GFP
cKO: GFP-CRE
50 µm
Ratio (OPP/Hoechst count)
***
cKO

Fig. S12. Depletion of VIRMA impairs global protein translation.

(**A**) IGV screenshots illustrating the lack of m^6A modification on p53 mRNA and the presence of an m^6A peak on MDM2 mRNA from a representative MeRIP-m^6A-seq result. The red and grey peaks represent the read coverages of immunoprecipitated m^6A and input libraries, respectively. Gene structures are denoted by the blue bar (arrows indicate transcription direction). The orange region highlights the m^6A peak downregulated upon VIRMA depletion.

(**B**) MeRIP-qPCR analysis of m^6A modifications on p53 and MDM2 mRNAs in E13.5 VIRMA WT and cKO forebrain. N = 4 biologically independent experiments.

(**C**) RT-qPCR validation of MDM2 mRNA stability upon VIRMA depletion in cultured NPCs using two different primer pairs. mRNA levels at 0 h, 3 h and 6 h after ActD treatment were normalized to RPL30 abundance and presented as a percentage of mRNA amount at 0 h. The stability of MDM2 mRNA slightly increased, rather than decreased, upon VIRMA depletion in NPCs. N =6 samples for each condition.

(**D**) RT-qPCR analysis of MDM2 mRNA levels in the forebrain of E13.5 WT and VIRMA cKO mice, as well as in cultured WT and VIRMA cKO NPCs. The Ct values were first normalized to GAPDH control, which were similar in both WT and cKO samples. N = 6-8 samples for each genotype.

(**E**) IP with an anti-MDM2 antibody demonstrating that VIRMA depletion in the forebrain enhanced the interaction between MDM2 and ribosomal proteins RPL5 and RPL11. In forebrain homogenates from E13.5 VIRMA cKO mice, RPL5 and RPL11 co-immunoprecipitated more strongly with MDM2 compared to WT mice. IgG served as a negative control.

(**F**) Representative images and quantification of translation rate assessed through OPP click chemistry in cultured NPCs. The GFP fluorescence signal was lost due to the click reaction. Accordingly, VIRMA knockout efficiency was visualized by immunostaining with a VIRMA antibody. Nuclei were counterstained with Hoechst. The ratio of cells (divided by the Hoechst count) with OPP signal above a certain threshold decreased in VIRMA cKO NPCs compared to WT cells. N = 4 samples for each condition. Unpaired Student's t-test, ***$p < 0.001$, compared with WT samples. Data are mean ± SEM.

Unpaired Student's t-test in (B, C, D and F), *$p < 0.05$, **$p < 0.01$, ***$p < 0.001$, compared with WT samples. Data are mean ± SEM.

Figure S13

A

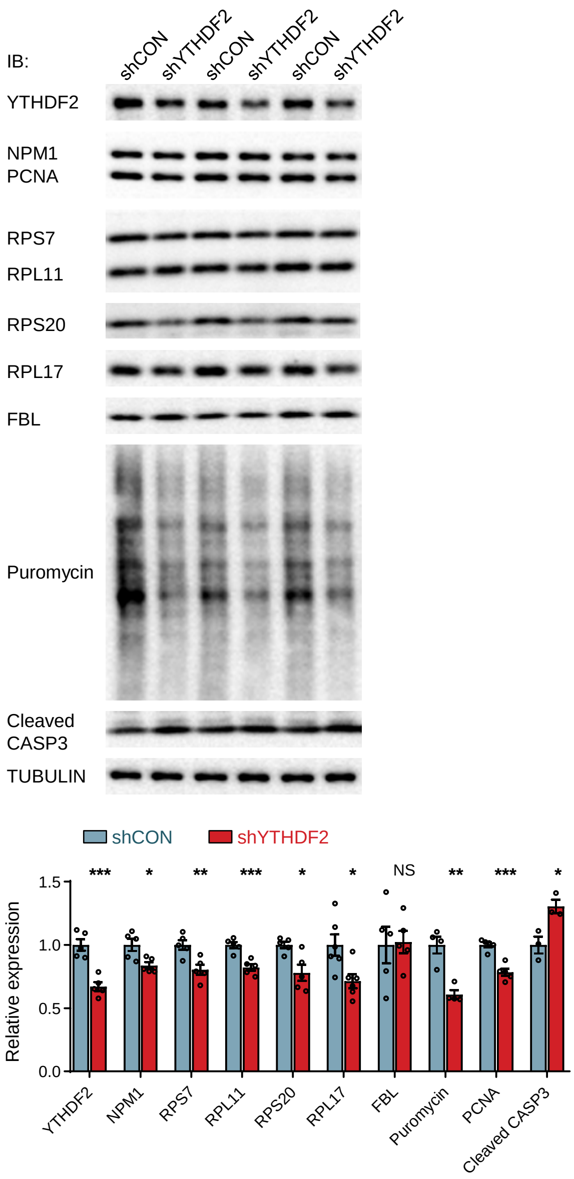

B

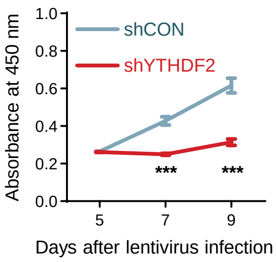

Fig. S13. YTHDF2 deficiency in NPCs impairs ribosome biogenesis, compromises global protein translation, reduces cell proliferation, and enhances apoptosis.

(**A**) Western blot analysis assessing the protein levels of ribosome-associated proteins, puromycin-labeled protein, cell proliferation marker PCNA and apoptosis marker cleaved CASP3 in cultured NPCs infected with lentivirus expressing either shCON or shYTHDF2. N = 3-6 samples for each condition.

(**B**) Growth curves of cultured NPCs infected with lentivirus expressing shCON or shYTHDF2. Cell numbers were determined by CCK8 assay. The assays were repeated two times, and one representative result is shown.

Unpaired Student's t-test in (A and B), $*p < 0.05$, $**p < 0.01$, $***p < 0.001$, compared with control cells. Data are mean ± SEM.

Figure S14

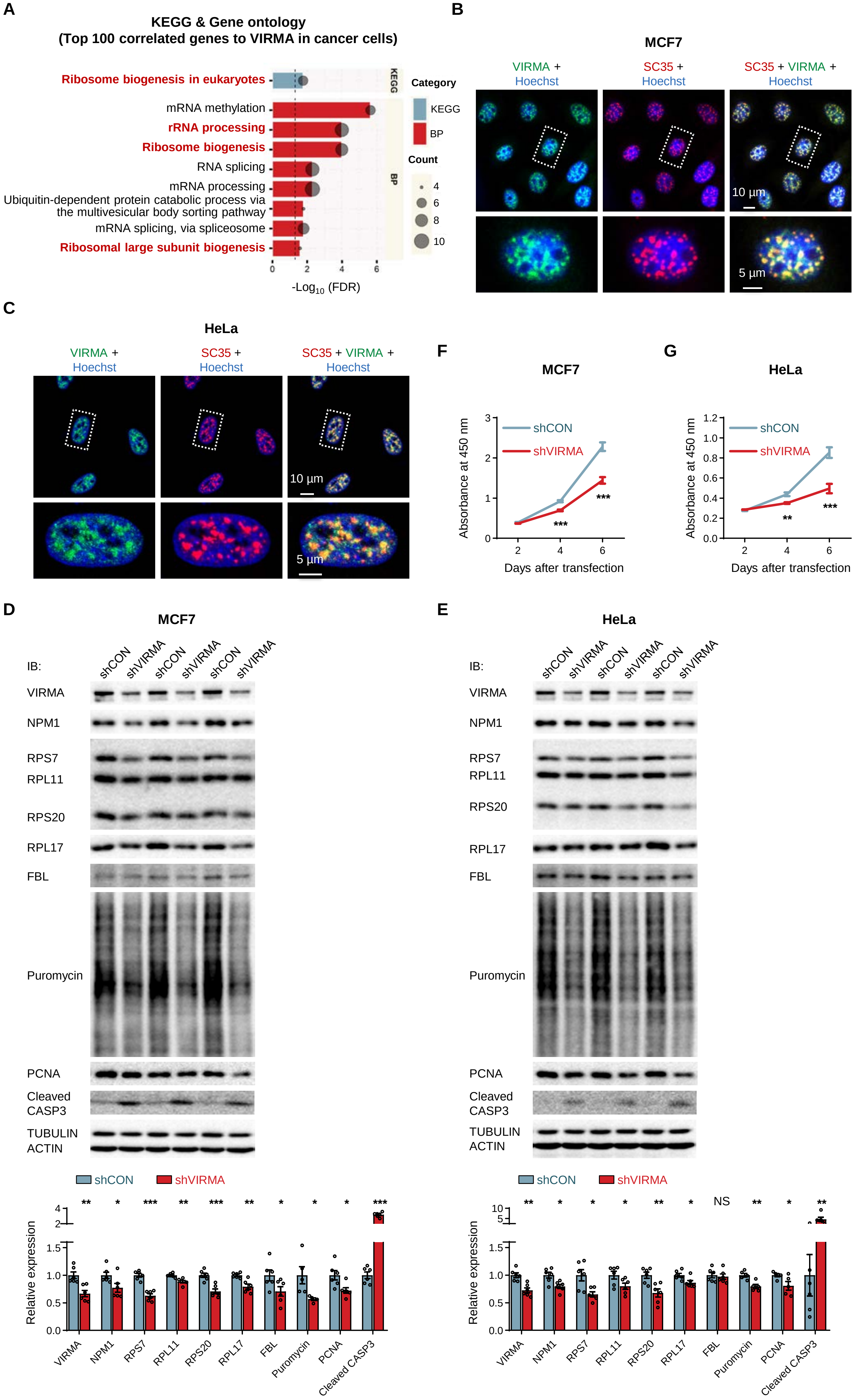
A
KEGG & Gene ontology
(Top 100 correlated genes to VIRMA in cancer cells)
Ribosome biogenesis in eukaryotes
mRNA methylation
rRNA processing
Ribosome biogenesis
RNA splicing
mRNA processing
Ubiquitin-dependent protein catabolic process via the multivesicular body sorting pathway
mRNA splicing, via spliceosome
Ribosomal large subunit biogenesis
KEGG
BP
Category
KEGG
BP
Count
4
6
8
10
-Log10 (FDR)
B
MCF7
VIRMA + Hoechst
SC35 + Hoechst
SC35 + VIRMA + Hoechst
10 µm
5 µm
C
HeLa
VIRMA + Hoechst
SC35 + Hoechst
SC35 + VIRMA + Hoechst
10 µm
5 µm
F
MCF7
G
HeLa
shCON
shVIRMA
Absorbance at 450 nm
Days after transfection
***
**
D
MCF7
E
HeLa
IB:
shCON
shVIRMA
VIRMA
NPM1
RPS7
RPL11
RPS20
RPL17
FBL
Puromycin
PCNA
Cleaved CASP3
TUBULIN
ACTIN
Relative expression
NS

Fig. S14. VIRMA deficiency in proliferating cancer cells impairs ribosome biogenesis, compromises global protein translation, reduces cell proliferation and enhances apoptosis.

(**A**) KEGG pathway and GO analyses of the top 100 genes whose dependency scores were correlated with that of VIRMA in hundreds of cancer cell lines (CRISPR, DepMap 22Q2 Public+Score, Chronos). Significantly enriched terms (FDR < 0.05, determined by Fisher's Exact test in DAVID) are listed and ranked by FDR value. Enriched terms related to ribosome biogenesis are highlighted in red. BP, biological processes.

(**B** and **C**) Immunostaining showing VIRMA localization to SC35-positive nuclear speckles in MCF7 (B) and HeLa (C) cells.

(**D** and **E**) Western blot analysis assessing the protein levels of ribosome-associated proteins, puromycin-labeled protein, cell proliferation marker PCNA and apoptosis marker cleaved CASP3 in MCF7 (D) and HeLa (E) cells transfected with shCON or shVIRMA plasmids. N = 5-6 samples for each condition.

(**F** and **G**) Growth curves of MCF7 (F) or HeLa (G) cells transfected with shCON or shVIRMA plasmids. Cell numbers were determined by CCK8 assay. The assays were repeated two times, and one representative result is shown.

Unpaired Student's t-test in (D to G), *$p < 0.05$, **$p < 0.01$, ***$p < 0.001$, compared with control cells. Data are mean ± SEM.

Figure S15. Uncropped western blots.

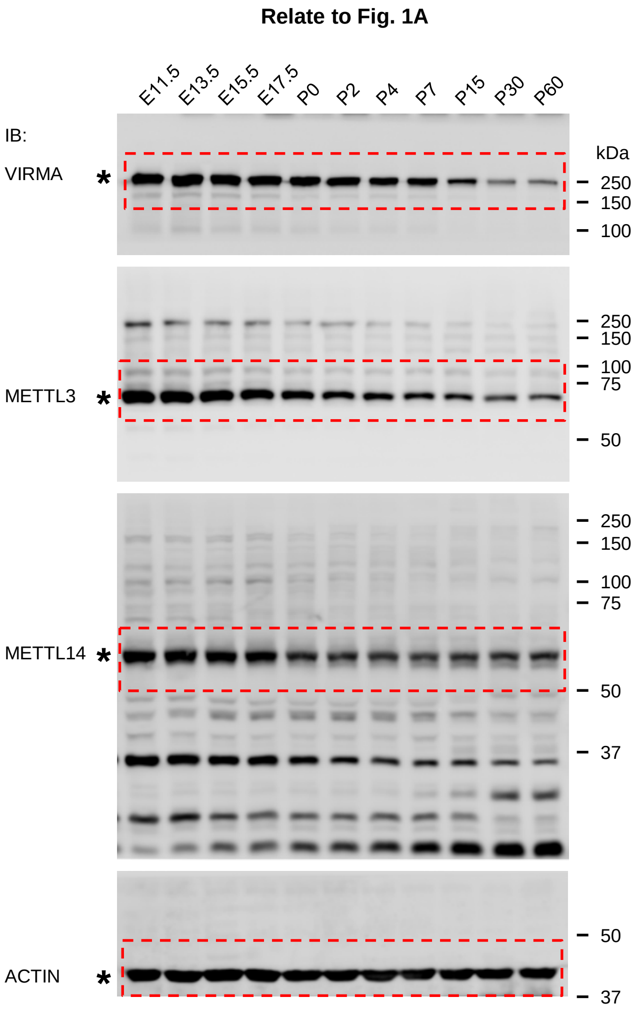

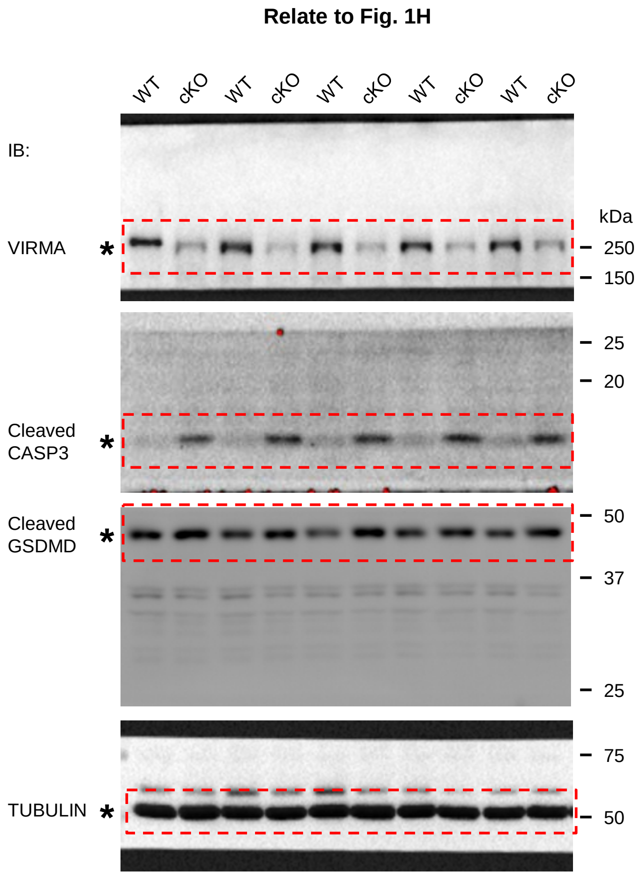

Figure S15. Uncropped western blots. (continued)

Relate to Fig. 2B

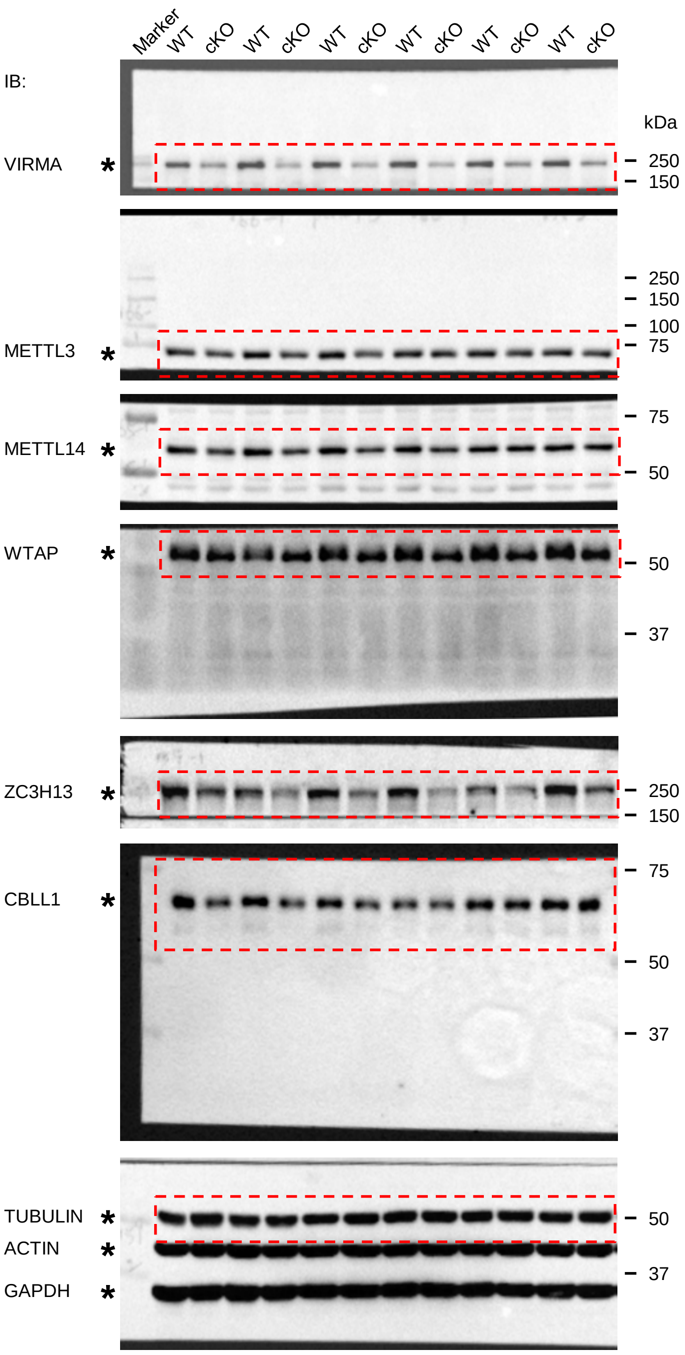

Relate to Fig. 3E

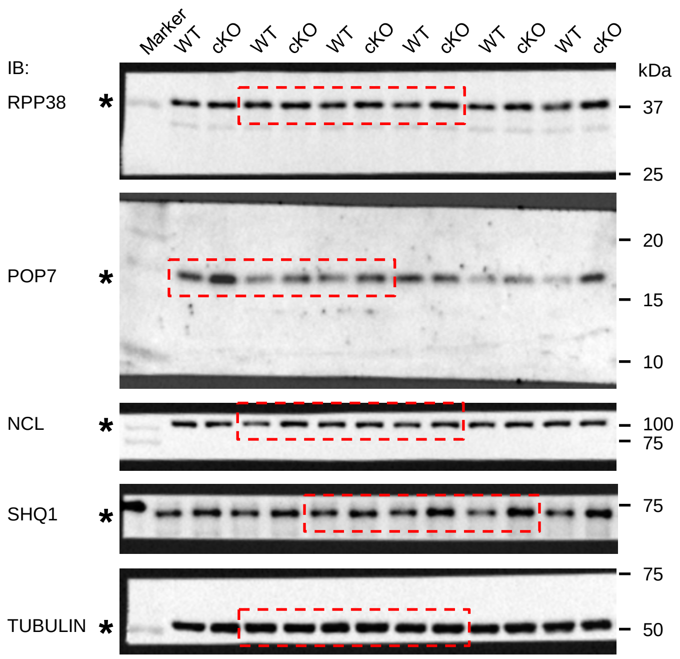

Figure S15. Uncropped western blots. (continued)

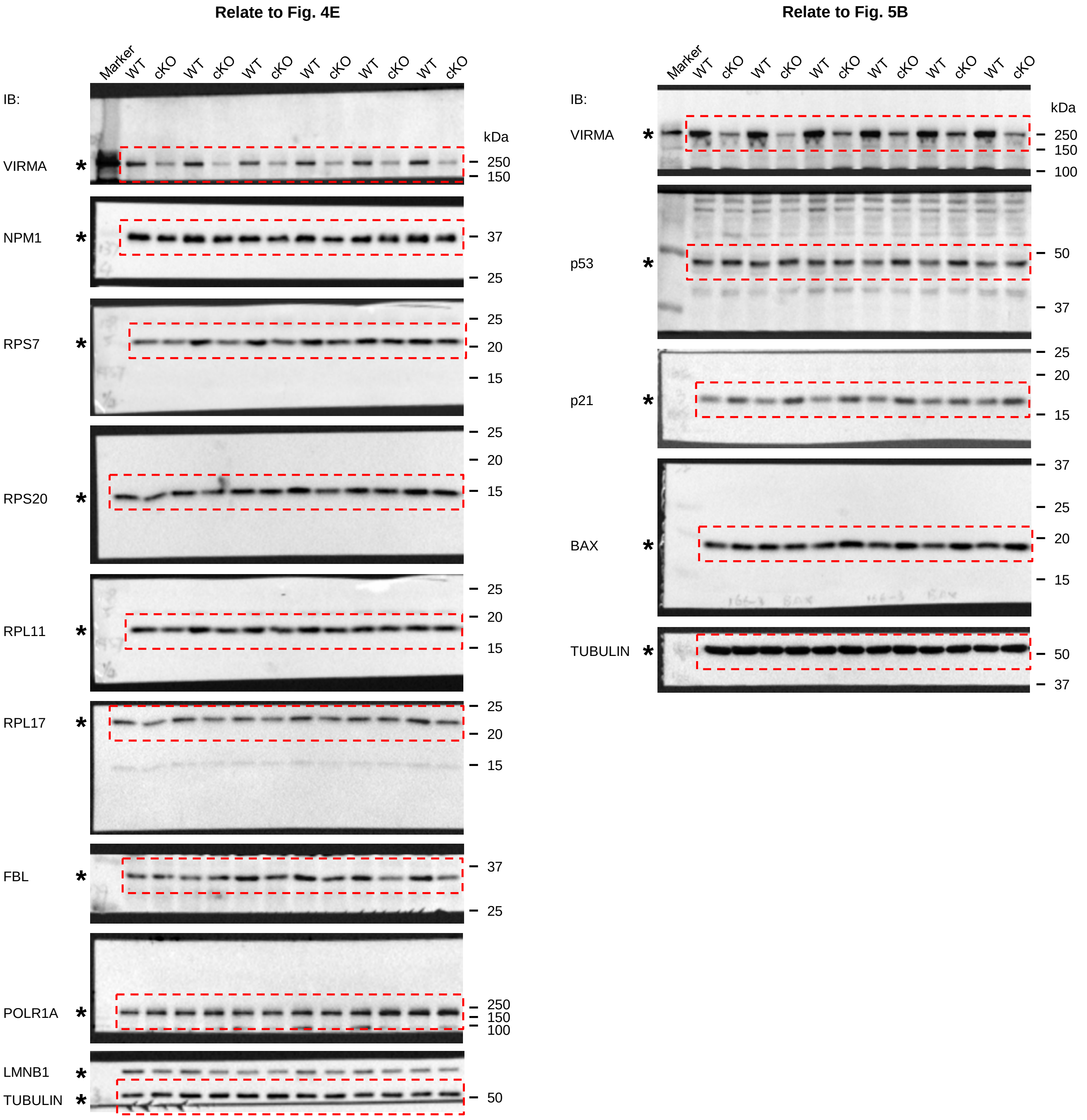

Figure S15. Uncropped western blots. (continued)

Relate to Fig. 5D

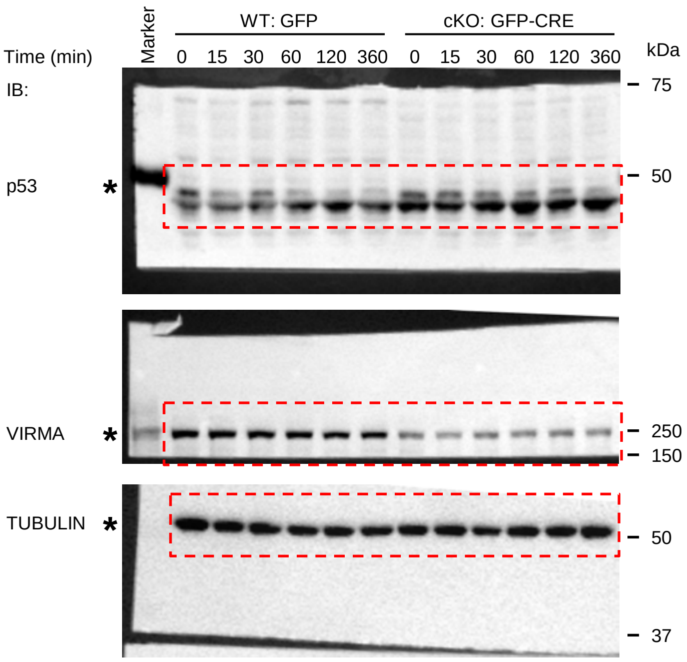

Relate to Fig. 5E

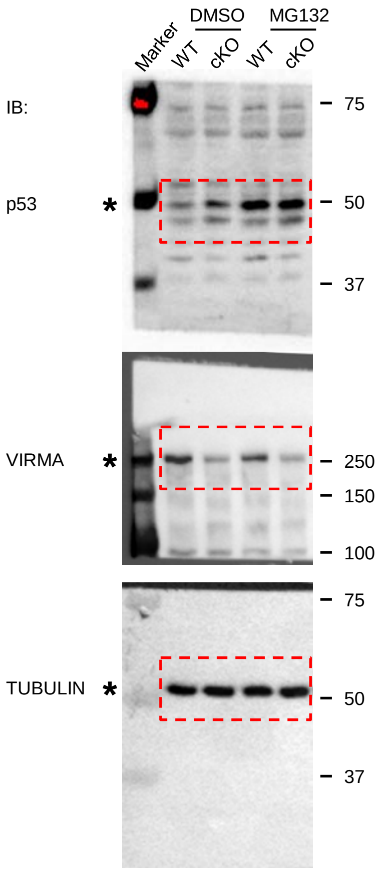

Figure S15. Uncropped western blots. (continued)

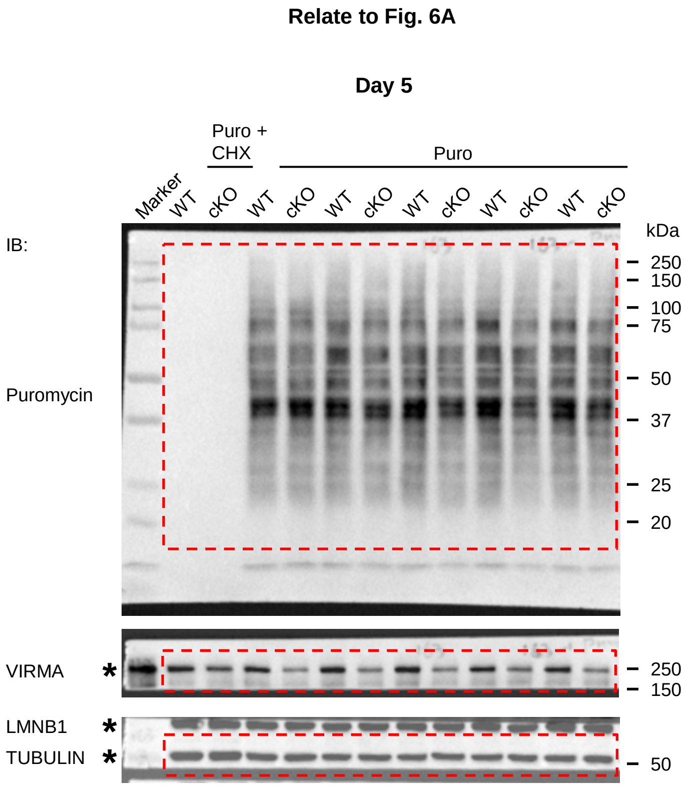

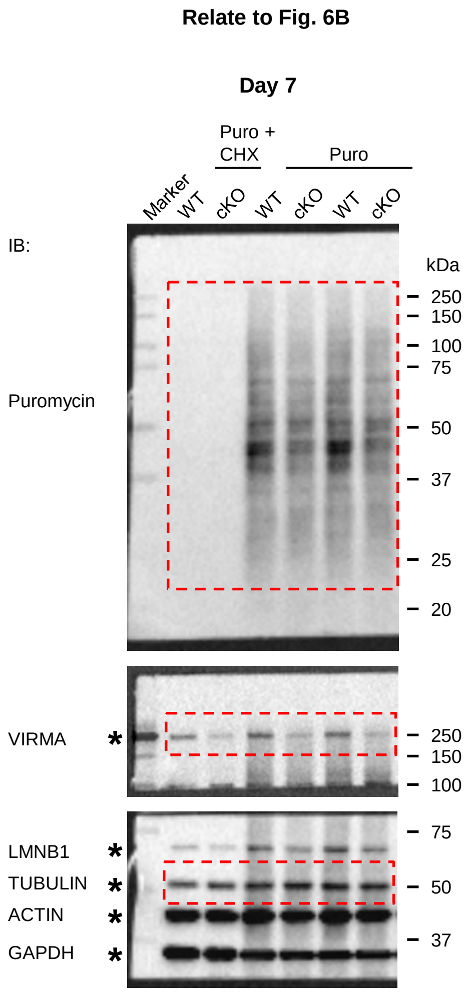

Figure S15. Uncropped western blots. (continued)

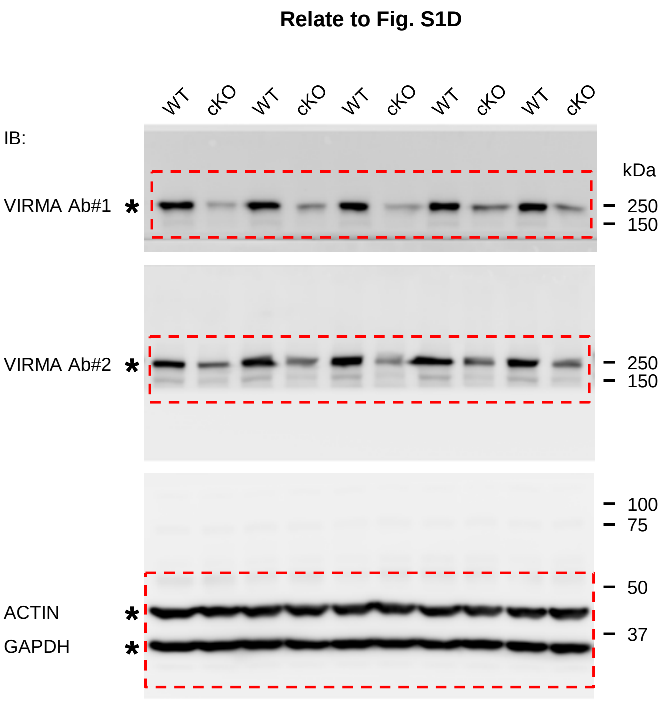

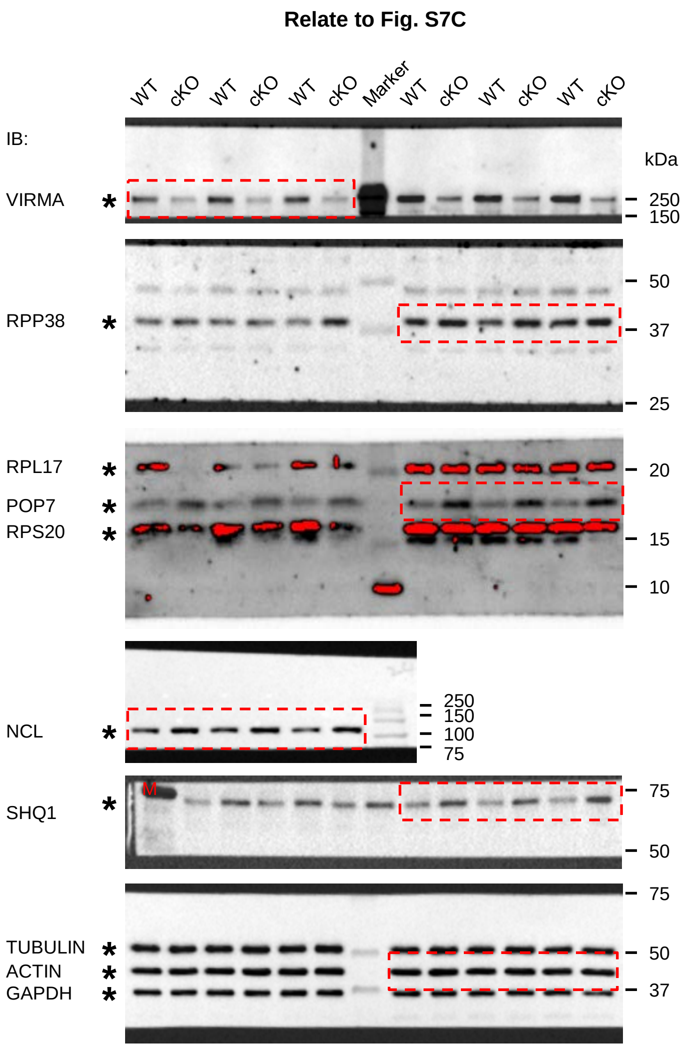

Figure S15. Uncropped western blots. (continued)

Relate to Fig. S8E

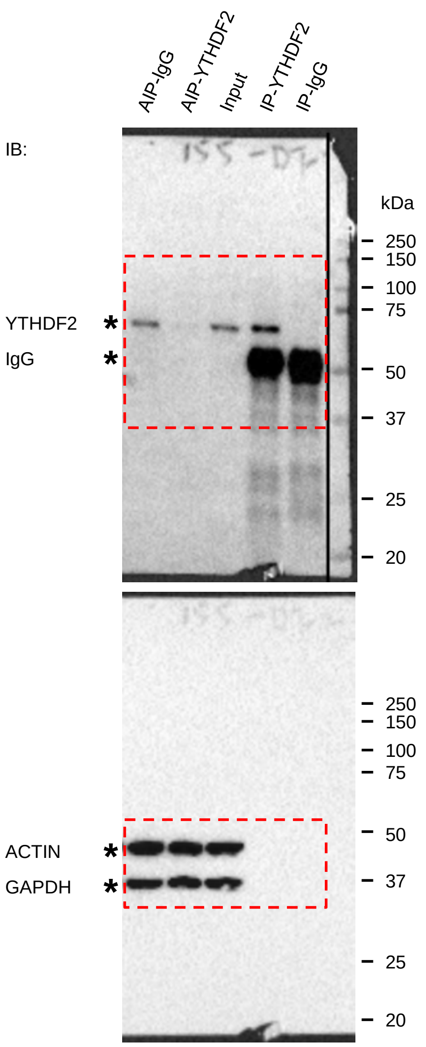

Relate to Fig. S10A

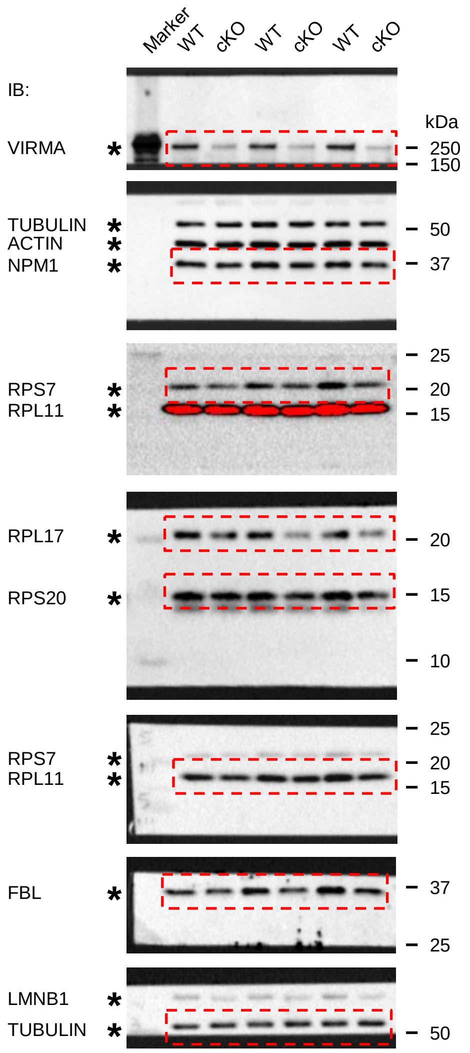

Figure S15. Uncropped western blots. (continued)

Relate to Fig. S13A

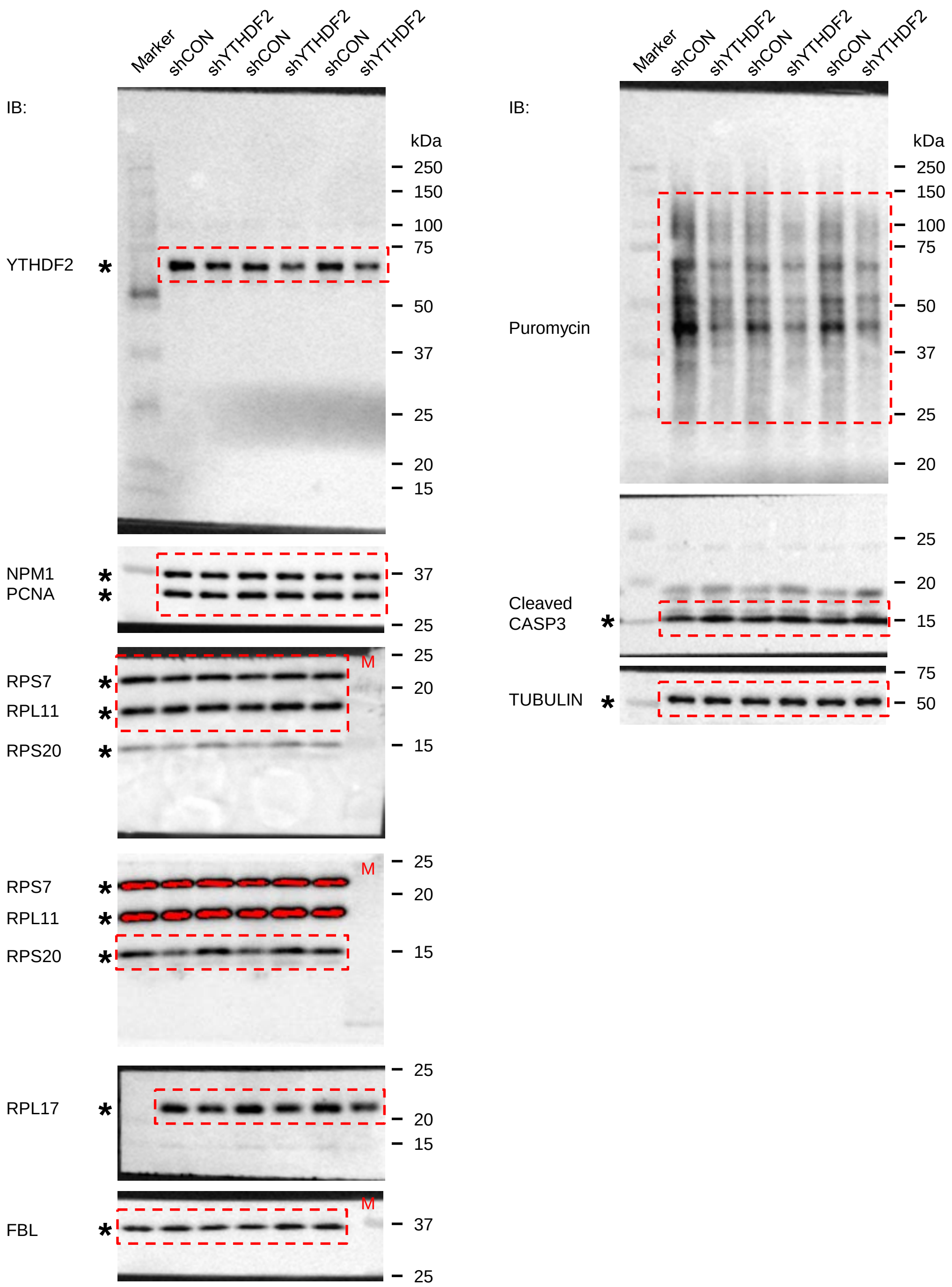

Figure S15. Uncropped western blots. (continued)

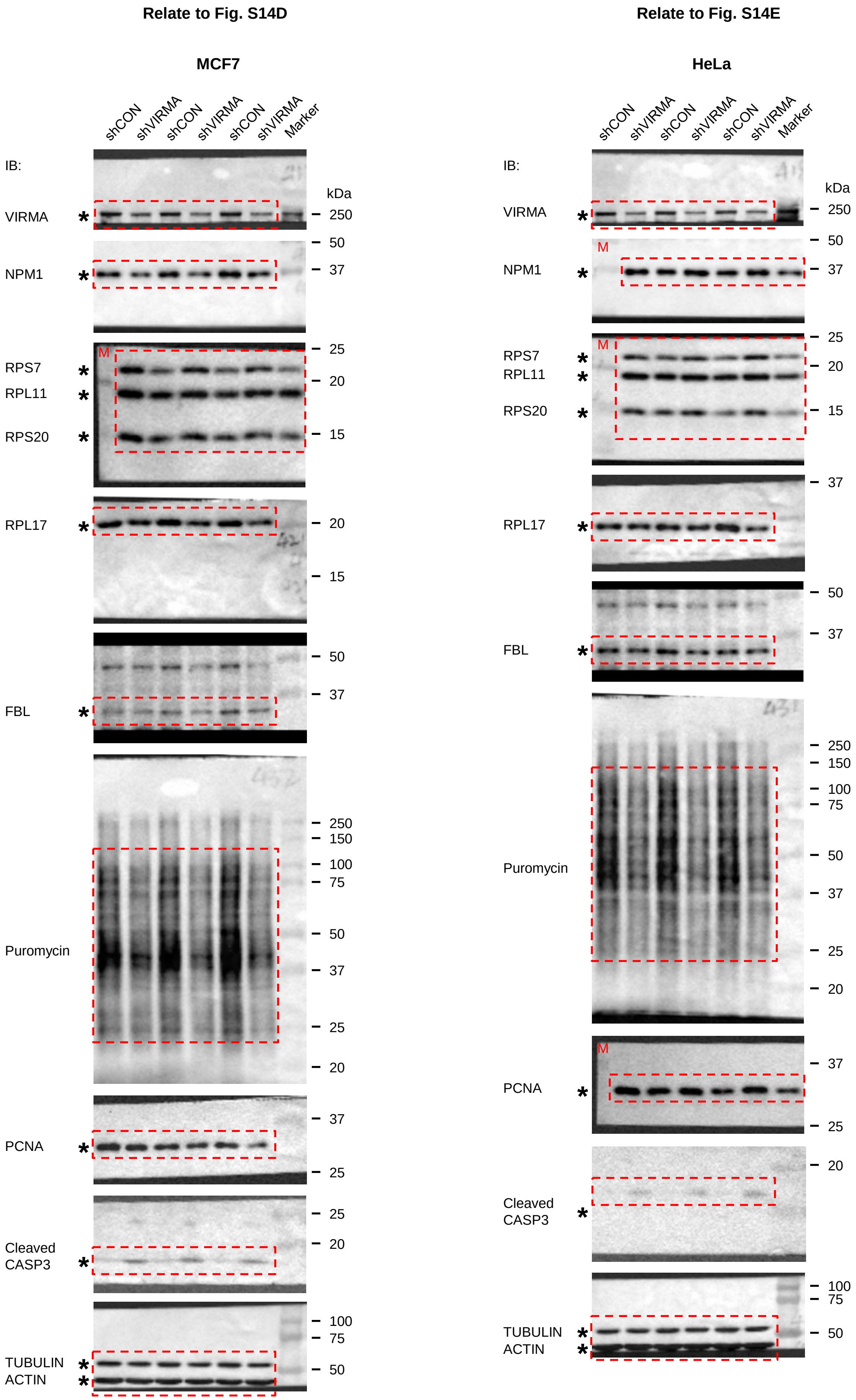

Fig. S15. Uncropped western blots.

Images of the target bands were merged with images of the molecular weight markers (where available) to show the boundaries of the blots. Red dashed boxes indicate the regions that were cut out for display, and the target bands are indicated with asterisks (*).

Figure S16

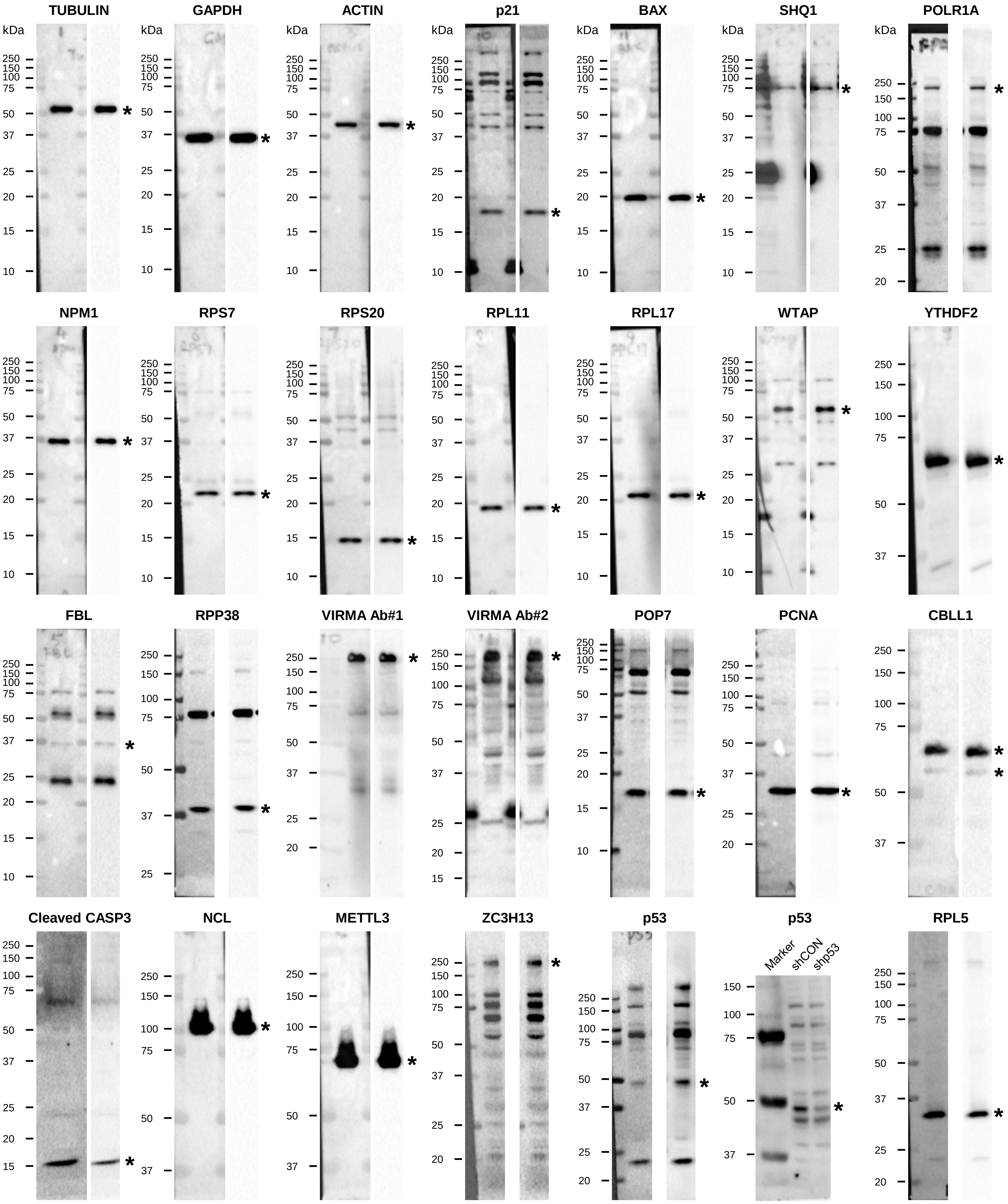
TUBULIN
GAPDH
ACTIN
p21
BAX
SHQ1
POLR1A
kDa
NPM1
RPS7
RPS20
RPL11
RPL17
WTAP
YTHDF2
FBL
RPP38
VIRMA Ab#1
VIRMA Ab#2
POP7
PCNA
CBLL1
Cleaved CASP3
NCL
METTL3
ZC3H13
p53
p53
RPL5
Marker
shCON
shp53

Fig. S16. Antibody tests on western blot strips.

For each antibody, the left panel shows images of the target bands merged with images of the molecular weight markers (where available). The right panel displays images of the target bands alone. The target bands are indicated with asterisks (*).

Supplementary Files:

Table S1. (separate file)
m^6A peaks identified from the forebrain of WT and VIRMA cKO mice at E13.5.

Table S2. (separate file)
Differential m^6A peaks identified from the forebrain of VIRMA cKO mice compared with WT mice at E13.5.

Table S3. (separate file)
RNA-seq analysis of forebrain from WT and VIRMA cKO mice at E13.5.

Table S4. (separate file)
Differentially expressed proteins identified from the forebrain of WT and VIRMA cKO mice at E13.5.

Table S5. (separate file)
List of primers used for RT-qPCR.
